# NF-κB determines Paneth versus goblet cell fate decision in the small intestine

**DOI:** 10.1242/dev.199683

**Published:** 2021-11-09

**Authors:** Cristina Brischetto, Karsten Krieger, Christian Klotz, Inge Krahn, Séverine Kunz, Marina Kolesnichenko, Patrick Mucka, Julian Heuberger, Claus Scheidereit, Ruth Schmidt-Ullrich

**Affiliations:** 1 Signal Transduction in Tumor Cells, Max-Delbrück-Center for Molecular Medicine, Robert-Rössle-Str. 10, 13092 Berlin, Germany; 2 Unit for Mycotic and Parasitic Agents and Mycobacteria, Robert Koch-Institute (RKI), 13353 Berlin, Germany; 3 CF Electron Microscopy, Max-Delbrück-Center for Molecular Medicine, Robert-Rössle-Str. 10, 13092 Berlin, Germany; 4 Department of Gastroenterology, Infectious Diseases and Rheumatology, Charité-Universitätsmedizin Berlin, Hindenburgdamm 30, 12203 Berlin, Germany; 5 Signal Transduction in Development and Cancer, Max-Delbrück-Center for Molecular Medicine, Robert-Rössle-Str. 10, 13092 Berlin, Germany; 6 Medical Department, Division of Gastroenterology and Hepatology, Charité University Medicine, 13353 Berlin, Germany

**Keywords:** NF-κB, Intestine, Paneth cells, Goblet cells, Stem cells, Epithelial self-renewal, Mouse

## Abstract

Although the role of the transcription factor NF-κB in intestinal inflammation and tumor formation has been investigated extensively, a physiological function of NF-κB in sustaining intestinal epithelial homeostasis beyond inflammation has not been demonstrated. Using NF-κB reporter mice, we detected strong NF-κB activity in Paneth cells, in ‘+4/+5’ secretory progenitors and in scattered Lgr5^+^ crypt base columnar stem cells of small intestinal (SI) crypts. To examine NF–κB functions in SI epithelial self-renewal, mice or SI crypt organoids (‘mini-guts’) with ubiquitously suppressed NF-κB activity were used. We show that NF-κB activity is dispensable for maintaining SI epithelial proliferation, but is essential for *ex vivo* organoid growth. Furthermore, we demonstrate a dramatic reduction of Paneth cells in the absence of NF-κB activity, concomitant with a significant increase in goblet cells and immature intermediate cells. This indicates that NF-κB is required for proper Paneth versus goblet cell differentiation and for SI epithelial homeostasis, which occurs via regulation of Wnt signaling and Sox9 expression downstream of NF-κB. The current study thus presents evidence for an important role for NF-κB in intestinal epithelial self-renewal.

## Abstract (precis)

**Summary:** The transcription factor NF-κB, together with downstream Wnt and Sox9, is required for Paneth and goblet cell fate decisions and for maintenance of the small intestinal stem cell niche.

## INTRODUCTION

The single-layered epithelium of the small intestine (SI) self-renews approximately every 5 days and thus displays the fastest turnover in mammalian tissues. The continuous and intense self-renewal of the various intestinal epithelial cell (IEC) types is guaranteed by specialized stem cells. These reside in a particular niche, the so-called crypts of Lieberkühn, located at the bottom of the villus, which itself constitutes the differentiated compartment of the SI (reviewed by Barker et al., 2008; Clevers, 2013; Gehart and Clevers, 2019). Both compartments are connected by a clearly restricted transition zone composed of transit-amplifying (TA) progenitor cells (reviewed by Barker et al., 2008, 2010; Carulli et al., 2014). The villus consists of absorptive enterocytes and of three types of secretory cell types: the enteroendocrine, tuft and goblet cells (Barker et al., 2010). A fourth cell type of the secretory lineage, Paneth cells, is found in crypts, intermingled with the highly proliferative intestinal stem cells (ISCs), also denominated crypt base columnar stem cells (CBCs) (Clevers and Bevins, 2013). Maintenance of CBCs and differentiation of Paneth cells strongly rely on active canonical Wnt signaling (Pinto et al., 2003; van Es et al., 2005; Fevr et al., 2007; Farin et al., 2012), which is potentiated by the R-spondin receptor Lgr5 (leucine rich repeat containing G protein coupled receptor 5) specifically expressed on CBCs (Barker et al., 2007; Koo and Clevers, 2014).

So far, the role of the transcription factor NF-κB in intestinal homeostasis has been studied in the contexts of inflammation and of tumorigenesis (reviewed by Pasparakis, 2009; Ben-Neriah and Karin, 2011; Taniguchi and Karin, 2018). IEC-specific deletion of either NF-κB subunits or components of the upstream IKK (IκB kinase) complex, but also constitutively elevated IKK/NF-κB activity in the intestinal epithelium, consistently resulted in local infiltration of immune cells and inflammation (such as in inflammatory bowel disease), increased IEC apoptosis and tissue damage (Chen et al., 2003; Greten et al., 2004; Nenci et al., 2007; Steinbrecher et al., 2008; Guma et al., 2011; Vlantis et al., 2011; Shaked et al., 2012; Schwitalla et al., 2013; Vereecke et al., 2014; Vlantis et al., 2016; Mikuda et al., 2020). Although tumor formation was also observed in IKK/NF-κB knockout (KO) mice to some degree, high incidence of colitis and spontaneous inflammation-associated tumor formation (colitis-associated cancer) was significantly enhanced in mice with constitutively active IKK/NF-κB in IECs (Shaked et al., 2012; Mikuda et al., 2020). Furthermore, it has been shown that NF-κB is required for mucosal innate immunity, barrier functions, and immune resistance to the intestinal microbiome, as well as for the formation of Peyer's patches, which are part of the gut-associated lymphoid tissue in the small intestine (Schmidt-Ullrich et al., 2001; Yilmaz et al., 2003; Zaph et al., 2007; Pasparakis, 2008). These studies suggest that intestinal NF-κB activation must be a well-balanced and strictly controlled process.

We have previously shown that NF-κB controls fetal hair follicle induction and participates in a significant manner in the regulation of the postnatal hair cycle, which is responsible for periodic renewal of hair follicles (Schmidt-Ullrich et al., 2006; Zhang et al., 2009; Krieger et al., 2018). Similar to intestinal epithelial self-renewal, the hair cycle strictly depends on actively cycling Lgr5-positive stem cells located in the bulge region of the hair follicle (reviewed by Fuchs, 2007; Blanpain and Fuchs, 2014; Hsu et al., 2014). We have demonstrated that in hair follicles NF-κB directly regulates the expression of markers that are essential for stem cell maintenance and activation (Zhang et al., 2009; Tomann et al., 2016; Krieger et al., 2018). Here, in the SI epithelium we observed strong NF-κB activity in Paneth cells, in +4/+5 secretory progenitors and occasionally in CBCs. This prompted us to examine more closely the role of NF-κB in IEC self-renewal. The results of the current study were obtained from a mouse model with ubiquitously suppressed NF-κB activity (here referred to as Δ*N*; Schmidt-Ullrich et al., 2001). Simultaneous suppression of NF-κB in IECs and surrounding tissues did not result in inflammation, which is observed persistently in epithelial-specific NF-κB/IKK KOs (see above and Pasparakis et al., 2002; Stratis et al., 2006; Grinberg-Bleyer et al., 2015), and did not show any changes in IEC proliferation or apoptosis. However, our data reveals a previously unknown role for NF-κB in Paneth versus goblet cell fate decisions.

## RESULTS

### Strong NF-κB activity is observed in murine SI in Paneth cells and secretory progenitors at position +4/+5

**Fig. 1.**
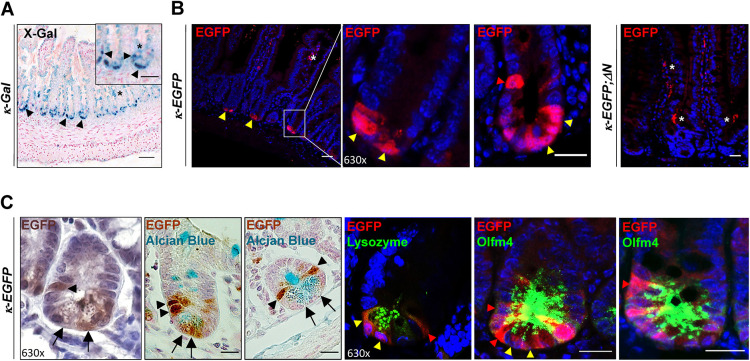
**Cell type-specific NF-κB activity in crypts of the small intestine.** (A) Technovit section of X-gal-stained PSIs of *κ-Gal* reporter mice (*n*=3). Inset shows magnification with X-Gal-stained cells in the bottom of the crypts and in +4/+5 position (black arrowheads). Arrowheads indicate specific X-Gal staining in crypts, asterisks diffuse, nonspecific staining. Scale bars: 50 μm (main panel); 10 μm (inset). (B) Indirect immunofluorescence using an anti-EGFP antibody on PSI sections of *κ-EGFP* and *κ-EGFP;*Δ*N* mice (representative of *n*=3 mice per group). Yellow arrowheads indicate EGFP expression/NF-κB activity, asterisk marks nonspecific staining in villi. Scale bars: 50 μm (far left and far right); 20 μm (middle). (C) EGFP IHC [DAB (brown) alone and DAB plus Alcian Blue staining; left panels] and immunofluorescence co-staining of EGFP with anti-lysozyme or anti-Olfm4 antibodies on PSI sections of *κ-EGFP* mice (*n*=3). Black arrows and yellow arrowheads point to Paneth cells; black or red arrowheads indicate EGFP expression/NF-κB activity in ISCs, mainly in +4 and +5 positions. Scale bars: 20 μm. Nuclear counterstain: DAPI (blue).

To analyze NF-κB activity in the small intestine under physiological conditions, NF-κB reporter mouse lines using either β-galactosidase (*κ-Gal*; Fig. 1A) or EGFP (*κ-EGFP*; Fig. 1B,C) expression as a read-out for NF-κB activity were examined 8-12 weeks after birth. In the adult murine SI, strong β-galactosidase or EGFP expression was confined to the crypts (Fig. 1A,B). Specificity of the signal was determined using *κ-EGFP;*Δ*N* mice, in which NF-κB activity is ubiquitously suppressed and in which EGFP expression was absent in SI crypts (Fig. 1B). NF-κB activity in SI crypts was further validated by *in situ* hybridization (ISH) using a riboprobe of *Nfkbia*, a bona fide target gene of NF-κB that encodes IκBα (inhibitor of NF-κB α) (Le Bail et al., 1993). As expected, endogenous *Nfkbia* mRNA expression was only detected in SI crypts (Fig. S1A, upper panel, control).

Co-staining of EGFP with Alcian Blue, the Paneth cell marker lysozyme or the ISC marker Olfm4 (olfactomedin 4; see also van der Flier et al., 2009) in the SI of *κ-EGFP* mice identified SI crypt cells with NF-κB activity mainly as Paneth cells, but also as cells at position +4/+5 and occasional CBCs (Fig. 1C). Cells at position +4/+5 above the SI crypt have previously been identified as potential secretory precursors (Stamataki et al., 2011; van Es et al., 2012; Buczacki et al., 2013; Tetteh et al., 2015). These results suggest a physiological function for NF-κB in Paneth cells and in progenitors.

### IEC proliferation and cell death remain unaltered in Δ*N* mice

To investigate further the functions of NF-κB in the SI epithelium, mice with ubiquitous suppression of NF-κB activity (Δ*N*) or mice with IEC-restricted NF-κB inhibition (*villinCRE*×*floxed* Δ*N*: hereafter referred to as *Villin-*Δ*N*) were examined. Suppressed NF-κB activity in Δ*N* mice was verified by loss of *Nfkbia* mRNA expression (Fig. S1A). More importantly, in Δ*N* and *floxed* Δ*N* mice the truncated form of the NF-κB inhibitor IκBα (ΔN) was integrated in-frame into the β-catenin (*Ctnnb1*) locus, which does not impair expression and activity of the β-catenin protein (Fig. S1B,C; Schmidt-Ullrich et al., 2001). Thus, the phenotype observed here is a result of ubiquitous suppression of NF-κB activity.

**Fig. 2.**
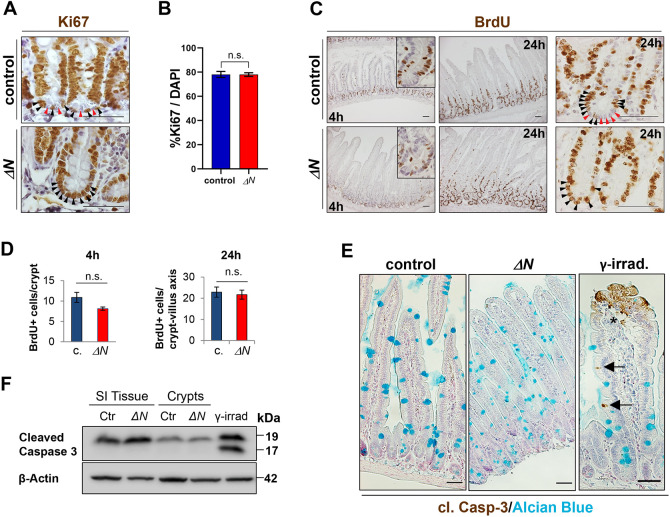
**Unaltered IEC proliferation and cell death rate in Δ*N* mice compared with controls.** (A) Anti-Ki67 antibody staining on PSI sections of Δ*N* and control mice indicating proliferative cells. *n*=3 per group. (B) Quantification of average numbers of Ki67^+^ cells as a percentage of total DAPI-stained cells. *n*=3 per group, *n*>20 crypts per mouse. (C) Anti-BrdU antibody staining on PSI sections of Δ*N* and control mice indicating BrdU incorporation 4 or 24 h after injection. Insets show magnified images of crypts. In A and C, in crypts of controls proliferating ISCs (black arrowheads) are intermingled with Paneth cells (red arrowheads). (D) Quantification of BrdU^+^ cells per crypt (4 h) and per crypt-villus axis (24 h) in control or Δ*N* mice. *n*=3 per group, *n*≥20 crypts per mouse. (E) Representative IHC images of anti-cleaved caspase 3 and Alcian Blue staining on PSI sections of Δ*N* and control (*n*=3 per group) mice, and of a tissue sample of γ-irradiated control mice (see Kolesnichenko et al., 2021). Black arrows point to caspase 3-positive (apoptotic) cells in villi of irradiated controls. Asterisk indicates nonspecific staining due to handling. (F) Analysis of cleaved caspase-3 protein expression. Total PSI and PSI crypt whole-cell extracts of control and Δ*N* mice (*n*=3 per group) were used for SDS-PAGE western blotting. β-Actin was used as loading control. Scale bars: 50 µm (A,C); 200 μm (E). In B,D, error bars represent s.e.m.; n.s., not significant (unpaired Welch's *t*-test).

Among others, NF-κB can regulate cell proliferation and death. Ki67 staining showed an altered distribution of proliferating cells in SI crypts of Δ*N* mice (Fig. 2A). In contrast to controls, all crypt base cells were positive for Ki67 and may correspond to TA cells that migrate into the crypt and/or other immature cell types (Fig. 2A). To analyze the proliferation rate further, we used 5-bromo-2′-desoxyuridine (BrdU) treatment for either 4 h to detect alterations in the cell cycle or for 24 h to reveal migration defects within the intestinal epithelium (Fig. 2C). Quantitative analysis of SI sections stained with either anti-Ki67 or anti-BrdU antibodies did not show any alteration in numbers of BrdU- or Ki67-positive cells in Δ*N* crypts compared with controls (for 4 h BrdU: *P*=0.1; for 24 h BrdU: *P*=0.38; for Ki67: *P*=0.96; Fig. 2B,D). Similar to Ki67 staining, in controls we observed BrdU-positive CBCs adjacent to post-mitotic Paneth cells whereas in Δ*N* mice crypts were entirely occupied with BrdU-positive cells (Fig. 2C, right panels, 24 h). These data demonstrate that suppression of NF-κB activity does not change the overall proliferation rate in the intestinal epithelium.

A recent study found that mice with intestinal epithelial knockout of the IKK subunit IKKγ (also known as NEMO) or subunits of NF-κB showed a significant increase in apoptosis of IECs, including Paneth cells (Vlantis et al., 2016). However, neither terminal deoxynucleotidyl transferase dUTP nick end labeling (TUNEL) nor cleaved caspase 3 staining showed an elevated number of apoptotic cells in the SI epithelium or in crypts of Δ*N* mice compared with controls (Fig. S2A, Fig. 2E,F). Importantly, no increase in inflammatory cytokines was observed in intestines of Δ*N* mice (Fig. S1D), in contrast to mice with constitutively elevated NF-κB activity in IECs (*IκBα^IEC-KO^*) (Fig. S1E), which exhibited increased inflammation, proliferation and apoptosis (Mikuda et al., 2020). Analyses of selected inflammatory bowel disease markers that depend on NF-κB activity also showed that in comparison with *IκBα^IEC-KO^* mice and controls, expression of these NF-κB targets was significantly reduced or absent in Δ*N* mice (Fig. S1F) (Mikuda et al., 2020).

### Increase in goblet cells in the SI of Δ*N* mice

**Fig. 3.**
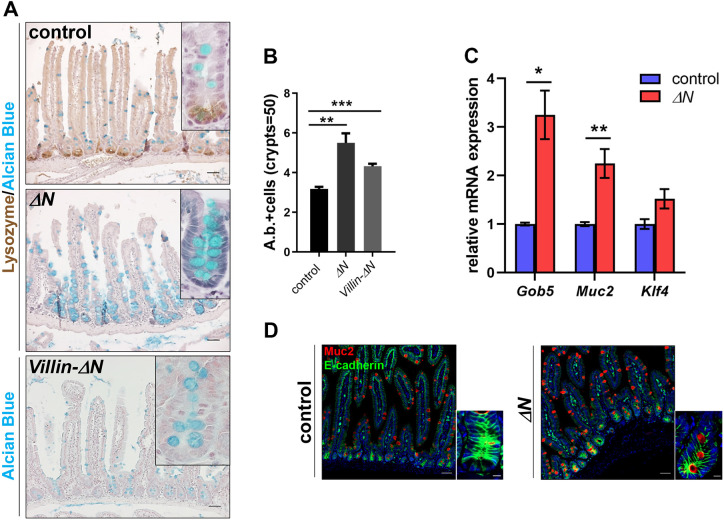
**Increased numbers of goblet cells in Δ*N* mice.** (A) IHC on PSI sections of control, Δ*N* and *Villin-*Δ*N* mice (*n*=3 per group) stained with either Alcian Blue alone or together with an anti-lysozyme antibody. Scale bars: 50 µm. Insets show magnified images of crypts. (B) Quantification of the average number of Alcian Blue-positive cells per crypt. **P<0.01, ***P<0.001 (unpaired Welch's *t*-test). Error bars represent s.e.m. *n*=5 per group, *n*=50 crypts per sample. (C) qRT-PCR for mature goblet cell markers *Gob5*, *Muc2* and *Klf4* using RNA from PSIs of Δ*N* and control mice (*n*=5 per group). *P<0.05, **P<0.01 (multiple *t*-test with Bonferroni correction). (D) Immunofluorescence using anti-Muc2 and anti-E-cadherin (cadherin 1) antibody staining on sections of PSI of control and Δ*N* mice (*n*=3 per group). Nuclear counterstain: DAPI (blue). Scale bars: 50 µm (A,D, main panels); 10 µm (insets).

Alcian Blue staining, which detects acidic mucopolysaccharides, revealed a dramatic increase of Alcian Blue-positive cells in SI, particularly in the crypts of Δ*N* and *villin-*Δ*N* mice compared with controls (Fig. 3A). This was observed in all three major segments of the small intestine: the duodenum [proximal small intestine (PSI)], jejunum [medium SI (MSI)] and ileum [distal SI (DSI)] (Fig. 3A; Fig. S2B). Quantification by counting Alcian Blue-positive cell numbers/crypt unit verified this finding (1.9-fold increase; Fig. 3B). Note that we did not observe any abnormalities related to inflammatory processes in the SI of *villin-*Δ*N* mice (data not shown) and increased goblet cell numbers were also never reported in any of the mice with IEC-specific deletion of IKK/NF-κB. Alcian Blue usually stains mature goblet cells, but also immature intermediate Paneth/goblet cell precursors, which prompted us to analyze goblet cell markers. mRNA expression of the goblet cell markers *Gob5* (*Clca1*) and *Muc2* was significantly increased in Δ*N* mice, whereas expression of *Klf4* (Krüppel-like factor 4) remained similar to controls (Fig. 3C). Accordingly, Muc2 protein expression was also elevated in SIs of Δ*N* mice (Fig. 3D). Muc2 and Gob5 are markers for mature goblet cells and Klf4 controls goblet cell fate decisions but is not required for final differentiation (Katz et al., 2002; Garg et al., 2007; Ghaleb et al., 2011; Pellegrinet et al., 2011). These results suggest that Klf4 may act upstream of NF-κB activation in secretory cell fate decisions, and confirm increased amounts of goblet cells in SIs and possibly the presence of immature intermediate Paneth/goblet cell precursors in the crypts of Δ*N* mice.

### Loss of Paneth cells in SI crypts of Δ*N* mice

**Fig. 4.**
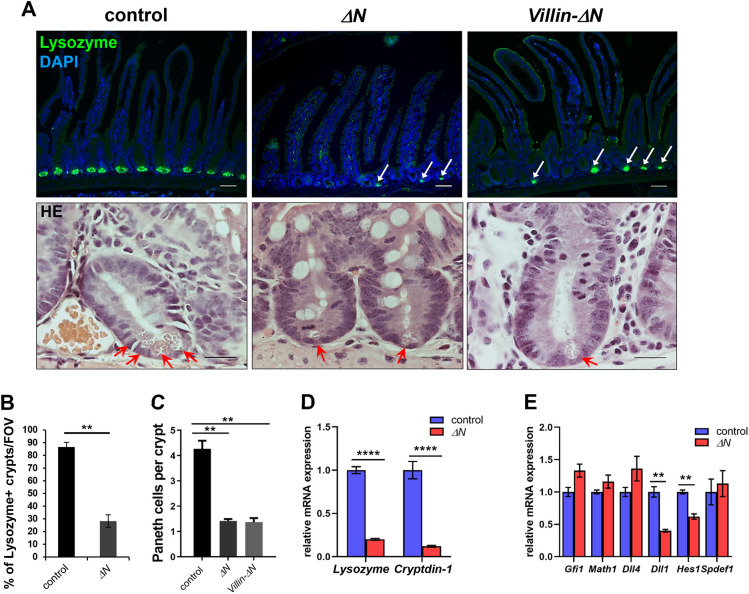
**Strongly reduced numbers of Paneth cells in crypts of Δ*N* mice.** (A) Top: Immunofluorescence using anti-lysozyme antibody staining on sections of PSI of control, Δ*N* and *Villin-*Δ*N* mice (*n*=3 per group). White arrows indicate remaining Paneth cells in Δ*N* and *Villin-*Δ*N* crypts. Scale bars: 20 µm. Nuclear counterstain: DAPI (blue). Bottom: Hematoxylin & Eosin-stained high-magnification images of single crypts to illustrate Paneth cells (red arrows) in PSIs of Δ*N*, *Villin-*Δ*N* and control mice. Scale bars: 50 µm. (B) Quantification of lysozyme-positive crypts per field of view (FOV) in control and Δ*N* mice (*n*=3 per group), *n*≥150 crypts per sample. **P<0.01 (unpaired Welch's *t*-test). (C) Quantification of the average number of Paneth cells per crypt in PSIs of control, Δ*N* or *Villin-*Δ*N* mice (*n*=3 per group). **P<0.01 (unpaired Welch's *t*-test). (D) qRT-PCR for Paneth cell markers lysozyme and cryptdin-1 using RNA from PSIs of Δ*N* and control mice (*n*=6 per group). ****P<0.0001 (multiple *t*-test with Bonferroni correction). (E) qRT-PCR for secretory lineage progenitor markers *Gfi1*, *Math1*, *Dll1* and *Dll4*, as well as for *Hes1* and *Spdef* using RNA from PSI crypts of Δ*N* and control mice (*n*=3 per group). **P<0.01 (multiple *t*-test with Bonferroni correction). Error bars represent s.e.m.

Anti-lysozyme immunofluorescence staining on sections of the SI (PSI, MSI and DSI) showed no more than one or two Paneth cells per crypt in Δ*N* and *Villin-*Δ*N* mice compared with the usual three or four Paneth cells (per visual field) in controls (Fig. 4A; Fig. S3A). Hematoxylin & Eosin staining on PSI sections of Δ*N*, *Villin-*Δ*N* and control mice, as well as *in situ* hybridization using a riboprobe for cryptdin-1 (*Defa1*) or immunofluorescence staining and western blotting using an antibody against the Paneth cell marker Mmp7 confirmed this finding (Fig. 4A; Fig. S3B-D). Loss of Paneth cells was particularly drastic in duodenal crypts (PSI) of Δ*N* and *Villin-*Δ*N* crypts (4.1±0.3 for control, 1.5±0.1 for Δ*N* and 1.4±0.3 cells for *Villin-*Δ*N*), but was somewhat less pronounced in jejunum (MSI) and ileum (DSI) (Fig. 4A; Fig. S3A). The overall reduction in Paneth cells in the absence of NF-κB activity was about 73% compared with controls (Fig. 4B,C).

The loss of Paneth cells in Δ*N* mice was confirmed by significantly diminished mRNA expression of mature Paneth cell markers lysozyme and cryptdin-1 as shown by qRT-PCR of RNA isolated from the SI of Δ*N* and control animals (Fig. 4D). Increased goblet cells and loss of Paneth cells suggest that NF-κB may play a role in cell fate decisions that are made in specialized secretory progenitors, which are thought to be located above the stem cell/Paneth cell zone (for a review, see Tetteh et al., 2015). We thus examined RNA expression of the basic helix-loop-helix (bHLH) transcription factor *Math1* (*Atoh1*, atonal homolog 1), *Gfi1* (growth factor independent 1), the Notch-ligands Delta-like 1 and 4 (*Dll1* and *Dll4*) and the bona fide Notch target *Hes1* (hairy and enhancer-of-split 1), as well as *Spdef* (SAM pointed domain containing ets transcription factor) (Fig. 4E). Whereas *Math1*, *Gfi1*, *Dll4* and *Spdef* expression remained unaltered, the secretory progenitor markers *Dll1* and *Hes1* were significantly reduced in Δ*N* mice compared with controls (Fig. 4E). Our results indicate that the formation of Math1- and Gfi1-positive secretory progenitors is not affected by the absence of NF-κB activity in the intestinal epithelium. This suggests that early secretory precursor cells are generated in the absence of NF-κB activity, but subsequent differentiation into Paneth cells is interrupted in favor of goblet cells.

### NF-κB is required for maturation of Paneth cells

Paneth and goblet cells derive from a common secretory lineage progenitor that, among others, expresses Spdef (Gregorieff et al., 2009; van Es et al., 2012; Buczacki et al., 2013; Basak et al., 2014) (reviewed by Koo and Clevers, 2014). In *Spdef^−/−^* mice, terminal differentiation of Paneth and goblet cells is impaired (Gregorieff et al., 2009; Noah et al., 2010). Surprisingly, expression of Spdef was not altered in Δ*N* mice (Fig. 4E, and see below). Furthermore, *Spdef^−/−^* mice showed normal NF-κB activity in the intestinal epithelium, indicating that both transcription factors are regulated independently (Fig. S4A). Although both Spdef and NF-κB are involved in Paneth cell differentiation, loss of Paneth cells seemed much more dramatic in the absence of NF-κB activity than in *Spdef^−/−^* mice (Fig. 4; for comparison, see Gregorieff et al., 2009).

**Fig. 5.**
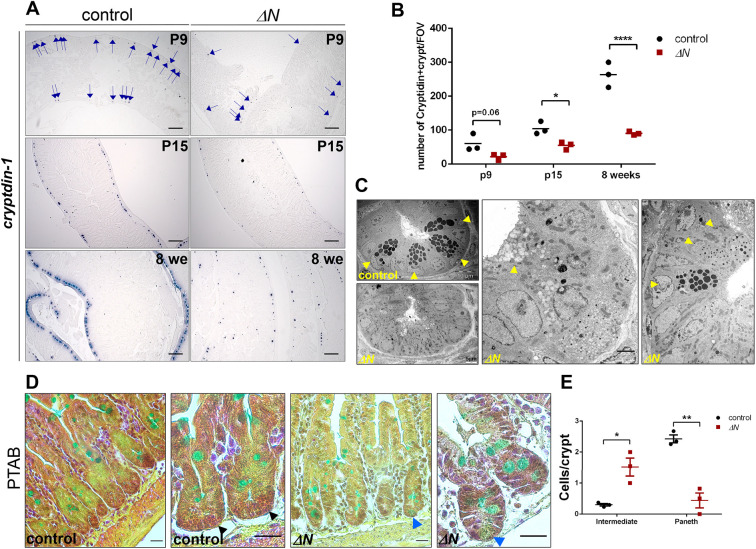
**NF-κB is required for differentiation and maturation of Paneth cells.** (A) ISH on PSI sections of control and Δ*N* mice (*n*=3/group) at P9, P15 and 8 weeks of age using a riboprobe for cryptdin-1. Blue arrows (P9 panels) point to cryptdin-1-positive cells. Scale bars: 200 µm. (B) Quantification of cryptdin-positive crypts in controls and Δ*N* mice at the indicated time points (*n*=3 per group). *P<0.05; ****P<0.0001 (two-way ANOVA). Error bars represent s.e.m. FOV, field of view. (C) TEM analysis of PSI crypts of Δ*N* and control mice (*n*=4 per group). Yellow arrowheads point to mature Paneth cells in control and immature intermediate cells in Δ*N* mice. Bottom left panel shows a Δ*N* crypt lacking Paneth cells. Scale bars: 10 µm (left, control); 5 µm (left, Δ*N*), 2 µm (middle, Δ*N*). (D) PTAB staining on PSI sections of Δ*N* and control mice (*n*=3 per group). Black arrowheads point to mature, lysozyme-containing Paneth cells (red granules) in controls, blue arrowheads to immature intermediate cells (blue-greenish) that lack lysozyme. Scale bars: 20 µm. (E) Quantification of immature intermediate cells versus mature Paneth cells in Δ*N* and control mice (*n*=3 per group). *P<0.05, **P<0.01 (multiple *t*-test with Bonferroni correction). Error bars represent s.e.m.

Differentiated Paneth cells are first observed around 7-10 days after birth (Bry et al., 1994). However, maturation is not completed until 15-17 days after birth (Bry et al., 1994; Clevers and Bevins, 2013). We thus performed comparative studies of Δ*N* and control mice at postnatal day (P) 9 and P15 and at 8 weeks of age (adulthood) (Fig. 5A,B). EGFP expression analysis in *κ-EGFP* reporter mice affirmed that NF-κB was active in crypts at the chosen time points (Fig. S4B). In Δ*N* mice, ISH using a riboprobe for cryptdin-1 revealed a reduced number of cryptdin-1-positive crypts already at P9 prior to final differentiation/maturation, compared with controls (about 50%; Fig. 5A). Upon final differentiation at P15 and in adulthood (8 weeks), the number of cryptdin-1-positive crypts remained strongly decreased in Δ*N* mice compared with controls (Fig. 5A). Quantification of these results and antibody staining for lysozyme at P15 and 8 weeks of age supported this finding (Fig. 5B; Fig. S4C). Of note, not only was the number of crypts containing Paneth cells reduced, but also the number of Paneth cells per crypt (Fig. 4C). Furthermore, whereas in controls the number of cryptdin-1-positive crypts increased about threefold between P15 and 8 weeks, in Δ*N* mice the increase was not statistically significant (Fig. 4B).

To characterize further the observed crypt-based secretory cell types in Δ*N* mice, we visualized crypts by transmission electron microscopy (TEM) comparing SI tissue from Δ*N* and control mice at 8-12 weeks of age (Fig. 5C). TEM images confirmed the lack of mature Paneth cells in most crypts of Δ*N* mice (Fig. S4D). Paneth cells in controls typically showed large dark secretory granules (Fig. 5C). In contrast, in most crypts of Δ*N* mice we observed cells with an altered morphology, containing granules that displayed reduced electron density (Fig. 5C). These cells strongly resembled the immature intermediate cells previously described in a mouse model in which Paneth cells were specifically deleted by cell-lineage ablation (Garabedian et al., 1997), but also in *Spdef^−/−^* mice that lack mature goblet and Paneth cells (Gregorieff et al., 2009; Noah et al., 2010). As we did not detect any mature goblet cells in TEM images of Δ*N*-positive crypts, the aberrant Alcian Blue-positive cells observed in crypts of Δ*N* mice are likely to correspond rather to intermediate cells. For further analysis of these intermediate cells, we also performed phloxine/tartrazine-Alcian Blue (PTAB) co-staining. In mature Paneth cells, phloxine/tartrizine typically stain the granular, lysozyme-containing vacuoles red, as is seen in controls (Fig. 5D). In contrast, Δ*N* crypts only revealed cells with greenish granules that lacked lysozyme (Fig. 5D). Quantification of PTAB staining confirmed that intermediate cells were present only at low levels in Δ*N* mice (Fig. 5E). Together, these data suggest that immature intermediate cells accumulate in crypts of Δ*N* mice and that the few Paneth cells that do form in Δ*N* mice do not appear to mature properly.

Note that numbers of another secretory cell type in the IEC, enteroendocrine cells, were not affected by the absence of NF-κB activity (Fig. S5A). In line with this, mRNA expression of the enteroendocrine cell markers chromogranin A and somatostatin was similar to controls (Fig. S5B).

### Altered expression of Wnt-dependent CBC markers in SIs of Δ*N* mice

NF-κB activity was also observed in a subset of CBCs (Fig. 1). To address whether suppressed NF-κB activity also affects CBCs, the expression of various ISC markers was analyzed. We did not observe any changes in mRNA expression of Wnt-independent CBC markers, such as the pan stem cell marker *Olfm4*, or *Smoc2*, *Lgr1*, *Tert* and *Hopx* (Fig. S5C,D). However, mRNA and protein expression of the Wnt-dependent CBC markers Lgr5, Ascl2, Edn1, Ccnd1 and Tnfrsf19 (also known as Troy) was significantly reduced in crypts of Δ*N* mice compared with controls (Fig. S5E-G). The expression of *Ephb3*, *Msi1* and *Prom1* was unaltered (Fig. S5E), but these latter three factors are not only expressed in CBCs, but also in secretory precursors (Batlle et al., 2002; Kim et al., 2017), which suggests that only Lgr5^+^ CBCs are diminished in the crypts of Δ*N* mice. In contrast to Δ*N* mice, mice with constitutively elevated NF-κB activity in the IECs (*IκBα^IEC-KO^*) showed a significant increase in Wnt activity and Lgr5^+^ CBCs in organoids (Mikuda et al., 2020). Although Ascl2 has also been described as a potential direct NF-κB target gene before (Vlantis et al., 2011), these results indicate that Wnt signaling is diminished and maintenance of Lgr5^+^ CBCs is at least in part dependent on NF-κB activity.

Paneth cells provide niche factors, such as Wnt ligands, but also express the Notch ligand Dll1, and both Notch and Wnt signaling are required for CBC maintenance (Pinto et al., 2003; van Es et al., 2005; Fevr et al., 2007; Farin et al., 2012; for a review, see Gehart and Clevers, 2019). We observed reduced *Hes1* and *Dll1* expression in the crypts of Δ*N* mice (Fig. 4E), indicating reduced Notch signaling in the SI crypts of Δ*N* mice. Finally, the Ki67^+^/Lgr5^+^ cell ratio was significantly higher in Δ*N* mice compared with controls (Fig. S5H), confirming a decrease in Lgr5^+^ cells in the crypts of Δ*N* mice. Together, these results strongly suggest alterations in the cell composition of the SI stem cell niche in Δ*N* mice.

### Wnt3 rescues growth of Δ*N* crypt organoids (mini-guts), but not Paneth/goblet cell fate decisions

**Fig. 6.**
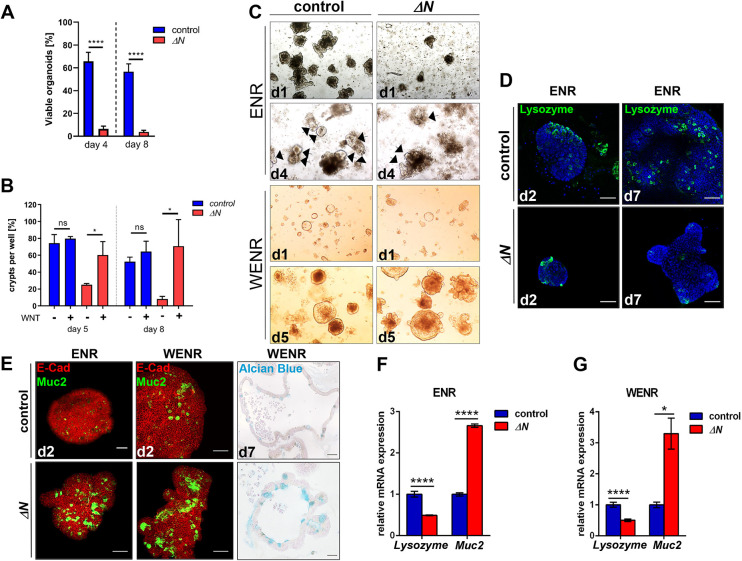
**In crypt organoids, suppression of NF-κB activity results in strongly reduced growth, but also an increase in goblet cells and a loss of Paneth cells.** (A) Quantification of viable organoid formation at days 4 and 8 after isolation of PSI crypts from control or Δ*N* mice (*n*=4 per group). ****P<0.0001 (unpaired *t*-test). Error bars represent s.e.m. (B) Quantification of organoid growth in the presence (WENR) or absence (ENR) of Wnt3 in culture medium at days 5 and 8 after isolation of PSI crypts from control or Δ*N* mice (*n*=3 per group). *P<0.05 (unpaired *t*-test). n.s., not significant. Error bars represent s.d. (C) Upper panels: Representative images of crypt organoids cultured in ENR medium (without Wnt) at day 1 (d1) and day 4 (d4) after isolation of PSI crypts from control and Δ*N* mice (*n*=4 per group). Black arrows indicate viable organoids. Lower panels: Representative images of crypt organoids cultured in WENR medium (plus Wnt) at day 1 (d1) and day 5 (d5) after isolation of PSI crypts from control and Δ*N* mice (*n*=3 per group). (D) Representative immunofluorescence staining using an anti-lysozyme antibody (green) and DAPI (blue) of control and Δ*N* crypt organoids at d2 or d7 in ENR medium. (E) Representative images of immunofluorescence staining using E-cadherin (E-Cad, red) and Muc2 (green) antibodies on control and Δ*N* crypt organoids at d2 in ENR or WENR medium, or of Alcian Blue staining of *Lgr5-EGFP* (control) and *Lgr5-EGFP*;Δ*N* (Δ*N*) organoids, generated from single Lgr5^+^ cells isolated by FACS, at d7 grown in WENR medium. Scale bars: 50 µm. (F,G) qRT-PCR for the Paneth cell marker lysozyme and the goblet cell marker Muc2 using RNA from organoids at d7 in ENR medium (F) or WENR medium (G). *P<0.05, ****P<0.0001 (multiple *t*-test with Bonferroni correction). Error bars represent s.e.m.

To explore further the relevance of NF-κB in Paneth versus goblet cell fate decisions, SI organoid growth and cell composition was examined. Organoids were generated either directly from PSI crypts of Δ*N* and control mice or from single EGFP-positive CBCs obtained from *Lgr5-EGFP;*Δ*N* (Δ*N*) and control mice (*Lgr5-EGFP*; Barker et al., 2007) by fluorescence-activated cell sorting (FACS). In contrast to normal *in vivo* proliferation rates observed in the SI epithelium of Δ*N* mice (Fig. 2A,B), the *ex vivo* ability to culture organoids obtained either from SI crypts of Δ*N* mice or from isolated Lgr5-positive CBCs from *Lgr5-EGFP;*Δ*N* mice was virtually absent in culture medium without Wnt3 (ENR; Fig. 6A,C; Fig. S6A,F): Less than 10% of SI crypts isolated from Δ*N* mice generated organoids after 4-8 days of culture (Fig. 6A), which might be a consequence of Paneth cell ablation in Δ*N* intestinal epithelia. This is in line with previous studies that showed that in the absence of Paneth cells, no organoids are formed (Sato et al., 2011). However, supplementation of organoid culture medium with Wnt3 ligand (WENR) restored Δ*N* organoid growth and Ki67 expression after 5, 7 or 8 days of culture, whereas organoid growth of controls was not affected [Fig. 6B,C; Fig. S6A,B,F (Fig. S6B shows EdU incorporation)]. As expected, expression of the CBC markers *Lgr5* and *Ascl2* was also reduced in Δ*N* organoids grown in ENR medium (Fig. S6C), but addition of Wnt3 caused a significant increase of both markers (Fig. S6D). Thus, the rescued growth of Δ*N* organoids in the presence of Wnt might be due to the re-establishment of a functioning stem cell niche (Fig. 6B,C; Fig. S6A,B).

Similar to observations in SI crypts, NF-κB was predominantly active in Paneth cells of organoids derived from *κ-EGFP* mice (Fig. S6E), and Δ*N* organoids had strongly reduced numbers of Paneth cells and increased numbers of goblet cells compared with controls (Fig. 6D,E). This was verified by qRT-PCR of the Paneth cell marker lysozyme and the goblet cell marker *Muc2* in Δ*N* and control organoids (Fig. 6F). In contrast to growth restoration of Δ*N* organoids, the presence of Wnt3 in the culture medium did not result in any changes of lysozyme or Muc2 expression (Fig. 6E,G). These data indicate that in ΔN organoids Wnt3 alone is not sufficient to re-establish Paneth cell formation and reduce the numbers of goblet cells.

### Wnt3, Wnt10A and Sox9 expression is dependent on NF-κB activity in SI crypts and organoids

**Fig. 7.**
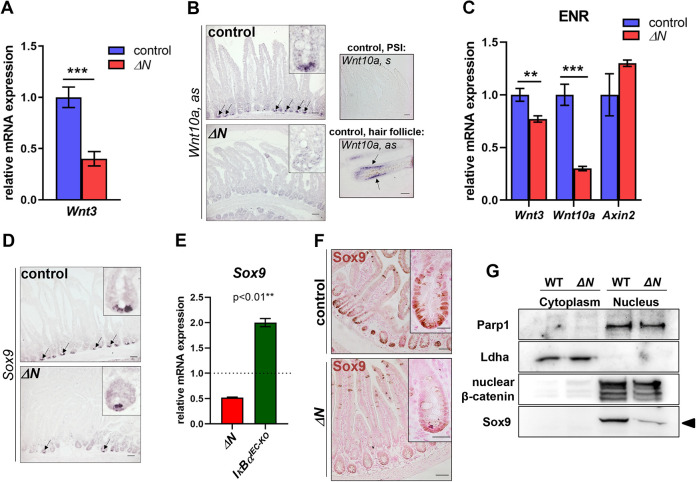
***Wnt3*, *Wnt10a* and *Sox9* expression is significantly reduced or absent in SI crypts and crypt organoids of Δ*N* mice.** (A) qRT-PCR for *Wnt3* using RNA from isolated PSI crypts of Δ*N* and control mice (*n*=3 per group). ***P<0.001 (unpaired Welch's *t*-test). Error bars represent s.e.m. (B) Left: ISH using antisense riboprobes for *Wnt10a* on PSI sections of control and Δ*N* mice (*n*=3 per group). Black arrows point to mRNA expression in crypts. Insets show magnified images of crypts. Right: Sense riboprobe for *Wnt10a* on PSI sections of controls (negative control; top), and *Wnt10a* antisense riboprobe on sagittal skin sections showing *Wnt10a* mRNA expression in the inner root sheath of an anagen hair follicle (black arrows; positive control; bottom). (C) qRT-PCR for *Wnt3*, *Wnt10a* and *Axin2* using RNA from bulk crypt organoids of Δ*N* and control mice (*n*=3 per group) cultured without Wnt (ENR). **P<0.01, ***P<0.001 (multiple *t*-test with Bonferroni correction). Error bars represent s.e.m. (D) ISH using an antisense riboprobe for *Sox9* on PSI sections of control and Δ*N* mice. Black arrows point to mRNA expression in crypts. Insets show magnified images of crypts. (E) qRT-PCR for *Sox9* using RNA from PSIs of Δ*N* and *IκBα^IEC-KO^* mice (*n*=3 per group). Expression level of control mice set to one. **P<0.01 (unpaired Welch's *t*-test). Error bars represent s.e.m. (F) IHC using a Sox9 antibody on PSI sections of control and Δ*N* mice (*n*=3 per group) shows strongly reduced Sox9 expression in Δ*N* mice. Insets show magnified images of crypts. Scale bars: 50 µm (main panels); 20 µm (insets). (G) PSI cytoplasmic and nuclear extracts of control and Δ*N* mice (*n*=3 per group) were used for SDS-PAGE western blotting to confirm the presence of nuclear Sox9 (arrowhead). Lactate dehydrogenase A (Ldha) was used as positive control for cytoplasmic extracts. Parp1 and active nuclear non-phosphorylated β-catenin were used as positive controls for nuclear extracts.

To elucidate the molecular mechanism behind defects in Paneth cell differentiation in Δ*N* mice, we examined Wnt activity and expression of Wnt pathway components that have previously been linked to NF-κB signaling or IEC self-renewal. A Wnt reporter using the *Axin2* promoter (*cond-lacZ*) and analysis of *Axin2* mRNA expression suggested that overall Wnt activity was maintained in the crypts of Δ*N* mice (Fig. S7A,B). This may be due to TA cells migrating into the crypts in the absence of Paneth cells (Fig. 2A,C; Fig. S7A) (Mori-Akiyama et al., 2007; Durand et al., 2012; Kim et al., 2012). However, expression of *Wnt3* mRNA was significantly reduced in isolated crypts of Δ*N* mice compared with controls (Fig. 7A). Wnt3 is considered the main Wnt ligand produced in SI crypts (Gregorieff et al., 2005; Farin et al., 2012). Interestingly, mRNA expression of the Wnt ligand *Wnt10a* was also strongly downregulated or absent in SI crypts and crypt organoids (grown in ENR medium) of Δ*N* mice compared with controls, as shown by ISH or via qRT-PCR (Fig. 7B,C). Similar to an earlier study, our ISH results suggest that Wnt10a is expressed in Paneth cells (Fig. 7B) (Berger et al., 2016). *Wnt3* has not yet been identified as NF-κB target gene, which is why reduced *Wnt3* mRNA expression might be connected with the lack of Paneth cells in the crypts of Δ*N* mice (see above and Fig. 4). However, *Wnt10a* has been identified as downstream target of NF-κB previously (Krappmann et al., 2004; Zhang et al., 2009; Tomann et al., 2016). These results support our findings of impaired Wnt signaling in SI crypts when NF-κB activity is suppressed (see above and Fig. S5).

The transcription factor Sox9 (SRY-box containing gene 9) is required for Paneth cell differentiation (Bastide et al., 2007; Mori-Akiyama et al., 2007) and has been described as a Wnt, but also as a direct NF-κB target gene (Blache et al., 2004; Tomann et al., 2016). mRNA and protein expression of Sox9 was significantly diminished or absent in SI crypts of Δ*N* mice compared with controls (set to 1) or with *IκBα^IEC-KO^* mice (Fig. 7D-G; Fig. S7C). Furthermore, we show that Sox9 is co-expressed with EGFP in *κ-EGFP* reporter mice (Fig. S7D-I). Thus, our data indicate that in Paneth cells and their progenitors Sox9 expression is at least partly regulated by NF-κB, which is why loss of NF-κB activity would result in defective Paneth cell differentiation in secretory progenitors.

## DISCUSSION

Self-renewal of the small intestinal epithelium relies mainly on Lgr5-positive stem cells (CBCs) in the crypts and on gradients of special signals, in particular Wnt and Notch (reviewed by Barker et al., 2008; Clevers, 2013; Gehart and Clevers, 2019). Local concentrations of Wnt and Notch determine the type of secretory cell to be formed or whether an enterocyte is generated. Thus far, it has remained unknown whether NF-κB signaling also plays a role in proper SI self-renewal or lineage specification. Ubiquitous suppression of NF-κB (Δ*N*) did not result in intestinal inflammation concomitant with alterations in IEC proliferation and increased apoptosis, which was observed in IEC-specific IKK/NF-κB KOs (Schwitalla et al., 2013; Vlantis et al., 2016); reviewed by Taniguchi and Karin, 2018). This enabled us to demonstrate for the first time an essential role for NF-κB in Paneth versus goblet cell fate decisions. As this was confirmed in organoids, which are devoid of mesenchymal tissue, we conclude that this is an IEC-intrinsic NF-κB function. Paneth cells are the main producers of Wnt and Notch ligands, which are necessary for crypt homeostasis and *ex vivo* organoid growth (Sato et al., 2009, 2011). *In vivo* absence of Paneth cells is thought to be overcome by Wnt production in the underlying mesenchyme, for instance by subepithelial telocytes (Durand et al., 2012; Farin et al., 2012; Kim et al., 2012; Shoshkes-Carmel et al., 2018), which is why the overall growth of the SI epithelium appeared to be normal in Δ*N* mice (see Fig. 2).

The crypts of Δ*N* mice were mainly filled with highly proliferative TA cells that are derived from Lgr5-positive CBCs and are normally located adjacent to SI crypts. This is expected because it was previously observed in the absence of Paneth cells in *Atoh1* (*Math1*) and *Sox9* KO mice (Mori-Akiyama et al., 2007; Durand et al., 2012; Kim et al., 2012). TA cells comprise progenitors for absorptive and secretory cell lineages (reviewed by Tetteh et al., 2015). Our TEM images showed that, in addition to TA cells, Δ*N*-positive crypts contained aberrant immature intermediate cells, and together these cell types, which are normally not present in crypts, might have resulted in no change to proliferation rates or expression of *Prom1*, *Msi1* and *Ephb3* (Figs 2 and 5; Fig. S5). The significantly reduced Wnt-dependent expression of *Lgr5*, *Ascl2*, *Tnfrsf19* or *Edn1* observed in SI crypts of Δ*N* mice may thus have been caused by decreased Wnt activity together with changes in CBC numbers and/or their differentiation rate. Δ*N* organoids grown in Wnt3-containing culture medium re-express *Lgr5* and *Ascl2* at higher levels than controls, suggesting that Wnt rather than NF-κB is the major regulator of Lgr5 and Ascl2. It also indicates that NF-κB activity is essential for fine-tuning of Wnt signaling in SI crypts. Various studies on inflammation or tumorigenesis in the intestinal epithelium have suggested that local aberrant NF-κB activation enhances Wnt signaling and CBC niche expansion, whereas deletion of NF-κB p65/RelA in IECs led to a delay of this expansion (Schwitalla et al., 2013; Mikuda et al., 2020; Zhao et al., 2020). However, it currently remains unknown whether NF-κB has a direct role in CBC homeostasis. The occasional NF-κB activity observed in what appeared to be CBCs (see Fig. 1) might also correspond to already-primed stem cells about to migrate out of the crypt to become secretory progenitors.

Paneth and goblet cells derive from the same secretory progenitor. Paneth cells are formed in an environment of high Wnt/β-catenin, whereas goblet cell formation requires low Wnt activity (for a review, see Buske et al., 2011). The exact mechanism of this switch remains unknown, but fine-tuning of Wnt signaling might in part be regulated by Shp2/Mek1/MAPK signaling that interferes with Tcf4/β-catenin signaling in goblet/Paneth cell progenitors (Heuberger et al., 2014). According to this study, goblet cell differentiation requires high Shp2/MAPK activity, which inhibits Wnt/β-catenin signaling, whereas Paneth cells are only formed in an environment of low Shp2/MAPK and, hence, high Wnt/β-catenin activity (Heuberger et al., 2014). Increased numbers of goblet cells at the expense of Paneth cells indicate that fine-tuning of Wnt activity is disturbed in Δ*N* mice, in which one would expect high Shp2/MAPK signaling and low Wnt activity. In line with this, expression of *Wnt3* and *Wnt10* mRNA was significantly reduced in SI crypts and in organoids derived from crypts or a single Lgr5-positive CBC. A potential connection between Shp2/MAPK signaling and NF-κB activity in SI crypt homeostasis remains to be evaluated. Although Wnt3 is required for stem cell niche maintenance, the role of Wnt10a is not well defined yet. It was shown previously that Wnt10a is expressed in mature Paneth cells (Berger et al., 2016), suggesting that it might regulate CBC proliferation and maintenance together with Wnt3, but it may additionally control Paneth cell differentiation in the secretory progenitor. We have demonstrated that Wnt3 alone is not sufficient to rescue loss of Paneth cells in Δ*N* organoids, which strongly suggests that NF-κB regulates the expression of additional Wnts required for proper Paneth cell differentiation and maturation. Thus, Paneth cells may direct their own generation. Interestingly, in skin appendages, such as hair follicles or filiform papillae of the tongue, Wnt10a is specifically required for differentiation and morphogenesis in post-mitotic cells that have already been primed (Hammerschmidt and Schlake, 2007; Xu et al., 2017; Krieger et al., 2018).

Loss of Sox9 expression and the consequential lack of Paneth cell differentiation alone does not lead to increased numbers of goblet cells (Bastide et al., 2007; Mori-Akiyama et al., 2007), as observed in Δ*N* mice. Thus, the increased numbers of goblet cells in Δ*N* mice must be due to molecular and/or physiological changes in addition to ablation of Sox9 expression due to suppressed NF-κB activity in secretory progenitors. We observed decreased Dll1 expression in crypts and organoids of Δ*N* mice, which inevitably will lead to reduced local Notch signaling. Notch ligands Dll1 and Dll4 are both expressed on mature Paneth cells, are partly redundant, activate Notch signaling via Notch receptors on adjacent CBCs and are required for stem cell maintenance (Pellegrinet et al., 2011; Sato et al., 2011). However, Dll1 is also highly expressed on secretory progenitors and its expression appears to be associated with proliferation cessation (Stamataki et al., 2011; van Es et al., 2012; for a review, see Tetteh et al., 2015). Furthermore, *Dll1* KOs have increased goblet cell numbers (Pellegrinet et al., 2011; Stamataki et al., 2011). Thus far, *Dll1* has not been identified as a target gene of NF-κB. It therefore may be linked to loss of Paneth cells and/or to an as-yet-unknown effect in secretory progenitors due to suppressed NF-κB activity. Taken together, in Δ*N* mice decreased *Dll1* expression in SI crypts, together with local loss of Wnt3, Wnt10a and Sox9 expression, might tilt the fate decisions of secretory progenitors towards goblet cells.

The upstream activator of NF-κB that specifically regulates Paneth cell differentiation in the SI epithelium has not yet been defined. Particular members of the TNF family appear to activate NF-κB in physiological processes unrelated to inflammation or development of the immune system. In fetal skin appendage development and hair follicle self-renewal, NF-κB is exclusively activated by the TNF family member Eda-A1 (ectodysplasin A1; also known as Eda; Kumar et al., 2001; Schmidt-Ullrich et al., 2001; Laurikkala et al., 2002; Schmidt-Ullrich et al., 2006; Krieger et al., 2018). Eda-A1 signaling does not seem to be responsible for activation of intestinal NF-κB activity, because the SIs of *Eda-*A1 mutant mice (*tabby* mice) were indistinguishable from controls (K.K. and R.S.-U., unpublished data). The search for activators of NF-κB signaling in the SI will thus continue in the future, but Tnfrsf19 is a potential candidate as it is expressed in SI crypts, is regulated by Wnt in Lgr5-positive CBCs and appears to modulate Wnt signaling in human colon carcinoma cell lines (Fafilek et al., 2013). Note that decreased Wnt ligand expression in SI crypts of Δ*N* mice might also have led to reduced Tnfrsf19 expression (see Fig. S5E). A recent publication also showed that the Lgr5-Tnfrsf19 interaction is able to activate NF-κB in colon carcinoma cell lines (Lai et al., 2020). Importantly, the current study is yet another proof that signaling crosstalk between NF-κB and Wnt plays a role not only in tumorigenesis (Schwitalla et al., 2013), but also in physiological processes. A reciprocal requirement of NF-κB and Wnt signaling is essential for fetal hair follicle induction to prevent premature differentiation, but also for subsequent morphogenesis and in adult hair follicle cycling (Fliniaux et al., 2008; Zhang et al., 2009; Kloepper et al., 2014; Tomann et al., 2016; Krieger et al., 2018). In hair follicles, this mainly involves *Wnt10a* and *Wnt10b*, which are target genes of NF-κB, whereas Wnt upregulates Edar (ectodysplain-A receptor) expression, the receptor for Eda-A1 (Zhang et al., 2009). Although Wnt10b is not expressed in IECs (data not shown), our data confirm previous findings of Wnt10a expression in SI crypts (Berger et al., 2016). It will thus be important to examine a role for NF-κB–Wnt10a interactions in SI crypt homeostasis in the future.

## MATERIALS AND METHODS

### Mice

All aspects of animal care and experimental protocols were approved by the Berlin Animal Review Board (LAGeSo Berlin; Reg. G 0077/08, G 0082/13, G 0358/13 and X 9013/11) and all animal studies were performed in accordance with ARRIVE guidelines. Mice were genotyped by PCR of genomic DNA. The following genetically modified lines were used for our study: *B6-Tg(κ-Gal)3Rsu* (*κ-Gal*) (Schmidt-Ullrich et al., 1996; Schmidt-Ullrich et al., 2001), *B6-Tg(κ-EGFP)3Pt/Rsu* (*κ-EGFP*) (Tomann et al., 2016), *129;129P2-ctnnb1^tm(NFKBIAΔN)1RSU^*(Δ*N*) (Schmidt-Ullrich et al., 2001), *129;129P2-ctnnb1^tm(NFKBIAΔNfl/fl)RSU^*(*loxP-*Δ*N*) (Schmidt-Ullrich et al., 2001; Freund et al., 2005; Henke et al., 2007; Krieger et al., 2018), *Tg(Vil-cre)20Syr* (*villin-Cre*) (el Marjou et al., 2004), *B6;129P2-Nfkbia^tm1Kbp^* (Beg et al., 1995; Rupec et al., 2005), *B6;129P2-Nfkbia^tm1Kbp^;Tg(Vil-cre)20Syr* (*IκBα^IEC-KO^*) (Mikuda et al., 2020), *B6.129P2-Lgr5^tm1(cre/ERT2)Cle^* (*Lgr5-EGFP-IRES-creERT2*, here referred to as *Lgr5-EGFP*) (Barker et al., 2007), *B6-Axin2^tm1Mdcb^* (*cond-lacZ*) (Lustig et al., 2002; Yu et al., 2005), *Spdef ^tm1.1Cle^* (Gregorieff et al., 2009). For each experiment three to seven mice per group (line) were used. Mice were sacrificed at 8-12 weeks of age, or P9 and P15 as indicated.

### Organoid cultures

Mouse intestine organoid culture in Matrigel was performed as described previously (Sato and Clevers, 2013). Briefly, duodenal (PSI) crypts were isolated by filtration (70 μm) and centrifugation (300 ***g*** for 5 min) of selected fractions following mechanical dissociation (shaking) of villi and crypts, a 5 min incubation at room temperature with 8 mM EDTA and a 25 min rotation at 4°C with 2 mM EDTA. Approximately five-hundred crypts were embedded in 50 μl Matrigel (BD Biosciences, 356,231) and cultured in Advanced DMEM/F12 medium (12634; Life Technologies), supplemented with N2 and B27 (Life Technologies, 17502-040 and 17504-044, respectively), mNoggin (PeproTech, final concentration 100 ng/ml), R-spondin (R&D Systems, final concentration 500 ng/ml), mEGF (mouse epidermal growth factor, 315-09, PeproTech; final concentration 50 ng/ml) and N-acetylcysteine (5619, Tocris Bioscience; final concentration 500 μM). For single-cell sorting experiments, isolated crypts were incubated in culture medium for 45 min at 37°C followed by trituration with a glass pipette. Dissociated cells were passed through a cell strainer with a pore size of 40 μm. EGFP-positive cells were sorted by flow cytometry (BD FACS ARIA II). Single viable epithelial cells were gated by forward scatter, side scatter and pulse-width parameter, and by negative staining for propidium iodide. Sorted cells were collected in culture medium and embedded in Matrigel containing Jagged-1 peptide (1 μM; AnaSpec) at 100 cells/well (in 48-well plates, 10 μl Matrigel). After sorting the culture medium (250 μl for 48-well plates) was supplemented with Y-27632 (10 μM; Sigma-Aldrich) for 24 h. Thereafter, Y-27632-free medium was used and changed every day. For passage, organoids were removed from Matrigel and mechanically dissociated into single-crypt domains, and then transferred to new Matrigel.

### Immunofluorescence, immunohistochemistry, ISH, TUNEL assay, BrdU and EdU incorporation

Unless indicated otherwise, histological and biochemical analyses were performed on sections of the duodenum (PSI) of 8- to 12-week-old mice. Cleansed intestinal pieces were coiled prior to treatment (‘swiss role’; Moolenbeek and Ruitenberg, 1981). For ISH, immunohistochemistry (IHC) or immunofluorescence, the tissue was fixed in Bouin's fixative (for ISH) or in 4% paraformaldehyde/MEM overnight at 4°C, followed by dehydration and paraffin-embedding. Immunofluorescence and ISH on paraffin sections were performed as described previously (Gregorieff et al., 2005; Zhang et al., 2009; Heuberger et al., 2014). For IHC, a 3,3′-diaminobenzidine (DAB) kit (882014 2-Solution DAB Kit, Invitrogen) was used for antibody detection. Alcian Blue, Hematoxylin & Eosin, or nuclear DAPI (blue) or Fast Red staining were carried out according to standard protocols. TUNEL staining was performed on 5-µm-thick sections of PSIs using the In Situ Cell Death Detection kit (11684795910, Roche) according to the manufacturer's protocol. For proliferation studies, BrdU in 0.9% NaCl was injected intraperitoneally (100 µg/g body weight). PSI samples were taken after 4 or 24 h, dehydrated and embedded in paraffin. Visualization was performed using the M.O.M. Immunodetection Kit (PK-2200, Vector Laboratories). EdU incorporation and detection was performed using the Click-iT EdU Cell Proliferation Kit for Imaging and Alexa Fluor 555 dye (C10338, Invitrogen) as described in the manufacturer's protocol.

Images were collected using a ZEISS LSM800 or Leica SP5-7-8 and processed using ImageJ.

Antibodies and ISH cDNA probes are provided in supplementary Materials and Methods. Images were obtained using a conventional (Leica SP5-7-8) or confocal (Zeiss LSM800) Zeiss microscope.

X-Gal staining for detection of β-galactosidase activity was as described previously (Schmidt-Ullrich et al., 1996, 2001). Tissue was stained as whole-mount, dehydrated in an ethanol series (30-100%) and embedded in Technovit 7100 plastic (Heraeus Kulzer). Sections of 5-8 μm thickness were counterstained with 0.1% Pyronin G (45005, Sigma-Aldrich).

PTAB staining was performed on 5-μm-thick sections for scoring of PT+AB– (Paneth cells), PT–AB+ (goblet cells) and PT+AB+ (intermediate cells) cell numbers, as described previously (Dekaney et al., 2019).

### TEM

Dissected pieces of PSI tissue of 2-3 mm^3^ from 8- to 12-week-old mice were fixed by immersion in 4% (w/v) paraformaldehyde and 2.5% (v/v) glutaraldehyde in 0.1 M phosphate buffer for 2 h at room temperature. Samples were post-fixed with 1% (v/v) osmium tetroxide for 3 h at room temperature, dehydrated in a graded series of ethanol, and embedded in PolyBed 812 resin (Polysciences). Ultrathin sections (60-80 nm) were stained with uranyl acetate and lead citrate, and examined at 80 kV with a Zeiss EM 910 electron microscope. Images were acquired with a Quemesa CCD camera using iTEM software (EMSIS).

### Quantification of Paneth cells and goblet cells

Lysozyme protein specifically expressed in secretory granules of Paneth cells was used as a surrogate marker to identify Paneth cells in the SI. Paraffin sections (5 μm) were stained with an anti-lysozyme antibody and a conventional microscope was used to take pictures at 100× magnification of random, non-overlapping SI areas within a section. The cell-counting plug-in of ImageJ was used to count lysozyme-positive and -negative crypts, and the percentage of lysozyme-positive crypts out of total number of crypts was calculated. A minimum of 150 crypts was counted for three to five biological replicates [Δ*N* (suppressed NF-κB activity) and control mice]. For quantification of goblet cells, PSI (proximal small intestine, duodenum) sections were stained with Alcian Blue. Conventional microscope analysis was performed at 600× magnification by counting Alcian Blue-positive cells per crypt for 50 crypts for three biological replicates (Δ*N*, *villin-*Δ*N* and control mice). Unpaired Student’s *t*-test with Welch's correction was used to calculate *P*-values. *P*≤0.05 was considered significant.

### Quantitative RT-PCR

For quantitative real-time PCR (qRT-PCR), cDNA was generated from total RNA using the iScript cDNA Synthesis Kit (Bio-Rad Laboratories). Primers are provided in supplementary Materials and Methods. Data analysis was performed with CFX96 software (Bio-Rad Laboratories), which is based on the ΔΔCT method. All target genes were standardized to reference genes *Gapdh* and *Hprt* (M-value <0.5 for homogeneous samples). Outliers were defined by the Grubbs test using GraphPad Prism 8. All qRT-PCR results were treated according to MIQE guidelines (Bustin et al., 2009). All control values were normalized to one.

### Protein extractions and western blotting

A freshly isolated piece of small intestine was shock-frozen in liquid nitrogen, pulverized using a mortar and incubated in 500 µl RIPA buffer for 4 h on a rotating table. The sample was centrifuged for 30 min at 14,000 rpm (21,952 ***g***). Cytoplasmic and nuclear extracts were performed after crypt isolation from the small intestine. The supernatant was used for western blotting in SDS-PAGE and protein concentration determined using the Bradford method as reported elsewhere (Mikuda et al., 2018).

### Statistical analyses

Statistical analyses were performed using GraphPad Prism 8. Unless stated otherwise, significance was estimated using either unpaired Student's *t*-test with Welch's correction or multiple *t*-test with Bonferroni correction and two-way ANOVA. *P*-values less than 0.05 were defined as significant. Mean and s.e.m. are reported in the figure legends. FACS data were analyzed using FlowJo_v10, and ImageJ was used for protein and QuPath for cell quantification.

## Supplementary Material

Supplementary information

Reviewer comments

## References

[DEV199683C1] Barker, N., Bartfeld, S. and Clevers, H. (2010). Tissue-resident adult stem cell populations of rapidly self-renewing organs. *Cell Stem Cell* 7, 656-670. 10.1016/j.stem.2010.11.016 21112561

[DEV199683C2] Barker, N., van de Wetering, M. and Clevers, H. (2008). The intestinal stem cell. *Genes Dev.* 22, 1856-1864. 10.1101/gad.1674008 18628392 PMC2735277

[DEV199683C3] Barker, N., van Es, J. H., Kuipers, J., Kujala, P., van den Born, M., Cozijnsen, M., Haegebarth, A., Korving, J., Begthel, H., Peters, P. J. et al. (2007). Identification of stem cells in small intestine and colon by marker gene Lgr5. *Nature* 449, 1003-1007. 10.1038/nature06196 17934449

[DEV199683C4] Basak, O., van de Born, M., Korving, J., Beumer, J., van der Elst, S., van Es, J. H. and Clevers, H. (2014). Mapping early fate determination in Lgr5+ crypt stem cells using a novel Ki67-RFP allele. *EMBO J.* 33, 2057-2068. 10.15252/embj.201488017 25092767 PMC4195772

[DEV199683C5] Bastide, P., Darido, C., Pannequin, J., Kist, R., Robine, S., Marty-Double, C., Bibeau, F., Scherer, G., Joubert, D., Hollande, F. et al. (2007). Sox9 regulates cell proliferation and is required for Paneth cell differentiation in the intestinal epithelium. *J. Cell Biol.* 178, 635-648. 10.1083/jcb.200704152 17698607 PMC2064470

[DEV199683C6] Batlle, E., Henderson, J. T., Beghtel, H., van den Born, M. M., Sancho, E., Huls, G., Meeldijk, J., Robertson, J., van de Wetering, M., Pawson, T. et al. (2002). Beta-catenin and TCF mediate cell positioning in the intestinal epithelium by controlling the expression of EphB/ephrinB. *Cell* 111, 251-263. 10.1016/S0092-8674(02)01015-2 12408869

[DEV199683C7] Beg, A. A., Sha, W. C., Bronson, R. T. and Baltimore, D. (1995). Constitutive NF-kappa B activation, enhanced granulopoiesis, and neonatal lethality in I kappa B alpha-deficient mice. *Genes Dev.* 9, 2736-2746. 10.1101/gad.9.22.2736 7590249

[DEV199683C8] Ben-Neriah, Y. and Karin, M. (2011). Inflammation meets cancer, with NF-kappaB as the matchmaker. *Nat. Immunol.* 12, 715-723. 10.1038/ni.2060 21772280

[DEV199683C9] Berger, E., Rath, E., Yuan, D., Waldschmitt, N., Khaloian, S., Allgauer, M., Staszewski, O., Lobner, E. M., Schottl, T., Giesbertz, P. et al. (2016). Mitochondrial function controls intestinal epithelial stemness and proliferation. *Nat. Commun.* 7, 13171. 10.1038/ncomms13171 27786175 PMC5080445

[DEV199683C10] Blache, P., van de Wetering, M., Duluc, I., Domon, C., Berta, P., Freund, J. N., Clevers, H. and Jay, P. (2004). SOX9 is an intestine crypt transcription factor, is regulated by the Wnt pathway, and represses the CDX2 and MUC2 genes. *J. Cell Biol.* 166, 37-47. 10.1083/jcb.200311021 15240568 PMC2172132

[DEV199683C11] Blanpain, C. and Fuchs, E. (2014). Stem cell plasticity. Plasticity of epithelial stem cells in tissue regeneration. *Science* 344, 1242281. 10.1126/science.1242281 24926024 PMC4523269

[DEV199683C12] Bry, L., Falk, P., Huttner, K., Ouellette, A., Midtvedt, T. and Gordon, J. I. (1994). Paneth cell differentiation in the developing intestine of normal and transgenic mice. *Proc. Natl. Acad. Sci. USA* 91, 10335-10339. 10.1073/pnas.91.22.10335 7937951 PMC45014

[DEV199683C13] Buczacki, S. J., Zecchini, H. I., Nicholson, A. M., Russell, R., Vermeulen, L., Kemp, R. and Winton, D. J. (2013). Intestinal label-retaining cells are secretory precursors expressing Lgr5. *Nature* 495, 65-69. 10.1038/nature11965 23446353

[DEV199683C14] Buske, P., Galle, J., Barker, N., Aust, G., Clevers, H. and Loeffler, M. (2011). A comprehensive model of the spatio-temporal stem cell and tissue organisation in the intestinal crypt. *PLoS Comput. Biol.* 7, e1001045. 10.1371/journal.pcbi.1001045 21253562 PMC3017108

[DEV199683C15] Bustin, S. A., Benes, V., Garson, J. A., Hellemans, J., Huggett, J., Kubista, M., Mueller, R., Nolan, T., Pfaffl, M. W., Shipley, G. L. et al. (2009). The MIQE guidelines: minimum information for publication of quantitative real-time PCR experiments. *Clin. Chem.* 55, 611-622. 10.1373/clinchem.2008.112797 19246619

[DEV199683C16] Carulli, A. J., Samuelson, L. C. and Schnell, S. (2014). Unraveling intestinal stem cell behavior with models of crypt dynamics. *Integr. Biol. (Camb)* 6, 243-257. 10.1039/c3ib40163d 24480852 PMC4007491

[DEV199683C17] Chen, L. W., Egan, L., Li, Z. W., Greten, F. R., Kagnoff, M. F. and Karin, M. (2003). The two faces of IKK and NF-kappaB inhibition: prevention of systemic inflammation but increased local injury following intestinal ischemia-reperfusion. *Nat. Med.* 9, 575-581. 10.1038/nm849 12692538

[DEV199683C18] Clevers, H. (2013). The intestinal crypt, a prototype stem cell compartment. *Cell* 154, 274-284. 10.1016/j.cell.2013.07.004 23870119

[DEV199683C19] Clevers, H. C. and Bevins, C. L. (2013). Paneth cells: maestros of the small intestinal crypts. *Annu. Rev. Physiol.* 75, 289-311. 10.1146/annurev-physiol-030212-183744 23398152

[DEV199683C20] Dekaney, C. M., King, S., Sheahan, B. and Cortes, J. E. (2019). Mist1 expression is required for paneth cell maturation. *Cell Mol. Gastroenterol. Hepatol.* 8, 549-560. 10.1016/j.jcmgh.2019.07.003 31330316 PMC6889789

[DEV199683C21] Durand, A., Donahue, B., Peignon, G., Letourneur, F., Cagnard, N., Slomianny, C., Perret, C., Shroyer, N. F. and Romagnolo, B. (2012). Functional intestinal stem cells after Paneth cell ablation induced by the loss of transcription factor Math1 (Atoh1). *Proc. Natl. Acad. Sci. U.S.A.* 109, 8965-8970. 10.1073/pnas.1201652109 22586121 PMC3384132

[DEV199683C22] el Marjou, F., Janssen, K. P., Chang, B. H., Li, M., Hindie, V., Chan, L., Louvard, D., Chambon, P., Metzger, D. and Robine, S. (2004). Tissue-specific and inducible Cre-mediated recombination in the gut epithelium. *Genesis* 39, 186-193. 10.1002/gene.20042 15282745

[DEV199683C23] Fafilek, B., Krausova, M., Vojtechova, M., Pospichalova, V., Tumova, L., Sloncova, E., Huranova, M., Stancikova, J., Hlavata, A., Svec, J. et al. (2013). Troy, a tumor necrosis factor receptor family member, interacts with lgr5 to inhibit wnt signaling in intestinal stem cells. *Gastroenterology* 144, 381-391. 10.1053/j.gastro.2012.10.048 23142137

[DEV199683C24] Farin, H. F., van Es, J. H. and Clevers, H. (2012). Redundant sources of wnt regulate intestinal stem cells and promote formation of paneth cells. *Gastroenterology* 143, 1518-1529.e1517. 10.1053/j.gastro.2012.08.031 22922422

[DEV199683C25] Fevr, T., Robine, S., Louvard, D. and Huelsken, J. (2007). Wnt/beta-catenin is essential for intestinal homeostasis and maintenance of intestinal stem cells. *Mol. Cell. Biol.* 27, 7551-7559. 10.1128/MCB.01034-07 17785439 PMC2169070

[DEV199683C26] Fliniaux, I., Mikkola, M. L., Lefebvre, S. and Thesleff, I. (2008). Identification of dkk4 as a target of Eda-A1/Edar pathway reveals an unexpected role of ectodysplasin as inhibitor of Wnt signalling in ectodermal placodes. *Dev. Biol.* 320, 60-71. 10.1016/j.ydbio.2008.04.023 18508042

[DEV199683C27] Freund, C., Schmidt-Ullrich, R., Baurand, A., Dunger, S., Schneider, W., Loser, P., El-Jamali, A., Dietz, R., Scheidereit, C. and Bergmann, M. W. (2005). Requirement of nuclear factor-kappaB in angiotensin II- and isoproterenol-induced cardiac hypertrophy in vivo. *Circulation* 111, 2319-2325. 10.1161/01.CIR.0000164237.58200.5A 15870116

[DEV199683C28] Fuchs, E. (2007). Scratching the surface of skin development. *Nature* 445, 834-842. 10.1038/nature05659 17314969 PMC2405926

[DEV199683C29] Garabedian, E. M., Roberts, L. J., McNevin, M. S. and Gordon, J. I. (1997). Examining the role of Paneth cells in the small intestine by lineage ablation in transgenic mice. *J. Biol. Chem.* 272, 23729-23740. 10.1074/jbc.272.38.23729 9295317

[DEV199683C30] Garg, P., Ravi, A., Patel, N. R., Roman, J., Gewirtz, A. T., Merlin, D. and Sitaraman, S. V. (2007). Matrix metalloproteinase-9 regulates MUC-2 expression through its effect on goblet cell differentiation. *Gastroenterology* 132, 1877-1889. 10.1053/j.gastro.2007.02.048 17484881

[DEV199683C31] Gehart, H. and Clevers, H. (2019). Tales from the crypt: new insights into intestinal stem cells. *Nat. Rev. Gastroenterol. Hepatol.* 16, 19-34. 10.1038/s41575-018-0081-y 30429586

[DEV199683C32] Ghaleb, A. M., McConnell, B. B., Kaestner, K. H. and Yang, V. W. (2011). Altered intestinal epithelial homeostasis in mice with intestine-specific deletion of the Kruppel-like factor 4 gene. *Dev. Biol.* 349, 310-320. 10.1016/j.ydbio.2010.11.001 21070761 PMC3022386

[DEV199683C33] Gregorieff, A., Pinto, D., Begthel, H., Destree, O., Kielman, M. and Clevers, H. (2005). Expression pattern of Wnt signaling components in the adult intestine. *Gastroenterology* 129, 626-638. 10.1016/j.gastro.2005.06.007 16083717

[DEV199683C34] Gregorieff, A., Stange, D. E., Kujala, P., Begthel, H., van den Born, M., Korving, J., Peters, P. J. and Clevers, H. (2009). The ets-domain transcription factor Spdef promotes maturation of goblet and paneth cells in the intestinal epithelium. *Gastroenterology* 137, 1333-1345.e1-3. 10.1053/j.gastro.2009.06.044 19549527

[DEV199683C35] Greten, F. R., Eckmann, L., Greten, T. F., Park, J. M., Li, Z. W., Egan, L. J., Kagnoff, M. F. and Karin, M. (2004). IKKbeta links inflammation and tumorigenesis in a mouse model of colitis-associated cancer. *Cell* 118, 285-296. 10.1016/j.cell.2004.07.013 15294155

[DEV199683C36] Grinberg-Bleyer, Y., Dainichi, T., Oh, H., Heise, N., Klein, U., Schmid, R. M., Hayden, M. S. and Ghosh, S. (2015). Cutting edge: NF-kappaB p65 and c-Rel control epidermal development and immune homeostasis in the skin. *J. Immunol.* 194, 2472-2476. 10.4049/jimmunol.1402608 25681334 PMC4355158

[DEV199683C37] Guma, M., Stepniak, D., Shaked, H., Spehlmann, M. E., Shenouda, S., Cheroutre, H., Vicente-Suarez, I., Eckmann, L., Kagnoff, M. F. and Karin, M. (2011). Constitutive intestinal NF-kappaB does not trigger destructive inflammation unless accompanied by MAPK activation. *J. Exp. Med.* 208, 1889-1900. 10.1084/jem.20110242 21825016 PMC3171091

[DEV199683C38] Hammerschmidt, B. and Schlake, T. (2007). Localization of Shh expression by Wnt and Eda affects axial polarity and shape of hairs. *Dev. Biol.* 305, 246-261. 10.1016/j.ydbio.2007.02.010 17376426

[DEV199683C39] Henke, N., Schmidt-Ullrich, R., Dechend, R., Park, J. K., Qadri, F., Wellner, M., Obst, M., Gross, V., Dietz, R., Luft, F. C. et al. (2007). Vascular endothelial cell-specific NF-kappaB suppression attenuates hypertension-induced renal damage. *Circ. Res.* 101, 268-276. 10.1161/CIRCRESAHA.107.150474 17585070

[DEV199683C40] Heuberger, J., Kosel, F., Qi, J., Grossmann, K. S., Rajewsky, K. and Birchmeier, W. (2014). Shp2/MAPK signaling controls goblet/paneth cell fate decisions in the intestine. *Proc. Natl. Acad. Sci. USA* 111, 3472-3477. 10.1073/pnas.1309342111 24550486 PMC3948231

[DEV199683C41] Hsu, Y. C., Li, L. and Fuchs, E. (2014). Emerging interactions between skin stem cells and their niches. *Nat. Med.* 20, 847-856. 10.1038/nm.3643 25100530 PMC4358898

[DEV199683C42] Katz, J. P., Perreault, N., Goldstein, B. G., Lee, C. S., Labosky, P. A., Yang, V. W. and Kaestner, K. H. (2002). The zinc-finger transcription factor Klf4 is required for terminal differentiation of goblet cells in the colon. *Development* 129, 2619-2628. 10.1242/dev.129.11.2619 12015290 PMC2225535

[DEV199683C43] Kim, H. S., Lee, C., Kim, W. H., Maeng, Y. H. and Jang, B. G. (2017). Expression profile of intestinal stem cell markers in colitis-associated carcinogenesis. *Sci. Rep.* 7, 6533. 10.1038/s41598-017-06900-x 28747693 PMC5529509

[DEV199683C44] Kim, T. H., Escudero, S. and Shivdasani, R. A. (2012). Intact function of Lgr5 receptor-expressing intestinal stem cells in the absence of Paneth cells. *Proc. Natl. Acad. Sci. USA* 109, 3932-3937. 10.1073/pnas.1113890109 22355124 PMC3309789

[DEV199683C45] Kloepper, J. E., Ernst, N., Krieger, K., Bodo, E., Biro, T., Haslam, I. S., Schmidt-Ullrich, R. and Paus, R. (2014). NF-kappaB activity is required for anagen maintenance in human hair follicles In Vitro. *J. Invest. Dermatol.* 134, 2036-2038. 10.1038/jid.2014.82 24518172

[DEV199683C46] Kolesnichenko, M., Mikuda, N., Hopken, U. E., Kargel, E., Uyar, B., Tufan, A. B., Milanovic, M., Sun, W., Krahn, I., Schleich, K. et al. (2021). Transcriptional repression of NFKBIA triggers constitutive IKK- and proteasome-independent p65/RelA activation in senescence. *EMBO J.* 40, e104296. 10.15252/embj.2019104296 33459422 PMC7957429

[DEV199683C47] Koo, B. K. and Clevers, H. (2014). Stem cells marked by the R-spondin receptor LGR5. *Gastroenterology* 147, 289-302. 10.1053/j.gastro.2014.05.007 24859206

[DEV199683C48] Krappmann, D., Wegener, E., Sunami, Y., Esen, M., Thiel, A., Mordmuller, B. and Scheidereit, C. (2004). The IkappaB kinase complex and NF-kappaB act as master regulators of lipopolysaccharide-induced gene expression and control subordinate activation of AP-1. *Mol. Cell. Biol.* 24, 6488-6500. 10.1128/MCB.24.14.6488-6500.2004 15226448 PMC434242

[DEV199683C49] Krieger, K., Millar, S. E., Mikuda, N., Krahn, I., Kloepper, J. E., Bertolini, M., Scheidereit, C., Paus, R. and Schmidt-Ullrich, R. (2018). NF-kappaB participates in mouse hair cycle control and plays distinct roles in the various pelage hair follicle types. *J. Invest. Dermatol.* 138, 256-264. 10.1016/j.jid.2017.08.042 28942365

[DEV199683C50] Kumar, A., Eby, M. T., Sinha, S., Jasmin, A. and Chaudhary, P. M. (2001). The ectodermal dysplasia receptor activates the nuclear factor-kappaB, JNK, and cell death pathways and binds to ectodysplasin A. *J. Biol. Chem.* 276, 2668-2677. 10.1074/jbc.M008356200 11035039

[DEV199683C51] Lai, S., Cheng, R., Gao, D., Chen, Y. G. and Deng, C. (2020). LGR5 constitutively activates NF-kappaB signaling to regulate the growth of intestinal crypts. *FASEB J.* 34, 15605-15620. 10.1096/fj.202001329R 33001511

[DEV199683C52] Laurikkala, J., Pispa, J., Jung, H. S., Nieminen, P., Mikkola, M., Wang, X., Saarialho-Kere, U., Galceran, J., Grosschedl, R. and Thesleff, I. (2002). Regulation of hair follicle development by the TNF signal ectodysplasin and its receptor Edar. *Development* 129, 2541-2553. 10.1242/dev.129.10.2541 11973284

[DEV199683C53] Le Bail, O., Schmidt-Ullrich, R. and Israel, A. (1993). Promoter analysis of the gene encoding the I kappa B-alpha/MAD3 inhibitor of NF-kappa B: positive regulation by members of the rel/NF-kappa B family. *EMBO J.* 12, 5043-5049. 10.1002/j.1460-2075.1993.tb06197.x 8262046 PMC413764

[DEV199683C54] Lustig, B., Jerchow, B., Sachs, M., Weiler, S., Pietsch, T., Karsten, U., van de Wetering, M., Clevers, H., Schlag, P. M., Birchmeier, W. et al. (2002). Negative feedback loop of Wnt signaling through upregulation of conductin/axin2 in colorectal and liver tumors. *Mol. Cell. Biol.* 22, 1184-1193. 10.1128/MCB.22.4.1184-1193.2002 11809809 PMC134640

[DEV199683C55] Mikuda, N., Kolesnichenko, M., Beaudette, P., Popp, O., Uyar, B., Sun, W., Tufan, A. B., Perder, B., Akalin, A., Chen, W. et al. (2018). The IkappaB kinase complex is a regulator of mRNA stability. *EMBO J.* 37, e98658. 10.15252/embj.201798658 30467221 PMC6293339

[DEV199683C56] Mikuda, N., Schmidt-Ullrich, R., Kargel, E., Golusda, L., Wolf, J., Hopken, U. E., Scheidereit, C., Kuhl, A. A. and Kolesnichenko, M. (2020). Deficiency in IkappaBalpha in the intestinal epithelium leads to spontaneous inflammation and mediates apoptosis in the gut. *J. Pathol.* 251, 160-174. 10.1002/path.5437 32222043

[DEV199683C57] Moolenbeek, C. and Ruitenberg, E. J. (1981). The “Swiss roll”: a simple technique for histological studies of the rodent intestine. *Lab. Anim.* 15, 57-59. 10.1258/002367781780958577 7022018

[DEV199683C58] Mori-Akiyama, Y., van den Born, M., van Es, J. H., Hamilton, S. R., Adams, H. P., Zhang, J., Clevers, H. and de Crombrugghe, B. (2007). SOX9 is required for the differentiation of paneth cells in the intestinal epithelium. *Gastroenterology* 133, 539-546. 10.1053/j.gastro.2007.05.020 17681175

[DEV199683C59] Nenci, A., Becker, C., Wullaert, A., Gareus, R., van Loo, G., Danese, S., Huth, M., Nikolaev, A., Neufert, C., Madison, B. et al. (2007). Epithelial NEMO links innate immunity to chronic intestinal inflammation. *Nature* 446, 557-561. 10.1038/nature05698 17361131

[DEV199683C60] Noah, T. K., Kazanjian, A., Whitsett, J. and Shroyer, N. F. (2010). SAM pointed domain ETS factor (SPDEF) regulates terminal differentiation and maturation of intestinal goblet cells. *Exp. Cell Res.* 316, 452-465. 10.1016/j.yexcr.2009.09.020 19786015 PMC3004755

[DEV199683C61] Pasparakis, M. (2008). IKK/NF-kappaB signaling in intestinal epithelial cells controls immune homeostasis in the gut. *Mucosal. Immunol.* 1 (Suppl 1), S54-S57. 10.1038/mi.2008.53 19079232

[DEV199683C62] Pasparakis, M. (2009). Regulation of tissue homeostasis by NF-kappaB signalling: implications for inflammatory diseases. *Nat. Rev. Immunol.* 9, 778-788. 10.1038/nri2655 19855404

[DEV199683C63] Pasparakis, M., Courtois, G., Hafner, M., Schmidt-Supprian, M., Nenci, A., Toksoy, A., Krampert, M., Goebeler, M., Gillitzer, R., Israel, A. et al. (2002). TNF-mediated inflammatory skin disease in mice with epidermis-specific deletion of IKK2. *Nature* 417, 861-866. 10.1038/nature00820 12075355

[DEV199683C64] Pellegrinet, L., Rodilla, V., Liu, Z., Chen, S., Koch, U., Espinosa, L., Kaestner, K. H., Kopan, R., Lewis, J. and Radtke, F. (2011). Dll1- and dll4-mediated notch signaling are required for homeostasis of intestinal stem cells. *Gastroenterology* 140, 1230-1240.e1-7. 10.1053/j.gastro.2011.01.005 21238454 PMC3066401

[DEV199683C65] Pinto, D., Gregorieff, A., Begthel, H. and Clevers, H. (2003). Canonical Wnt signals are essential for homeostasis of the intestinal epithelium. *Genes Dev.* 17, 1709-1713. 10.1101/gad.267103 12865297 PMC196179

[DEV199683C66] Rupec, R. A., Jundt, F., Rebholz, B., Eckelt, B., Weindl, G., Herzinger, T., Flaig, M. J., Moosmann, S., Plewig, G., Dorken, B. et al. (2005). Stroma-mediated dysregulation of myelopoiesis in mice lacking I kappa B alpha. *Immunity* 22, 479-491. 10.1016/j.immuni.2005.02.009 15845452

[DEV199683C67] Sato, T. and Clevers, H. (2013). Primary mouse small intestinal epithelial cell cultures. *Methods Mol. Biol.* 945, 319-328. 10.1007/978-1-62703-125-7_19 23097115

[DEV199683C68] Sato, T., Vries, R. G., Snippert, H. J., van de Wetering, M., Barker, N., Stange, D. E., van Es, J. H., Abo, A., Kujala, P., Peters, P. J. et al. (2009). Single Lgr5 stem cells build crypt-villus structures in vitro without a mesenchymal niche. *Nature* 459, 262-265. 10.1038/nature07935 19329995

[DEV199683C69] Sato, T., van Es, J. H., Snippert, H. J., Stange, D. E., Vries, R. G., van den Born, M., Barker, N., Shroyer, N. F., van de Wetering, M. and Clevers, H. (2011). Paneth cells constitute the niche for Lgr5 stem cells in intestinal crypts. *Nature* 469, 415-418. 10.1038/nature09637 21113151 PMC3547360

[DEV199683C70] Schmidt-Ullrich, R., Memet, S., Lilienbaum, A., Feuillard, J., Raphael, M. and Israel, A. (1996). NF-kappaB activity in transgenic mice: developmental regulation and tissue specificity. *Development* 122, 2117-2128. 10.1242/dev.122.7.2117 8681793

[DEV199683C71] Schmidt-Ullrich, R., Aebischer, T., Hulsken, J., Birchmeier, W., Klemm, U. and Scheidereit, C. (2001). Requirement of NF-kappaB/Rel for the development of hair follicles and other epidermal appendices. *Development* 128, 3843-3853. 10.1242/dev.128.19.3843 11585809

[DEV199683C72] Schmidt-Ullrich, R., Tobin, D. J., Lenhard, D., Schneider, P., Paus, R. and Scheidereit, C. (2006). NF-kappaB transmits Eda A1/EdaR signalling to activate Shh and cyclin D1 expression, and controls post-initiation hair placode down growth. *Development* 133, 1045-1057. 10.1242/dev.02278 16481354

[DEV199683C73] Schwitalla, S., Fingerle, A. A., Cammareri, P., Nebelsiek, T., Goktuna, S. I., Ziegler, P. K., Canli, O., Heijmans, J., Huels, D. J., Moreaux, G. et al. (2013). Intestinal tumorigenesis initiated by dedifferentiation and acquisition of stem-cell-like properties. *Cell* 152, 25-38. 10.1016/j.cell.2012.12.012 23273993

[DEV199683C74] Shaked, H., Hofseth, L. J., Chumanevich, A., Chumanevich, A. A., Wang, J., Wang, Y., Taniguchi, K., Guma, M., Shenouda, S., Clevers, H. et al. (2012). Chronic epithelial NF-kappaB activation accelerates APC loss and intestinal tumor initiation through iNOS up-regulation. *Proc. Natl. Acad. Sci. USA* 109, 14007-14012. 10.1073/pnas.1211509109 22893683 PMC3435160

[DEV199683C75] Shoshkes-Carmel, M., Wang, Y. J., Wangensteen, K. J., Toth, B., Kondo, A., Massasa, E. E., Itzkovitz, S. and Kaestner, K. H. (2018). Subepithelial telocytes are an important source of Wnts that supports intestinal crypts. *Nature* 557, 242-246. 10.1038/s41586-018-0084-4 29720649 PMC5966331

[DEV199683C76] Stamataki, D., Holder, M., Hodgetts, C., Jeffery, R., Nye, E., Spencer-Dene, B., Winton, D. J. and Lewis, J. (2011). Delta1 expression, cell cycle exit, and commitment to a specific secretory fate coincide within a few hours in the mouse intestinal stem cell system. *PLoS ONE* 6, e24484. 10.1371/journal.pone.0024484 21915337 PMC3168508

[DEV199683C77] Steinbrecher, K. A., Harmel-Laws, E., Sitcheran, R. and Baldwin, A. S. (2008). Loss of epithelial RelA results in deregulated intestinal proliferative/apoptotic homeostasis and susceptibility to inflammation. *J. Immunol.* 180, 2588-2599. 10.4049/jimmunol.180.4.2588 18250470

[DEV199683C78] Stratis, A., Pasparakis, M., Markur, D., Knaup, R., Pofahl, R., Metzger, D., Chambon, P., Krieg, T. and Haase, I. (2006). Localized inflammatory skin disease following inducible ablation of I kappa B kinase 2 in murine epidermis. *J. Invest. Dermatol.* 126, 614-620. 10.1038/sj.jid.5700092 16397523

[DEV199683C79] Taniguchi, K. and Karin, M. (2018). NF-kappaB, inflammation, immunity and cancer: coming of age. *Nat. Rev. Immunol.* 18, 309-324. 10.1038/nri.2017.142 29379212

[DEV199683C80] Tetteh, P. W., Farin, H. F. and Clevers, H. (2015). Plasticity within stem cell hierarchies in mammalian epithelia. *Trends Cell Biol.* 25, 100-108. 10.1016/j.tcb.2014.09.003 25308311

[DEV199683C81] Tomann, P., Paus, R., Millar, S. E., Scheidereit, C. and Schmidt-Ullrich, R. (2016). Lhx2 is a direct NF-kappaB target gene that promotes primary hair follicle placode down-growth. *Development* 143, 1512-1522. 26952977 10.1242/dev.130898 PMC6514410

[DEV199683C82] van der Flier, L. G., van Gijn, M. E., Hatzis, P., Kujala, P., Haegebarth, A., Stange, D. E., Begthel, H., van den Born, M., Guryev, V., Oving, I. et al. (2009). Transcription factor achaete scute-like 2 controls intestinal stem cell fate. *Cell* 136, 903-912. 10.1016/j.cell.2009.01.031 19269367

[DEV199683C83] van Es, J. H., Jay, P., Gregorieff, A., van Gijn, M. E., Jonkheer, S., Hatzis, P., Thiele, A., van den Born, M., Begthel, H., Brabletz, T. et al. (2005). Wnt signalling induces maturation of Paneth cells in intestinal crypts. *Nat. Cell Biol.* 7, 381-386. 10.1038/ncb1240 15778706

[DEV199683C84] van Es, J. H., Sato, T., van de Wetering, M., Lyubimova, A., Yee Nee, A. N., Gregorieff, A., Sasaki, N., Zeinstra, L., van den Born, M., Korving, J. et al. (2012). Dll1+ secretory progenitor cells revert to stem cells upon crypt damage. *Nat. Cell Biol.* 14, 1099-1104. 10.1038/ncb2581 23000963 PMC3789123

[DEV199683C85] Vereecke, L., Vieira-Silva, S., Billiet, T., van Es, J. H., Mc Guire, C., Slowicka, K., Sze, M., van den Born, M., De Hertogh, G., Clevers, H. et al. (2014). A20 controls intestinal homeostasis through cell-specific activities. *Nat. Commun.* 5, 5103. 10.1038/ncomms6103 25267258

[DEV199683C86] Vlantis, K., Wullaert, A., Sasaki, Y., Schmidt-Supprian, M., Rajewsky, K., Roskams, T. and Pasparakis, M. (2011). Constitutive IKK2 activation in intestinal epithelial cells induces intestinal tumors in mice. *J. Clin. Invest.* 121, 2781-2793. 10.1172/JCI45349 21701067 PMC3223831

[DEV199683C87] Vlantis, K., Wullaert, A., Polykratis, A., Kondylis, V., Dannappel, M., Schwarzer, R., Welz, P., Corona, T., Walczak, H., Weih, F. et al. (2016). NEMO Prevents RIP Kinase 1-Mediated Epithelial Cell Death and Chronic Intestinal Inflammation by NF-kappaB-Dependent and -Independent Functions. *Immunity* 44, 553-567. 10.1016/j.immuni.2016.02.020 26982364 PMC4803910

[DEV199683C88] Xu, M., Horrell, J., Snitow, M., Cui, J., Gochnauer, H., Syrett, C. M., Kallish, S., Seykora, J. T., Liu, F., Gaillard, D. et al. (2017). WNT10A mutation causes ectodermal dysplasia by impairing progenitor cell proliferation and KLF4-mediated differentiation. *Nat. Commun.* 8, 15397. 10.1038/ncomms15397 28589954 PMC5467248

[DEV199683C89] Yilmaz, Z. B., Weih, D. S., Sivakumar, V. and Weih, F. (2003). RelB is required for Peyer's patch development: differential regulation of p52-RelB by lymphotoxin and TNF. *EMBO J.* 22, 121-130. 10.1093/emboj/cdg004 12505990 PMC140043

[DEV199683C90] Yu, H. M., Jerchow, B., Sheu, T. J., Liu, B., Costantini, F., Puzas, J. E., Birchmeier, W. and Hsu, W. (2005). The role of Axin2 in calvarial morphogenesis and craniosynostosis. *Development* 132, 1995-2005. 10.1242/dev.01786 15790973 PMC1828115

[DEV199683C91] Zaph, C., Troy, A. E., Taylor, B. C., Berman-Booty, L. D., Guild, K. J., Du, Y., Yost, E. A., Gruber, A. D., May, M. J., Greten, F. R. et al. (2007). Epithelial-cell-intrinsic IKK-beta expression regulates intestinal immune homeostasis. *Nature* 446, 552-556. 10.1038/nature05590 17322906

[DEV199683C92] Zhang, Y., Tomann, P., Andl, T., Gallant, N. M., Huelsken, J., Jerchow, B., Birchmeier, W., Paus, R., Piccolo, S., Mikkola, M. L. et al. (2009). Reciprocal requirements for EDA/EDAR/NF-kappaB and Wnt/beta-catenin signaling pathways in hair follicle induction. *Dev. Cell* 17, 49-61. 10.1016/j.devcel.2009.05.011 19619491 PMC2859042

[DEV199683C93] Zhao, X., Ma, L., Dai, L., Zuo, D., Li, X., Zhu, H. and Xu, F. (2020). TNFalpha promotes the malignant transformation of intestinal stem cells through the NFkappaB and Wnt/beta catenin signaling pathways. *Oncol. Rep.* 44, 577-588. 10.3892/or.2020.7631 32627006 PMC7336517

