# Supplementary material for: NF-κB determines Paneth versus goblet cell fate decision in the small intestine
Source: Development. 2021 Nov 9;148(21):dev199683. doi: 10.1242/dev.199683 (PMC8627599; doi:10.1242/dev.199683)
Supplement: Supplementary information [file develop-148-199683-s1.pdf]

SUPPLEMENTARY METHODS

In situ hybridization probes

In situ hybridization was carried out using the following mouse cDNA probe templates: *Axin2* (nt 282 – 918 of *Axin2* cDNA; BC057338) kindly provided by J. Huelsken, ISREC, Lausanne, Switzerland/W. Birchmeier, MDC, Berlin, Germany. *Cryptidin-1* (nt 3 – 401 of NM_010031/AA871421.1; I.M.A.G.E. ID 1096215), kindly provided by M. van de Wetering/H. Clevers, Hubrecht Institute, Utrecht Netherlands. Murine *Nfkbia* (IκBα; nt 1 – 1091; U36277/NM010907). *Olfm4* (NM_001030294), kindly provided by J. Heuberger/W. Birchmeier, MDC, Berlin, Germany. pBSK-*Sox9* (first 1800bp of *Sox9* cDNA), kindly provided by T. Willnow, MDC, Berlin, Germany. NM_011448). *Wnt10a* (nt 1295 - 2487, U61969), kindly provided by G. Shackleford, UCLA, USA).

Primers used for quantitative RT-PCR

Primer name	Sequence	Origin
mLysozyme	forward: 5'-GCAGCCATACAATGTGCAAAGAGG-3'<br>reverse: 5'-TTTGCCCTGTTTCTGCTGAAGTCC-3'	(Heuberger et al. 2014)
mCryptdin-1	forward: 5'-AGG AGC AGC CAG GAG AAG-3'<br>reverse: 5'-ATG TTC AGC GAC AGC AGA G-3'	(Tsai et al. 2014)
mMath1	forward: 5'-GTTGCGCTCACTCACAAATAAGGG-3'<br>reverse: 5'-TGGCAGTTGAGTTTCTTCAAGGCG-3'	(Heuberger et al. 2014)
mDll4	forward: 5'-TTA CTG CAG CAA GCC AGA TG-3'<br>reverse: 5'-CAT TCT TGC ACG GAG AGT GG-3'	(Heuberger et al. 2014)
mAscl2	forward: 5'-AAAGCTTGGTCCGGTTCTTCATCC-3'<br>reverse: 5'-GCAGATGCTTAGCTTATTGCGTCC-3'	(Heuberger et al. 2014)
mLgr5	forward: 5'-CCTACTCGAAGACTTACCCAGT-3'<br>reverse: 5'-GCATTGGGGTGAATGATAGCA-3'	(Heuberger et al. 2014)
mOlfm4	forward: 5'-CAAGCCTGGCTCGACGGCC-3'<br>reverse: 5'-CGCGAACATCTTCAGGTTCT-3'	(Heuberger et al. 2014)
mSpedf1	forward: 5'-AACATGTATCCCGACGATAGCAGC-3'<br>reverse: 5'-TCAATATCTTTCAGGACCTCGCCC-3'	(Heuberger et al. 2014)
mGob5	forward: 5'-TGAAATTGTGCTGCTGACCGATGG-3'<br>reverse: 5'-TGCTGCGAAAGCATCAACAAGACC-3'	(Heuberger et al. 2014)

Development • Supplementary information

mKlf4	forward: 5'-GAGTTCCTCACGCCAACG-3'<br>reverse: 5'-CGGGAAGGGAGAAGACACT-3'	(Al Alam et al. 2015)
mMuc2	forward: 5'-TGTGATGCCAATGACAAGGTGTCC-3'<br>reverse: 5'-ACCACAATGTTGATGCCAGACTCG-3'	(Heuberger et al. 2014)
mAxin2	forward: 5’-ACTGACCGACGATTCCATGT-3’<br>reverse: 5’-CTGCGATGCATCTCTCTCTG-3’	NM_015732.4
mSST	forward: 5’-ACCGGGAAACAGGAACTGG-3’<br>reverse: 5’-TTGCTGGTTCGAGTTGGC-3’	(Mustata et al. 2011)
mChromoA	forward: 5’-TCCCCACTGCAGCATCCAGTTC-3’<br>reverse: 5’-CCTTCAGACGGCAGAGCTTCGG-3’	(Mustata et al. 2011)
mDll1	forward: 5’-CCGATGACCTCGCAACAGAA-3’<br>reverse: 5’-CCAGGGTCGCACATCTTCTC-3’	(Zhang et al. 2019)
mHes1	forward: 5’-GGAGAAGAGGCGAAGGGCAAGA-3’<br>reverse: 5’CGT GGACAGGAAGCGGGTCA-3’	(Zhang et al. 2019)
mGfi1	forward: 5’-TCCACACTGTCCACACACCT-3´<br>reverse: 5´-CTGGCACTTGTGAGGCTTCT-3´	(Zhang et al. 2019)
mWnt3a	forward: 5’-TGGAACTGTACCACCATAGATGAC-3’<br>reverse: 5’-ACACCAGCCGAGAGCGATG-3’	(Farin et al. 2012)
mWnt10a	forward: 5’-GAGAGCCTCACAGAGACATCCAT-3’<br>reverse: 5’-TACTGTGCGGAACTCAGGCGT-3’	NM_009518
mSox9	forward: 5’-GACTCCCCACATTCCTC-3’<br>reverse: 5’-CCTCTCGCTTCAGATCAAC-3’	NM_011448
mCtnnb1	forward: 5’-TGCCATCTGTGCTCTTCGTC-3’<br>reverse: 5’-AACTGCTGCTGCGTTCCAC-3’	NM_007614
mIcam1	forward: 5’-CTGCGTTTTGGAGCTAGCGG-3’<br>reverse: 5’-TTGGCTCCCTTCCGAGACCT-3’	NM_010493
mTNF	forward: 5’-TCCCAAATGGCCTCCCTCTCC-3’<br>reverse: 5’-CCACTTGGTGGTTTGCTACGA-3’	NM_013693
mIL6	forward: 5’-ACAAAGCCAGAGTCCTTCAGAGA-3’<br>reverse: 5’-AGCCACTCCTTCTGTGACTCC-3’	NM_031168
mIL1α	forward: 5’-GGAGAGCCGGGTGACAGTATC-3’<br>reverse: 5’-TCTGGGTTGGATGGTCTCTTC-3’	NM_010554.4
mGAPDH	forward: 5'-AGCAAGGACACTGAGCAAGAG-3'<br>reverse: 5'-GCAGCGAACTTTATTGATGGT-3'	NM_008084
mHprt	forward: 5’-GGATATGCCCTTGACTATAATGAG-3’<br>reverse: 5’- GGCAACATCAACAGGACTC-3’	NM_013556
mNfkbia	forward: 5’-AGGAGTACGAGCAAATGG-3’<br>reverse: 5’-CAGGCAAGATGTAGAGGG-3’	NM_010907
mCXCL12	forward: 5’-TCTTCGAGAGCCACATCGCC-3’<br>reverse: 5’-AGCCGTGCAACAATCTGAAGG-3’	NM_021704.3
mLcn2	forward: 5´-ACTTCCGGAGCGATCAGTTC-3´<br>reverse: 5´-TTTTTCTGGACCGCATTGCC-3´	NM_008491.1
mNos2	forward: 5´- TTTCACCCGCTTTGCCAAGT-3´<br>reverse: 5´- GTCTCTGCGCATCCCAGTCA-3´	NM_001313921.1

Development • Supplementary information

mNox1	forward: 5´- CTCCAGCCTATCTCATCCTGAG-3´<br>reverse: 5´- AGTGGCAATCACTCCAGTAAGGC-3´	NM_172203.2
mTNFIP3	forward: 5´- GTCAGGAAGCTCGTGGCTCT-3´<br>reverse: 5´- TTAAGGGTGCTGCAGAGGGC-3´	NM_001166402.1
mLbp	forward: 5´- TGCTGTTTGCTGCAGACAAC-3´<br>reverse: 5´- TGGGTCCAACCAAAACCTTC-3´	NM_008489.2
mSmoc2	forward: 5´ AGCTGGGGCAATTCTTTCAG-3´<br>reverse: 5´- AATGAGCAAAGGCCTTCTGC-3´	NM_022315.2
mLgr1	forward: 5´- ATTTGATGGTCTGTCGCGGT-3´<br>reverse: 5´- GTGCAGCACGTGCATCTTAG-3´	(Heuberger et al. 2014)
mTert	forward: 5´-AGAAACGTGCTGGCTTTTGG-3´<br>reverse: 5´- AACAGTGTTGGGCAAGTAGC-3´	NM_009354.2
mHopx	forward: 5´- AGTTCCTTCCCTTACAGCTGTG-3´<br>reverse: 5´-ACTTGCTTTTCTGCCCCTTG-3´	NM_001159900.1
mCcdn1	forward: 5´- AGTTCATTTCCAACCCACCC-3´<br>reverse: 5´- AGACCAGCCTCTTCCTCCAC-3´	NM_001379248.1
mEdn1	forward: 5´- ACTACGAAGGTTGGAGGCCA-3´<br>reverse: 5´- CAATGTGCTCGGTTGTGCGT-3´	NM_010104.4
mProm1	forward: 5´- CTGAAGATTGCCCTCTATGA-3´<br>reverse: 5´- AGTTTCTGGGTCCCTTTGAT-3´	NM_001163577.1
mEphb3	forward: 5´- GACCTTGCTGCCCGAAACAT-3´<br>reverse:5´- CCCACATGACAATCCCATAGCT-3´	NM_010143.1
mMsi1	forward: 5´- AAAACCACCAACAGGCACAG-3´<br>reverse: 5´- TGGGCTTTCTTGCATTCCAC-3´	NM_001376960.1
mTnfrsf19	forward: 5´- TGAAAGTGGCGGTGAATGTG-3´<br>reverse: 5´- AACATTCACAGCCAGGCTTC-3´	NM_001164155.1

Antibodies

- Non-phospho (active) β-Catenin (Ser33/37/Thr41), rabbit, Cell Signaling (#8814), 1:500
- BrdU, mouse, Sigma (BU33), 1:500
- Chromogranin A, rabbit, Abcam (15160), 1:300
- Cleaved Caspase-3 (Asp175), rabbit, Cell Signaling (9664), 1:400
- Digoxigenin-AP Fab fragments, sheep, Roche (11093274910), 1:1000
- E-Cadherin, mouse, BD (610181), 1:500
- EGFP, chicken, Abcam (ab13970), 1:400
- Ki67, rabbit, Abcam (ab15580), 1:100

- Ldha (Lactate dehydrogenase A), Santa Cruz (sc-27230), 1:1000
- Lysozyme, rabbit, DAKO (A 0099), 1:1000
- Mmp7 (matrix metalloproteinase-7), Santa Cruz sc-515703, 1:1000
- Muc2 (Mucin 2), rabbit, Santa Cruz (sc-15334), 1:100
- Olfm4, rabbit, Cell signaling (D6Y5A), 1:400
- Parp1 (Poly(ADP-ribose)-polymerase 1), mouse, Santa Cruz (sc-8007), 1:1000
- Sox9 (SRY-box 9), rabbit, Santa Cruz (sc-20095) 1:100, (Vidal et al. 2005; Nowak et al. 2008), and rabbit, Millipore (AB5535), 1:200.

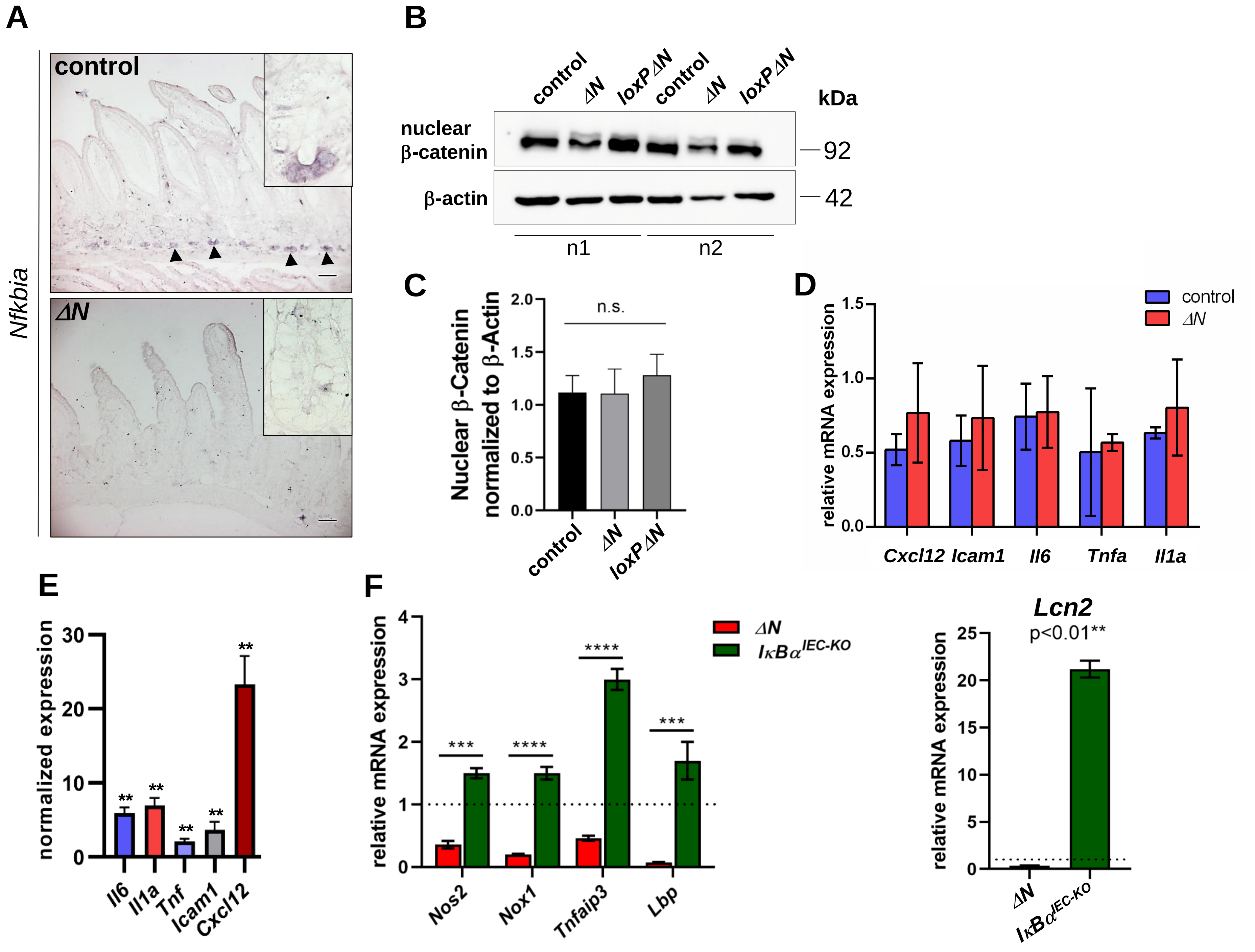

A
control
ΔN
Nfkbia
B
control
ΔN
loxP ΔN
control
ΔN
loxP ΔN
kDa
nuclear β-catenin
β-actin
92
42
n1
n2
C
Nuclear β-Catenin normalized to β-Actin
n.s.
2.0
1.5
1.0
0.5
0.0
control
ΔN
loxP ΔN
D
relative mRNA expression
control
ΔN
Cxcl12
Icam1
Il6
Tnfa
Il1a
E
normalized expression
**
Il6
Il1a
Tnf
Icam1
Cxcl12
F
relative mRNA expression
ΔN
IκBα IEC-KO
***
****
Nos2
Nox1
Tnfaip3
Lbp
Lcn2
p<0.01**

Fig. S1. (**A**) **NF-κB activity is suppressed in SI crypts of *ΔN* mice.** *In situ* hybridization on PSI sections of control and *ΔN* mice using a riboprobe for mouse *Nfkbia* (NF-κB inhibitor IκBα, bona fide target gene of NF-κB). Arrowheads point to *Nfkbia* mRNA expression. Scale bars = 50µm. Note that in *ΔN* mice a C-terminal deletion mutant of the human IκBα was used which is not detected by the murine *Nfkbia* riboprobe (see (Schmidt-Ullrich et al. 2001; Schmidt-Ullrich et al. 2006)). (**B – C**) **β-catenin protein expression is maintained in *ΔN* and *loxP-ΔN* mice.** (**B**) PSI whole cell protein extracts of control, *ΔN* and *loxP-ΔN* mice (n=3/group n1 or n2) were used for SDS-PAGE Western blotting with an antibody against nuclear β-catenin. β-actin was used as loading control. (**C**) Quantification of nuclear β-catenin protein expression normalized to β-actin (from (**B**)). n.s.= not significant, *p=0.658* (one-way Anova), error bars = SEM. Note that *ΔN* and *loxP-ΔN* mice maintain levels of nuclear β-catenin protein expression that are identical to controls, in spite of one-allelic expression from *Ctnnb1* gene (see (Schmidt-Ullrich et al. 2001)). (**D - E**) **No increase in inflammatory cytokines in small intestines of *ΔN* mice**. (**D**) Quantitative real-time PCR (qRT–PCR) for inflammatory cytokines (*Cxcl12, Icam, Il6*, *Tnfa*, and *Il1a*) using RNA from PSIs of *ΔN* and control mice (n=3/group). Observed differences were statistically insignificant. Error bars = SEM. (**E**) qRT–PCR for inflammatory cytokines (*Il6*, *Il1a*, *Tnfa*, *Icam1*, *Cxcl12*) using RNA from bulk organoids from $I\kappa B\alpha^{IEC\text{-}KO}$ mice (n=4; constitutively increased NF-κB activity in the SI epithelium; used as positive control for (**D**)). mRNA expression was normalized to 2 reference genes, *Hprt1* and *Sdha* (Succinate dehydrogenase complex, subunit A). Expression of control animals (n=4) was normalized to 1. Mean ratio of $I\kappa B\alpha^{IEC\text{-}KO}$ to control is shown. **: $p<0.01$, error bars = SEM. (**F**) Quantitative real-time PCR (qRT–PCR) for NF-κB target genes that are known to be upregulated in inflammatory bowel disease (IBD; see (Mikuda et al. 2020)). Left panel: *Nos2* (Nitric Oxide Synthase), *Nox1* (NADPH Oxidase 1), *Tnfaip3* (A20; Tumor Necrosis Factor alpha-induced protein 3), *Lbp* (Lipopolysaccharide Binding Protein). Right panel: *Lcn2* (NGAL, Lipocalin 2). RNA from PSIs of *ΔN*, $I\kappa B\ \alpha^{IEC\text{-}KO}$ and control mice (n=3/group) was used. Expression of control animals was normalized to 1. ***: $p<0.001$, ****: $p<0.0001$, error bars = SEM.

Development • Supplementary information

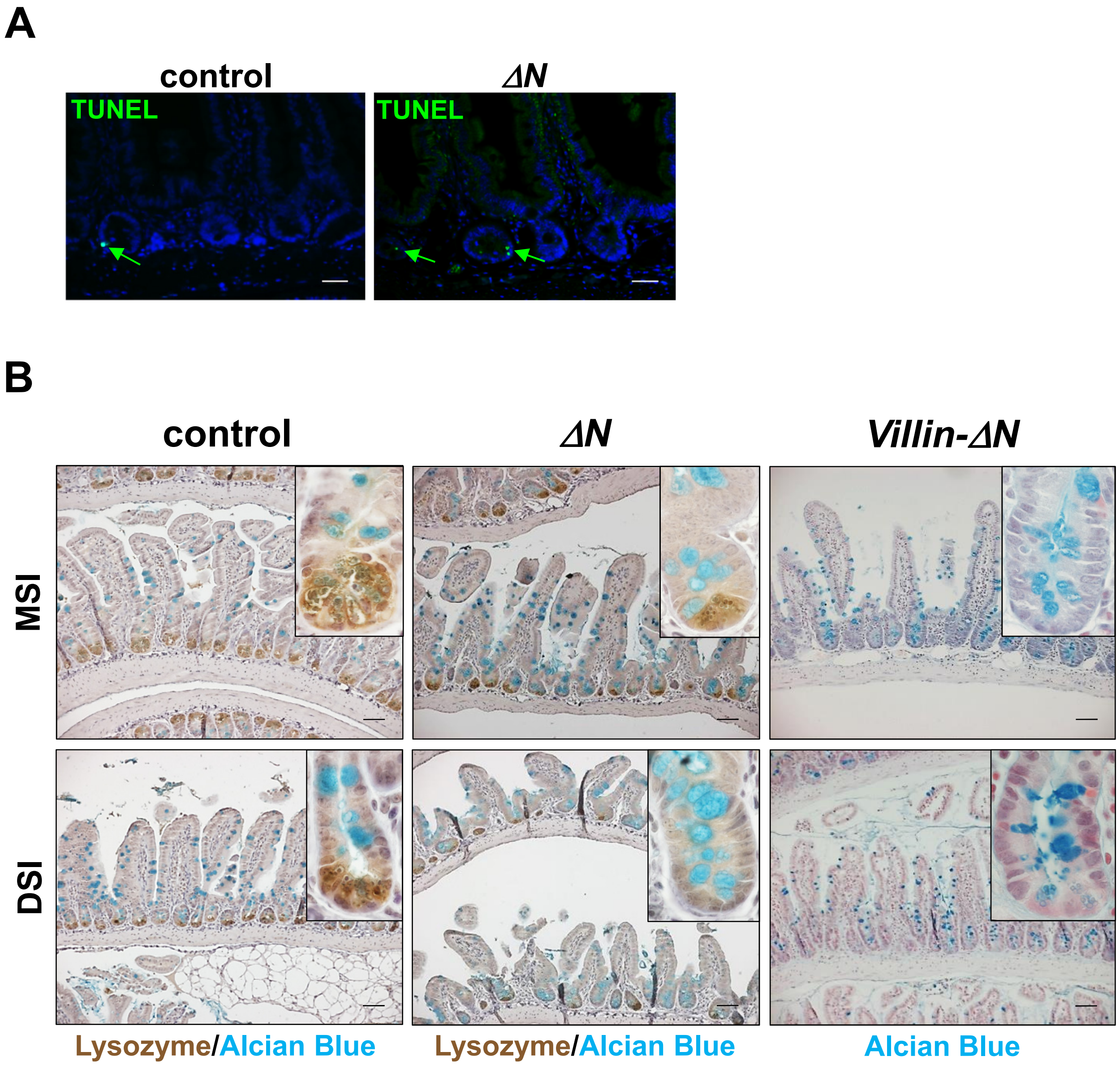

Fig. S2. (**A**) **No increased apoptosis in SI epithelium of** ***ΔN*** **mice.** IF TUNEL staining on PSI sections of *ΔN* mice and controls (n=4/ group). Green arrows point to TUNEL-positive cells**.** Nuclear counterstaining: DAPI. Scale bars = 50 µm. (**B**) **Increased numbers of Alcian blue-positive (goblet) cells also in the jejunum and ileum of** ***ΔN*** **mice.** Sections of middle (MSI, jejunum) and distal (DSI, ileum) SI of control, *ΔN* and *Villin-ΔN* mice (n=3/group) stained either with Alcian Blue (blue) alone or together with an anti-Lysozyme antibody (brown; DAB). Scale bars = 50µm.

Development • Supplementary information

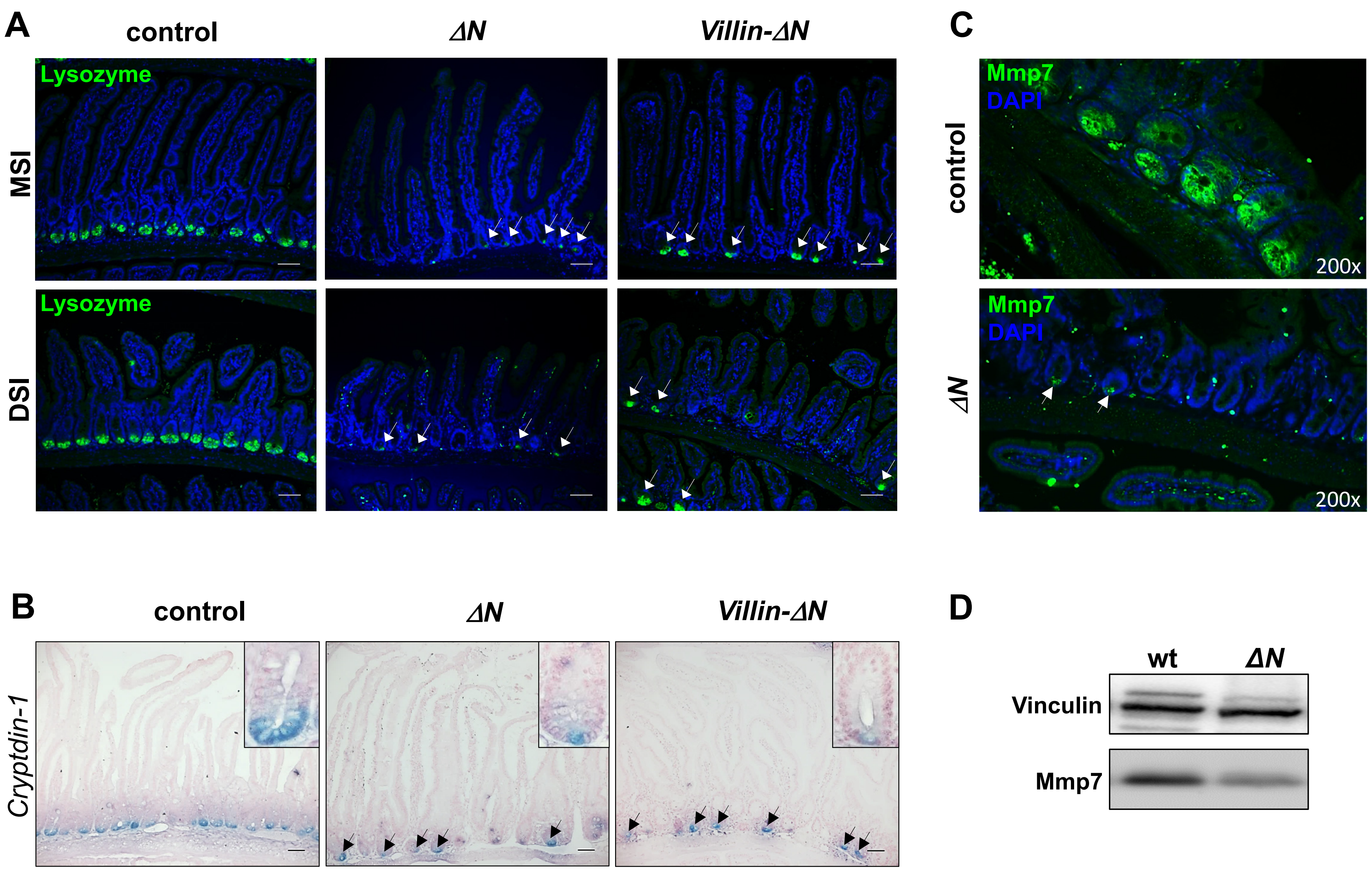

Development • Supplementary information

Fig. S3. **Reduced numbers of Paneth cells also in the jejunum and ileum of *ΔN* mice.** (**A**) Anti-Lysozyme antibody staining (green) on sections of middle (MSI, jejunum) and distal (DSI, ileum) SI of control, *ΔN* and *Villin-ΔN* mice (n=3/group). White arrows indicate remaining Paneth cells in *ΔN* and *Villin-ΔN* crypts. Scale bars = 50µm. (**B – D**) **Paneth cell loss in *ΔN* mice was further verified by strongly reduced Cryptdin-1 and MMP7 expression.** (**B**) In situ hybridization using a riboprobe for *Cryptdin-1* on PSI sections of control, *ΔN* and *Villin-ΔN* mice (n=3/group). Black arrows indicate remaining Paneth cells in PSI crypts of *ΔN* and *Villin-ΔN* mice. Due to one Paneth cell/crypt at most, Cryptdin-1 expression is strongly reduced in *ΔN* and *Villin-ΔN* crypts when compared to controls (see insets). Scale bars = 50µm. (**C**) Anti-Mmp7 antibody staining on PSI sections of control and *ΔN* mice (n=3/group). White arrows indicate remaining Paneth cells in *ΔN* mice which express Mmp7. Original magnification 200x. (**D**) Comparing protein expression of Mmp7 in *ΔN* and control mice (n=3/group). PSI whole cell extracts of control and *ΔN* mice were used for SDS-PAGE Western blotting.

Development • Supplementary information

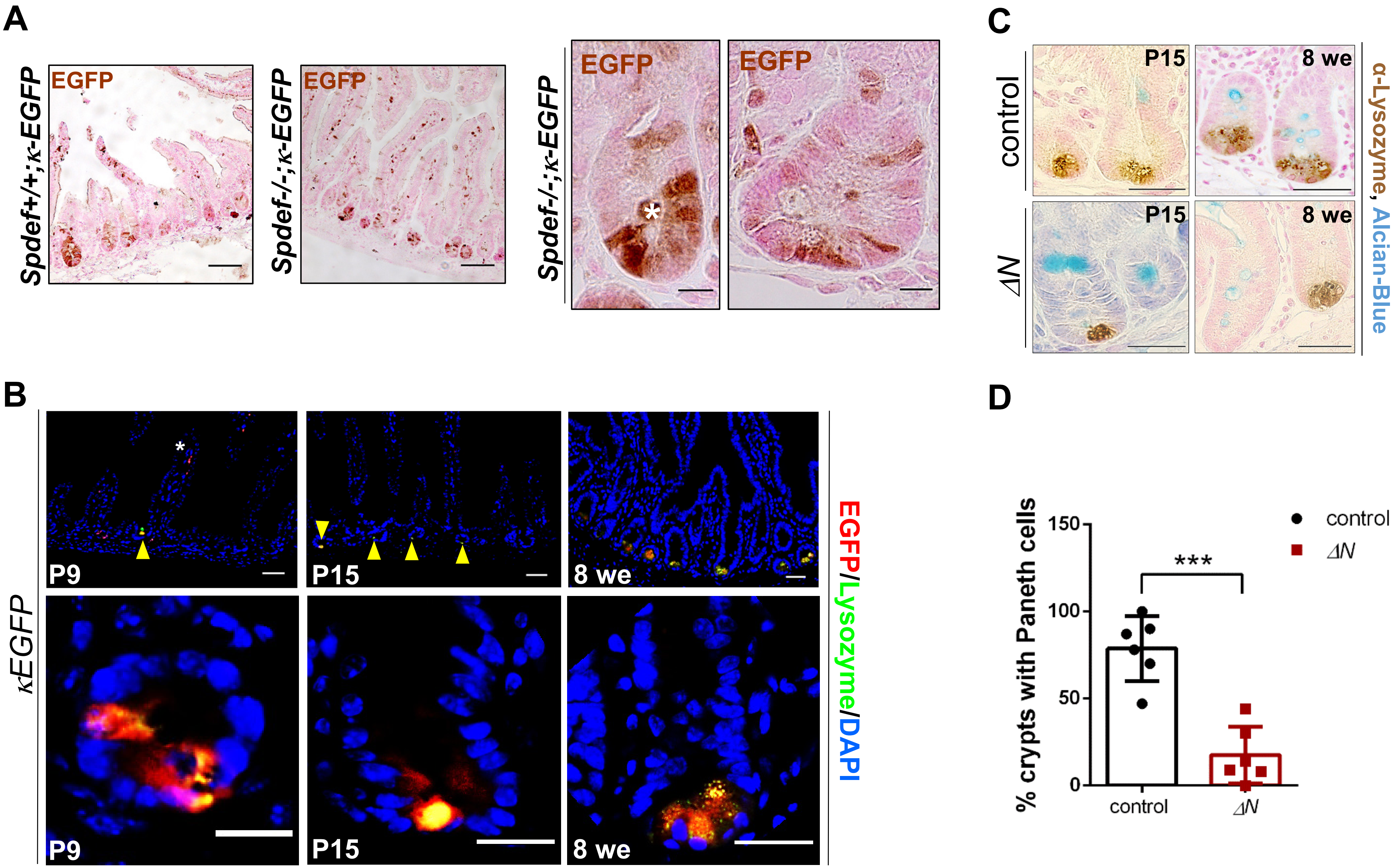

Development • Supplementary information

Fig. S4. (**A**) **NF-κB remains active in the absence of goblet and Paneth cell maturation factor Spdef1.** (**A**) IHC using anti-EGFP antibody on PSI sections of *Spdef-/- ;κ-EGFP* (Spdef KO) and *Spdef+/+;κ-EGFP* (controls) mice (n=3/group). Scale bars = 50µm and 10µm (right panels). White asterisk indicates background staining. (**B – D**) **NF-κB activity is required for postnatal Paneth cell differentiation.** (**B**) Anti-EGFP (red) and -Lysozyme (green) antibody staining on PSI sections of *κ-EGFP* mice at P9, P15 and 8 weeks of age (n=3/group). Nuclear counterstain: DAPI (blue). NF-κB activity is already detected in

Paneth cell precursors at P9. Yellow arrows point to Paneth cells with active NF-κB (P9 and P15). Scale bars upper panels = 50µm, lower panels = 20µm. (**C**) IHC on PSI sections of control and *ΔN* mice (n=3/group) stained with Alcian Blue and with anti-Lysozyme antibody (brown; DAB). While in controls the number of Paneth cells/crypt increases between P15 and 8 weeks of age, this is not the case in *ΔN* mice. Scale bars = 20µm. (**D**) Transmission electron microscopy of PSI crypts of *ΔN* and control mice: Quantification of crypts containing Paneth cells in *ΔN* mice compared to controls (n=4/group) confirms data shown in Fig. 2. ****: $p<0.0001$, error bars = SEM.

Development • Supplementary information

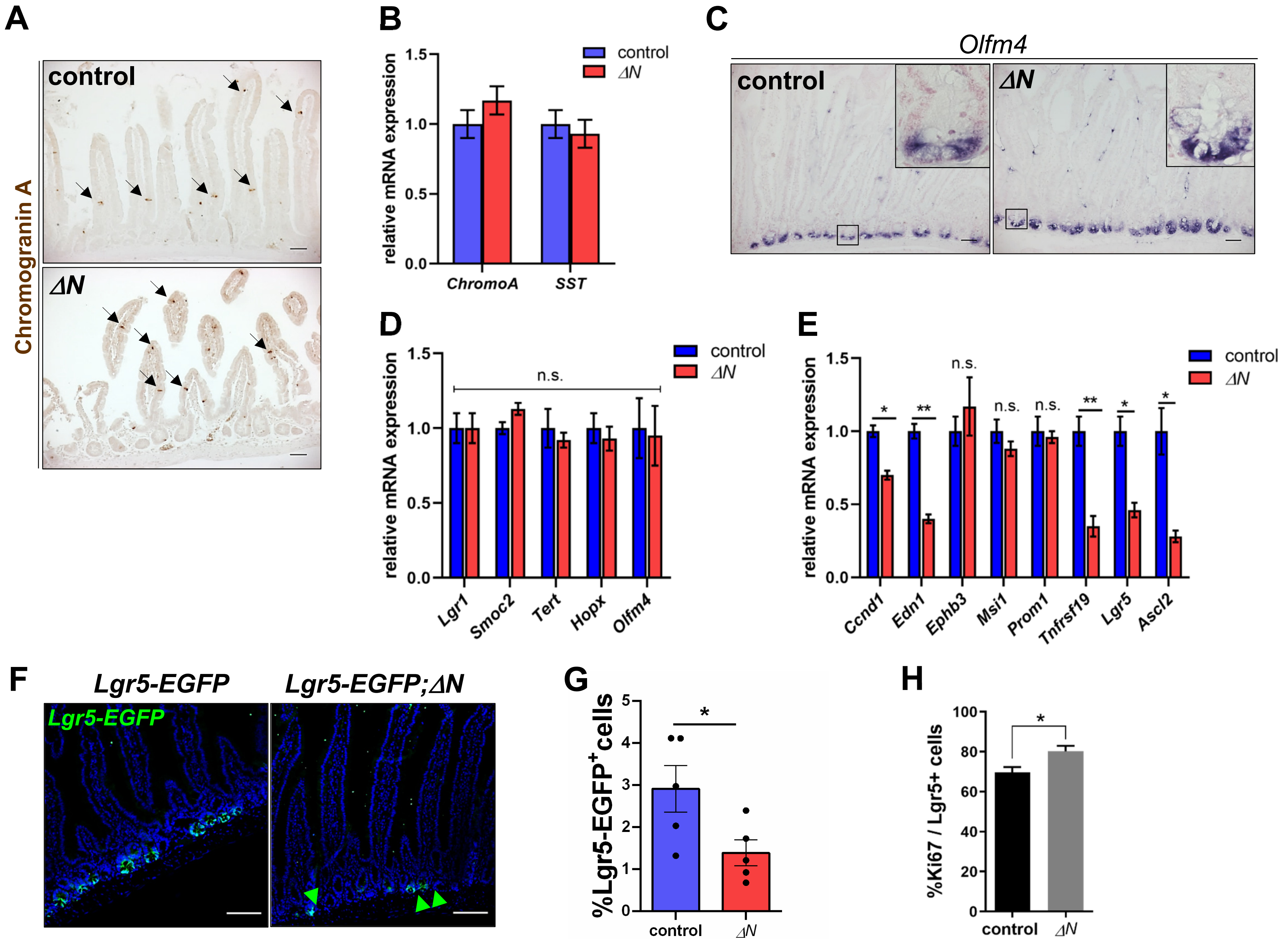
A
control
ΔN
Chromogranin A
B
relative mRNA expression
control
ΔN
ChromoA
SST
C
Olfm4
control
ΔN
D
n.s.
Lgr1
Smoc2
Tert
Hopx
Olfm4
E
Ccnd1
Edn1
Ephb3
Msi1
Prom1
Tnfrsf19
Lgr5
Ascl2
F
Lgr5-EGFP
Lgr5-EGFP;ΔN
G
%Lgr5-EGFP+cells
H
%Ki67 / Lgr5+ cells

Fig. S5. (**A, B**) **Unaltered numbers of enteroendocrine cells in *ΔN* mice.** (**A**) IHC using an anti-Chromogranin-A antibody on PSI sections of control and *ΔN* mice (n=3/group). Arrows point to enteroendocrine cells. Scale bars = 50µm. (**B**) qRT–PCR for enteroendocrine markers *Chromogranin-A* (*ChromoA*) and *Somatostatin* (*SST*) using RNA isolated from PSIs of *ΔN* and control mice (n=3/group). Error bars = SEM. (**C – H**) **Altered expression of Wnt-dependent ISC markers in *ΔN* mice.** (**C**) In situ hybridization for ISC marker *Olfm4* on PSI (proximal small intestine; duodenum) sections of *ΔN* and control mice (n=3/group). (**D**, **E**) qRT–PCR for Wnt-independent (**D**) and -dependent (**E**) ISC markers (see (Mikuda et al. 2020)), using

RNA from PSIs of control and *ΔN* mice (n=3/group). Lgr1: Leucine-rich repeat-containing G-protein coupled receptor 1; Smoc2: SPARC-related modular calcium-binding protein 2; Tert: Telomerase reverse transcriptase; Hopx: Homeodomain-only protein homeobox; Olfm4: Olfactomedin 4; Ccnd1: Cyclin D1; Edn1: Endothelin 1; Ephb3: Ephrin type-B receptor 3; Msi1: Musashi RNA-binding protein 1; Prom1: Prominin 1; Tnfrsf19: Troy, TNF receptor superfamily member 19; Lgr5: Leucine-rich repeat-containing G-protein coupled receptor 5; Ascl2: Achaete scute-like 2; n.s. = not significant, *: p<0.05, **: p<0.01; error bars = SEM. (**F**) IF using an anti-EGFP (green) antibody on PSI sections of *Lgr5-EGFP* and *Lgr5-EGFP;ΔN* mice (n=3/group). Nuclear counterstain: DAPI (blue). Green arrowheads point to scarce Lgr5 expression in crypts of *ΔN* mice. Scale bar = 100µm. (**G**) Quantification (in %) of EGFP-positive (Lgr5-expressing) cells in *ΔN* compared to control mice (n=5/group) by FACS. *: p<0.05; error bars = SEM. (**H**) Quantification (in %) of Ki67- versus Lgr5-positive cells in PSI of *Lgr5-EGFP* and *Lgr5-EGFP;ΔN* mice (n=3/group). *: p<0.05; error bars = SEM.

Development • Supplementary information

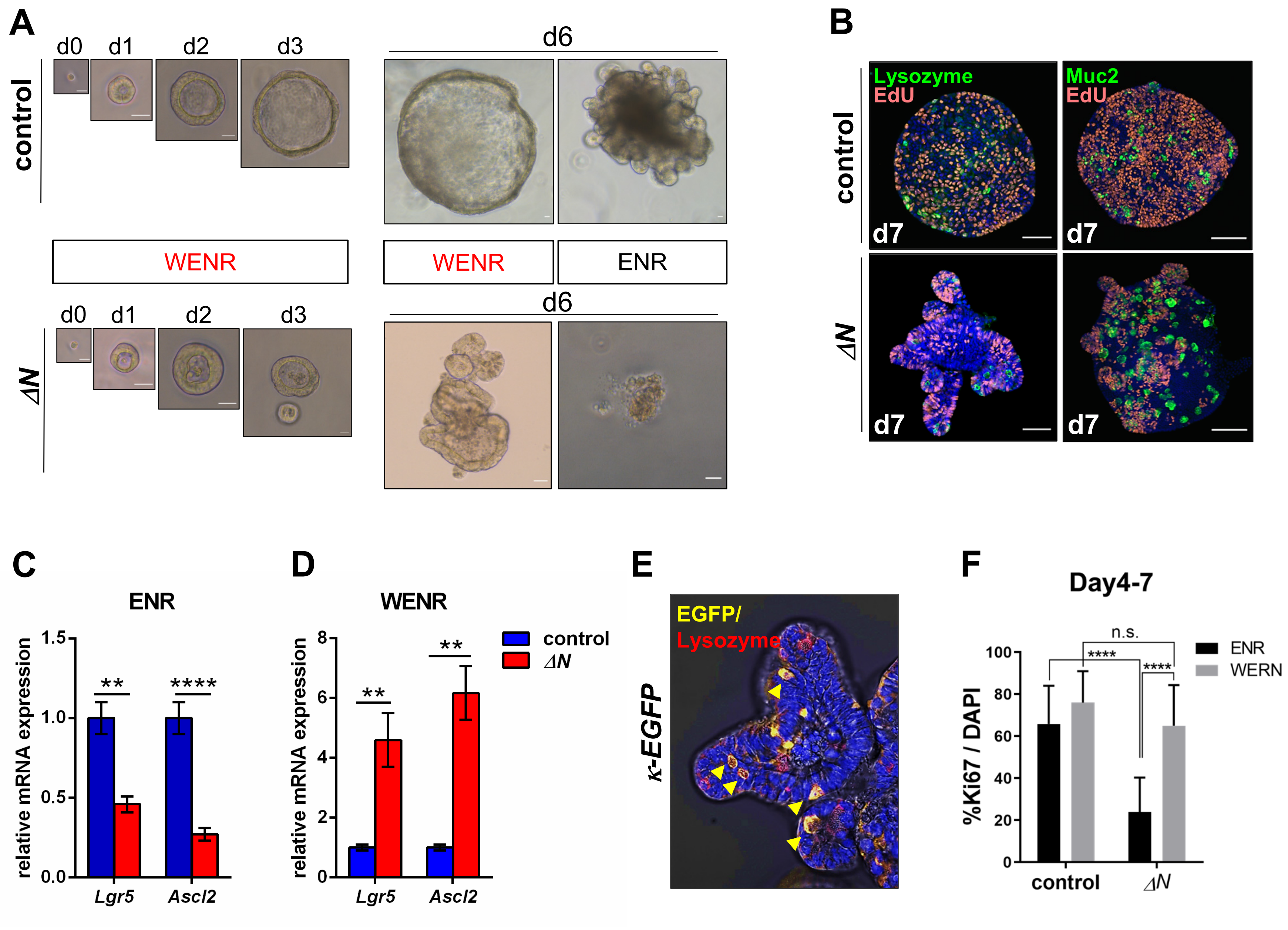

Development • Supplementary information

Fig. S6. Wnt rescues organoid growth in *ΔN* mice. (**A**) Single, EGFP-positive cells isolated from *Lgr5-EGFP* (control) or *Lgr5-EGFP*;*ΔN* mice (*ΔN*) by FACS and cultured in presence (WENR) or absence of Wnt (ENR). n=3 independent experiments; d0 – d6 = days of culture. (**B**) EdU incorporation together with anti-Lysozyme or anti-Muc2 (both green) staining in control and *ΔN* organoids (from 3 mice/group) at culture day 7 (d7) in WENR medium, verified by EdU antibody staining (purple). Nuclear counterstain: DAPI (blue). (**C**, **D**) Representative qRT–PCR for stem cell markers Lgr5 and Ascl2 using RNA isolated from bulk organoids grown either in ENR or WENR medium. **: $p<0.01$, ****: $p<0.0001$; error bars = SEM. (**E**) Representative image of NF-Bκ activity in Paneth cells of crypt organoids: Anti-EGFP and -Lysozyme antibody staining on cultured organoids obtained from *κ-EGFP* mice (n=3). Yellow arrowheads point to Paneth cells. Scale bar = 20μm. (**F**) Quantification (in %) of Ki67-positive cells of bulk control and *ΔN* organoids grown either in ENR or WENR medium. Two-way ANOVA with Bonferroni´s multiple comparison test, ****p<0.0001, n.s. = not significant; error bars = SEM.

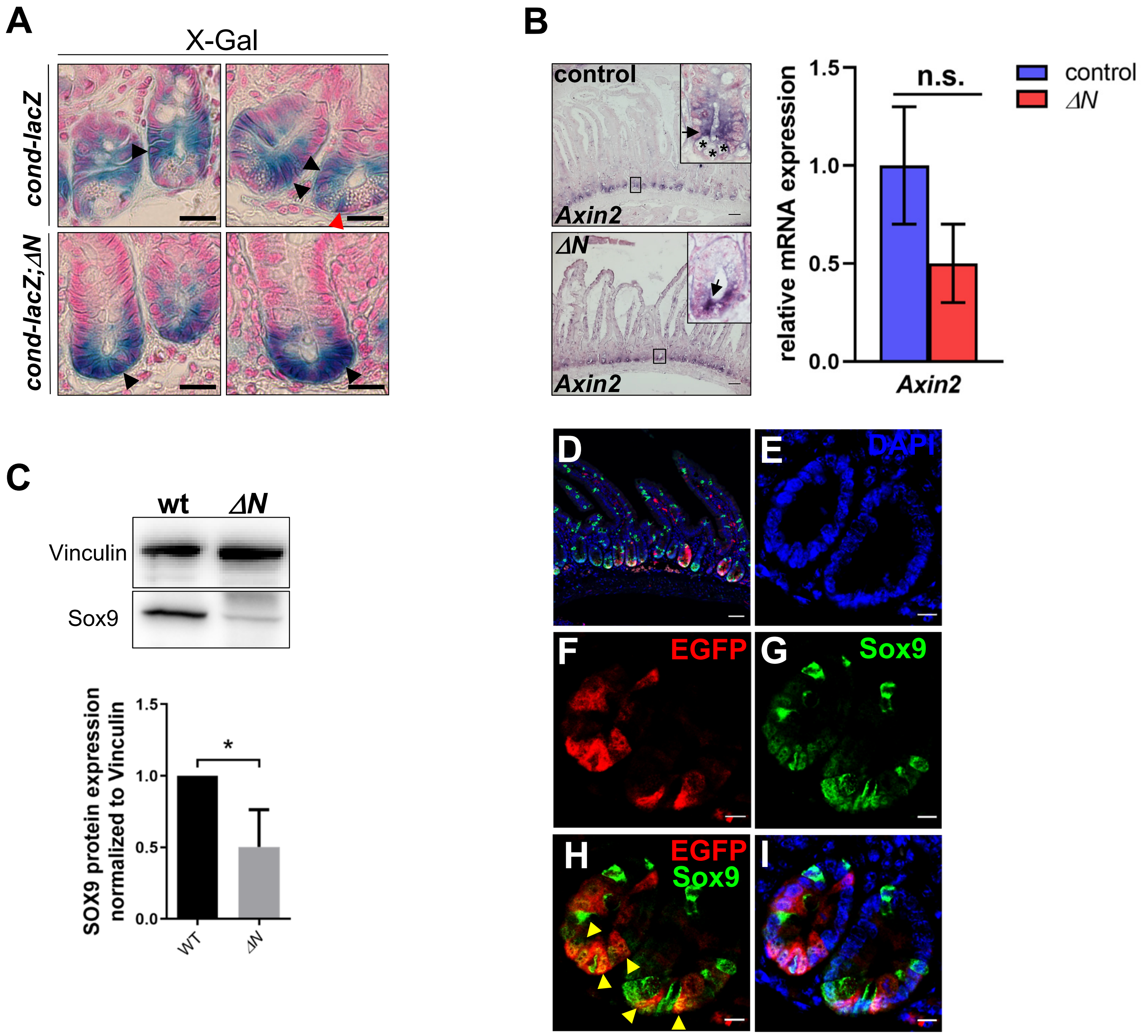

Development • Supplementary information

Fig. S7. (A, B) Overall Wnt/β-Catenin activity appears to be maintained in crypts of *ΔN* mice
(**A**) Cryosections
of whole mount X-Gal-stained (blue) PSIs obtained from WNT reporter *cond-lacZ* (controls) and *cond-lacZ;ΔN* mic e (n=3/group). Black arrows point to β-galactosidase expression (= Wnt activity) in transit amplifying (TA) cells in controls (upper panels) and in the bottom of *ΔN*-positive crypts (lower panels). Red arrow points to CBC stem cells. Scale bars = 30µm. (**B**) Left panels: In situ hybridization for canonical Wnt/β-Catenin target gene *Axin2* on PSI sections of control and *ΔN* mice (n=3/group). Insets: Asterisks in controls indicate Paneth cells. Black arrows point t o *Axin2* in TA cells of controls and to aberrant *Axin2* mRNA expression in *ΔN*-positive crypt bottoms. Scale bars = 50µm. Right panel: qRT–PCR for *Axin2* using RNA from PSIs of *ΔN* and control mice (n=3/group), normalized to 1 (control). n.s., not significant. **(C) Quantification of Sox9 protein in *ΔN*-positive SI epithelium compared to controls.** Upper panel: SDS-PAGE Western blotting using PSI whole cell extracts from control and *ΔN* mice (n=3/group) showing Sox9 protein expression. Lower panel: Quantification of Sox9 protein expression normalized t o vinculin. *: $p<0.05$, error bars = SEM. (**D - I**) **Sox9 protein is co-expressed with EGFP (equivalent to active nu clear NF-κB in the cell).** IF using anti-EGFP and Sox9 antibody co-staining on sections of PSI of *κκ –EGFP* reporter mice (n=3/group). Yellow arrow heads point to
cellular co-localization of Sox9 expression and NF-κB activity (EGFP expression). Nuclear counterstain: DAPI. Scale bars = 10µm.

Development • Supplementary information

SUPPLEMENTARY REFERENCES

Al Alam D, Danopoulos S, Schall K, Sala FG, Almohazey D, Fernandez GE, Georgia S, Frey MR, Ford HR, Grikscheit T et al. 2015. Fibroblast growth factor 10 alters the balance between goblet and Paneth cells in the adult mouse small intestine. *Am J Physiol Gastrointest Liver Physiol* **308**: G678-690.

Farin HF, Van Es JH, Clevers H. 2012. Redundant sources of wnt regulate intestinal stem cells and promote formation of paneth cells. *Gastroenterology* **143**: 1518-1529 e1517.

Heuberger J, Kosel F, Qi J, Grossmann KS, Rajewsky K, Birchmeier W. 2014. Shp2/MAPK signaling controls goblet/paneth cell fate decisions in the intestine. *Proc Natl Acad Sci U S A* **111**: 3472-3477.

Mikuda N, Schmidt-Ullrich R, Kargel E, Golusda L, Wolf J, Hopken UE, Scheidereit C, Kuhl AA, Kolesnichenko M. 2020. Deficiency in IkappaBalpha in the intestinal epithelium leads to spontaneous inflammation and mediates apoptosis in the gut. *J Pathol* **251**: 160-174.

Mustata RC, Van Loy T, Lefort A, Libert F, Strollo S, Vassart G, Garcia MI. 2011. Lgr4 is required for Paneth cell differentiation and maintenance of intestinal stem cells ex vivo. *EMBO Rep* **12**: 558-564.

Nowak JA, Polak L, Pasolli HA, Fuchs E. 2008. Hair follicle stem cells are specified and function in early skin morphogenesis. *Cell Stem Cell* **3**: 33-43.

Schmidt-Ullrich R, Aebischer T, Hulsken J, Birchmeier W, Klemm U, Scheidereit C. 2001. Requirement of NF-kappaB/Rel for the development of hair follicles and other epidermal appendices. *Development* **128**: 3843-3853.

Schmidt-Ullrich R, Tobin DJ, Lenhard D, Schneider P, Paus R, Scheidereit C. 2006. NF-kappaB transmits Eda A1/EdaR signalling to activate Shh and cyclin D1 expression, and controls post-initiation hair placode down growth. *Development* **133**: 1045-1057.

Tsai SY, Sennett R, Rezza A, Clavel C, Grisanti L, Zemla R, Najam S, Rendl M. 2014. Wnt/beta-catenin signaling in dermal condensates is required for hair follicle formation. *Dev Biol* **385**: 179-188.

Vidal VP, Chaboissier MC, Lutzkendorf S, Cotsarelis G, Mill P, Hui CC, Ortonne N, Ortonne JP, Schedl A. 2005. Sox9 is essential for outer root sheath differentiation and the formation of the hair stem cell compartment. *Curr Biol* **15**: 1340-1351.

Zhang X, Liu S, Wang Y, Hu H, Li L, Wu Y, Cao D, Cai Y, Zhang J, Zhang X. 2019. Interleukin22 regulates the homeostasis of the intestinal epithelium during inflammation. *Int J Mol Med* **43**: 1657-1668.

Development • Supplementary information
