# Supplementary material for: NF-κB determines Paneth versus goblet cell fate decision in the small intestine
Source: Development. 2021 Nov 9;148(21):dev199683. doi: 10.1242/dev.199683 (PMC8627599; doi:10.1242/dev.199683)
Supplement: Reviewer comments [file develop-148-199683_review_history.pdf]

NF-κB determines Paneth versus goblet cell fate decision in the small intestine

Cristina Brischetto, Karsten Krieger, Christian Klotz, Inge Krahn, Séverine Kunz, Marina Kolesnichenko, Patrick Mucka, Julian Heuberger, Claus Scheidereit and Ruth Schmidt-Ullrich
DOI: 10.1242/dev.199683

Editor: James Wells

Review timeline

Original submission:	6 April 2021
Editorial decision:	12 May 2021
First revision received:	31 August 2021
Accepted:	28 September 2021

Original submission

First decision letter

MS ID#: DEVELOP/2021/199683

MS TITLE: NF-κB determines Paneth versus goblet cell fate decision in the small intestine

AUTHORS: Cristina Brischetto, Karsten Krieger, Christian Klotz, Inge Krahn, Severine Kunz, Marina Kolesnichenko, Patrick Michael Mucka, Julian Heuberger, Claus Scheidereit, and Ruth Schmidt-Ullrich

I have now received all the referees reports on the above manuscript, and have reached a decision. The referees' comments are appended below, or you can access them online: please go to BenchPress and click on the 'Manuscripts with Decisions' queue in the Author Area.

The overall evaluation is positive and we would like to publish a revised manuscript in Development, provided that the referees' comments can be satisfactorily addressed. Please attend to all of the reviewers' comments in your revised manuscript and detail them in your point-by-point response. If you do not agree with any of their criticisms or suggestions explain clearly why this is so.

We are aware that you may currently be unable to access the lab to undertake experimental revisions. If it would be helpful, we encourage you to contact us to discuss your revision in greater detail. Please send us a point-by-point response indicating where you are able to address concerns raised (either experimentally or by changes to the text) and where you will not be able to do so within the normal timeframe of a revision. We will then provide further guidance. Please also note that we are happy to extend revision timeframes as necessary.

Reviewer 1

Advance summary and potential significance to field

The manuscript characterized the suppression of Paneth cell lineage commitment in a previously published mouse model with ubiquitous inhibition of NF-κB signalling.

© 2021. Published by The Company of Biologists under the terms of the Creative Commons Attribution License (https://creativecommons.org/licenses/by/4.0/).

With NF-κB activity reporter, the authors identified the unique NF-κB activity restricted to Paneth cells and occasionally CBCs. Upon ubiquitous suppression of NF-κB signalling, it was found that the overall proliferation and cell death IECs remained unchanged, while Paneth/goblet cell differentiation was dysregulated. The authors further discussed the essentiality of cell-intrinsic NF-κB activity for IECs homeostasis by regulating Paneth cell maturation through SOX9 expression. Overall, the manuscript provided a spectrum of phenotypic characterization in IECs upon ubiquitous NF-κB inhibition.

Comments for the author

One major concern regarding to this study is that, in the mouse model chosen (ΔN), the expression cassette of degradation-resistant IκB mutants (IκBαΔN) is within the β-catenin locus, accompanied by heterozygous β-catenin knockout. Thus, this genotype makes it difficult to rule out the synergistic effect of NF-κB inhibition together with β-catenin heterozygosity to the phenotypes described. Meanwhile, since the expression level of IκBαΔN is under control of β-catenin promoter, it is likely inducing an artificial negative feedback between β-catenin dosage and NF-κB activity, adding another layer of complexity on potential signalling crosstalk. Unfortunately, this point was not discussed in the manuscript. In Figure 3, the authors observed increased numbers of goblet cells in both ΔN and IEC-restricted villin-ΔN mice, but this phenotype was not reported in previous other IEC- restricted IKK/NF-κB deletion. Meanwhile, the authors characterized the profound loss of Paneth cells at the ΔN crypts. Since Paneth cell differentiation relies on high Wnt/β-catenin activity, it is possible that the heterozygous β-catenin mutant could also contribute to the goblet/Paneth cell phenotypes described. Moreover, in Figure 7G, the western blot showed that there could be a reduction (quantification missing) of nuclear β-catenin in ΔN duodenum, where the most drastic loss of Paneth cells was described. Thus, a key piece of information missing is that whether Wnt/β-catenin/Lgr5 signaling is affected by β-catenin heterozygosity in intestine. In this case, loxP-ΔN mouse line can be recruited as additional control, which also contains heterozygous β-catenin mutant but with intact loxP-stop-loxP preventing IκBαΔN expression. The authors should quantify the nuclear β-catenin and Wnt targets expression, in all three genotypes (wild-type, loxP-ΔN and ΔN) to understand the possible combinatory effect of NF-κB inhibition and β-catenin heterozygosity. Further validation in a wild-type genotype would be helpful to exclude the possible signalling crosstalk, for instance, wild-type organoids with treatment of selective IKKβ inhibitors shall provide direct evidence to attribute the Paneth/goblet cell phenotype to NF-κB inhibition.

Minor Critiques:

In Figure 2A and B, the authors speculated that the Ki67/BrdU positive cells at crypt base in ΔN line are likely TA cells/immature cell types migrating down to the crypt, however, these cells could also be proliferating stem cells. It is intriguing to know whether suppression of NF-κB signalling altered TA/CBC ratio, and how does that affect the morphology of crypt- villus axis (cell density per um, crypt/villus length)? In Figure 2B, the quantification of crypt- villus axis is missing, though it is mentioned in the figure legend.

In Figure S5, CBC markers Lgr5, Ascl2 and Olfm4 was examined in ΔN versus wild-type control (Lgr5-EGFP). Apparently, only Wnt-regulated CBC marker (Lgr5 and Ascl2) was downregulated, but not non-Wnt-regulated marker (Olfm4), suggesting that the Wnt/β-catenin/LGR5 signaling is compromised in ΔN CBCs. It is known that ablation of Paneth cells or epithelial cell-derived Wnt does not cause Lgr5+ stem cell depletion in the crypts, and Notch signalling still remains active. Can authors provide possible explanation for the decrease of Lgr5+ CBCs and the decreased expression of Dll1 and Hes1 in ΔN crypts? The crippled immune system resulted from ubiquitous NF-κB inhibition is likely to affect the stromal niche for Paneth/stem cell homeostasis. To distinguish whether the compromised Wnt/β-catenin/LGR5 is due to crypt-intrinsic NF-κB inhibition or extrinsic defects, the author could include the IEC-restricted villin-ΔN mice for comparison.

© 2021. Published by The Company of Biologists under the terms of the Creative Commons Attribution License (https://creativecommons.org/licenses/by/4.0/).

In organoid culture experiment (Figure 6 and S6), it seems that exogenous Wnt partially rescue the NF-κB inhibition phenotype. However, the ΔN organoid morphology remains different from control. Since this rescue experiment was performed in Lgr5-EGFP; ΔN reporter line, besides Lgr5 mRNA expression, a quantification of the Lgr5 positive cells would be helpful to understand whether the Lgr5+ stem cell population is restored. To confirm that the ΔN-driven phenotypes are cell-intrinsic, authors should perform long-term organoid culture, instead of one-week culture, to assess whether ΔN can be maintained in exogenous Wnt condition for multiple passages. Since Lgr5+ CBCs deficiency was observed in vivo, how exogenous Wnt condition, but not stroma Wnt, can restore Lgr5+ CBCs should be discussed. Figure S6 B-E, figure legends are mislabelled.

In Figure 7, the authors pointed that the central transcription factor Sox9 regulating Paneth cell maturation is downregulated by NF-κB inhibition, reflecting differentiation defects rather than cell death. However, intestinal goblet cell mucin, Muc2, is also a downstream target of canonical NF-κB signalling, while presenting overexpression phenotype in ΔN mice. Thus, the mechanism underlying Sox9 regulation upon NF-κB inhibition required more examination, and how NF-κB responsive genes are differentially affected by ΔN should be discussed.

The persistent activity of intrinsic NF-κB in Paneth cell shown both in vivo and in vitro is novel and worth further investigation/discussion. According to the reporter activity, NF-κB signalling is uniquely activated in mature Paneth cells and some CBCs, indicating its other potential role in maintenance of cell function. The authors could examine the effect of acute inhibition of NF-κB activity in Paneth cell by using Defensin-CreERT mice, or by treating wild- type ENR organoids with NF-κB inhibitors. The NF-κB activity presented in the subset of CBCs is also worth noting. Is this unique CBC subset patterned to become Paneth cell? If using a Lgr5-Cre mouse line to ablate such population also results in loss of Paneth cells, it would provide further evidence that NF-κB plays a crucial upstream for Paneth cell commitment.

In short, the ΔN mouse model presented in this study does provide a non-inflammatory background to study the role of intrinsic NF-κB in intestinal epithelial homeostasis. However, since the major phenotype (ablation of Paneth, decreased CBCs) implicated crosstalk between NF-κB with Wnt/β-catenin signalling, the authors shall provide evidence that the Wnt/β-catenin signalling in intestine is not compromised by β-catenin heterozygosity in ΔN background, and that the phenotype discovered is not a combinatory effect of ubiquitous NF-κB inhibition and β-catenin heterozygosity.

Reviewer 2

Advance summary and potential significance to field

Understanding the function of NF-κB in intestinal epithelial homeostasis, beyond its well-established role in intestinal inflammation, is of great interest to the fields of intestinal stem cell (ISC) and developmental biology. In this study, Brischetto et al. sought to examine NF-κB physiological role in murine small intestine (SI) by 1) using NF-κB reporter mouse lines and 2) assessing the role of NF-κB in Paneth vs. goblet cell fate decisions in crypt organoids (mini-guts). The authors use mouse lines with β-galactosidase (κ-Gal) or EGFP (κ-EGFP) expression as read-outs for NF-κB activity, which was shown to be primarily confined to crypt ISCs (potential secretory progenitors) in the +4/+5 position as well as in Paneth cells. Additionally, mouse models are used with ubiquitous suppression of NF-κB activity (ΔN) or with intestinal epithelial cell-restricted NF-κB inhibition (Villin-ΔN) to demonstrate that suppression of NF-κB activity does not change overall proliferation rate or cell death in vivo but reduces viable organoids. However, NF-κB suppression (ΔN) appears to increase numbers of goblet cells and reduce numbers of Paneth cells both in vivo and in mini-guts compared to WT controls. While the findings about the role of NF-κB in Paneth and goblet cell differentiation are novel and important, not all of the claims regarding the role of NF-κB in cell proliferation/ISC homeostasis are fully supported by the data, additionally some statements

© 2021. Published by The Company of Biologists under the terms of the Creative Commons Attribution License (https://creativecommons.org/licenses/by/4.0/).

appear to be contradictory and need to be clarified or further explored. The findings are important in understanding the mechanisms underlying intestinal stem cell self-renewal and differentiation.

Comments for the author

Comments:

1. A major critique is that, in the results section, the authors state that NF-κB activity was identified "also at position +4/+5 and occasionally as Lgr5+ CBCs" in reference to Fig. 1C without actually staining for or otherwise identifying Lgr5+ cells in the figure. It appears that they use the ISC marker Bmi1 as a proxy for Lgr5, but they are functionally distinct and should not be used interchangeably.

2. Results state that lack of NF-κB activity in ΔN mice was verified by loss of IκBα mRNA expression (Fig. S1A), yet later in the discussion they state that "in ΔN mice, NF-κB activity is not completed blocked," this appears contradictory and should be clarified earlier in the manuscript.

3. Results state that the impaired ex vivo ability to culture organoids may be due to Paneth cell ablation in ΔN epithelium, but then in the discussion it is stated that "in ΔN mice, NF-κB activity is not completely blocked…which allows for the formation of occasional Paneth cells." Given the conclusion that "Paneth cells are the main producers of Wnt and Notch ligands," it confounds the studies on ENR vs. WENR media.

4. In order to claim that proliferation was reduced in organoids derived from ΔN mice (Fig. 6) but not in vivo, a more direct assay would have been to stain organoids with Ki67 (as done in histological sections in Fig. 2) and quantify proliferation as number of Ki67+ cells rather than viable organoids or crypts/well.

5. It is well known that Y-27632 promotes proliferation and self-renewal of stem cells, such that organoid studies assessing proliferation often include Y-27632 in crypt culture medium within the first 24 hours of passage but then remove it from the media prior to proliferation studies. However, it appears that the authors always include Y-27632 in their organoid culture media. This could confound the conclusions and should be evaluated.

6. A minor comment is that, under EXPERIMENTAL PROCEDURES line 29 (under Organoid cultures), it should read "Dissociated cells were passed through a cell strainer" rather than "passed through cell strainer".

First revision

<u>Author response to reviewers' comments</u>

We thank the expert reviewers for their careful analysis of our manuscript and the positive and constructive comments. We have responded to all of the reviewer's points with additional data and/or discussion. All changes done in the manuscript are marked in yellow.

Reviewer 1

One major concern regarding to this study is that, in the mouse model chosen (ΔN), the expression cassette of degradation-resistant IκB mutants (IκBαΔN) is within the β-catenin locus, accompanied by heterozygous β-catenin knockout. Thus, this genotype makes it difficult to rule out the synergistic effect of NF-κB inhibition together with β-catenin heterozygosity to the phenotypes described. Meanwhile, since the expression level of IκBαΔN is under control of β-catenin promoter, it is likely inducing an artificial negative feedback between β-catenin dosage and NF-κB activity, adding another layer of complexity on potential signalling crosstalk. Unfortunately, this point was not discussed in the manuscript.

© 2021. Published by The Company of Biologists under the terms of the Creative Commons Attribution License (https://creativecommons.org/licenses/by/4.0/).

We thank the reviewer for the suggestion to illustrate our mouse model. The ΔN mouse model was first published and explained in detail in 2001 (see (Schmidt-Ullrich et al. 2001)). Note that β-catenin expression is regulated at the protein level (protein stability), not at the transcriptional level. In the 2001 article, we showed β-catenin protein expression in mouse embryonic fibroblasts obtained from ΔN, floxed ΔN (loxP-ΔN) and wildtype control mice and did not observe any difference (see (Schmidt-Ullrich et al. 2001) and Fig. 1E therein). We also compared expression levels of β-catenin protein in all major tissues from ΔN, loxP-ΔN and control mice and did not see any variation at all (data was not shown). Importantly, loxP-ΔN mice do not have any phenotype and are thus identical to wildtype control mice (Schmidt- Ullrich et al. 2001). This would not be expected if one-allelic Ctnnb1 expression would have led to compromised β-catenin expression. Finally, homozygous KO of β-catenin is lethal, however, heterozygous IκBαΔN expression from only one Ctnnb1 allele turned out to be abundantly sufficient to suppress NF-κB activity.

In Figure 3, the authors observed increased numbers of goblet cells in both ΔN and IEC- restricted villin-ΔN mice, but this phenotype was not reported in previous other IECrestricted IKK/NF-κB deletion. Meanwhile, the authors characterized the profound loss of Paneth cells at the ΔN crypts. Since Paneth cell differentiation relies on high Wnt/β-catenin activity, it is *possible that the heterozygous β-catenin mutant could also contribute to the goblet/Paneth cell phenotypes described.*

*The reason why IEC-specific IKK/NF-κB KOs do not recapitulate the ΔN phenotype (loss of Paneth cells concomitant with increased numbers of goblet cells) is mainly due to inflammation going on in these KOs. The same phenomenon was, incidentally, also observed in mice with epidermal loss of NF-κB activity (Makris et al. 2000; Schmidt-Supprian et al. 2000; Pasparakis et al. 2002; Stratis et al. 2006a; Stratis et al. 2006b; Rebholz et al. 2007). Generally, in epithelium-specific IKK/NF-κB KOs the observed recruitment of inflammatory cells and cytokine production in the underlying mesenchyme results in apoptosis of cells in the intestinal epithelium (for instance of Paneth cells; (Nenci et al. 2007; Vlantis et al. 2016)), or in epidermal keratinocytes and hair follicles. Thus, phenotypes observed in IEC- or skin- specific IKK/NF-κB KO mice are rather related to (or are overshadowed by) massive inflammation going on in the underlying mesenchyme which causes high levels of inflammatory cytokine production. In mice with ubiquitous suppression of NF-κB (ΔN), inflammatory processes are blocked (see **Suppl. Fig. S1**), as these require NF-κB activity in immune cells, including local dendritic cells and macrophages in the adjacent mesenchyme. Therefore, ΔN mice are helpful to analyze intrinsic NF-κB functions in tissues independently of any aberrant immune reactions. As expected, IEC-restricted Villin-ΔN mice (Villin-ΔN: loxP-ΔN x villin-Cre) also showed some inflammation at advanced ages, but at 8 weeks of age, the phenotype was identical to ΔN mice (**Fig. S2B** and **Fig. 4A**). Finally, it was shown in recent years that the IKK complex also regulates other signaling pathways and cellular processes, which is why IKK inhibition does not necessarily reflect NF-κB functions (see for instance (Vlantis et al. 2016; Mikuda et al. 2018)).*

Downregulation of Wnt/β-catenin in secretory precursors is indeed needed for generation of goblet cells. Thus, if β-catenin would not be expressed at appropriate levels in ΔN mice, secretory precursors could also preferentially form goblet cells instead of Paneth cells. As this is not the case in our mouse model, we hypothesize that NF-κB is required for the Paneth/goblet cell switch in secretory precursors. This might occur via specific upregulation of Wnt10a by NF-κB in secretory precursors to generate Paneth cells. If this is blocked, goblet cells will form instead. However, the exact mechanism remains unknown and will be analyzed in more detail in the future by single cell analysis.

Moreover, in Figure 7G, the western blot showed that there could be a reduction (quantification missing) of nuclear β-catenin in ΔN duodenum, where the most drastic loss of Paneth cells was described. Thus, a key piece of information missing is that whether Wnt/β- catenin/Lgr5 signaling is affected by β-catenin heterozygosity in intestine. In this case, loxP- ΔN mouse line can be recruited as additional control, which also contains heterozygous β- catenin mutant but with intact loxP-stop-loxP preventing IκBαΔN expression. The authors should quantify the nuclear β-catenin and Wnt targets expression, in all three genotypes (wild- type, loxP-ΔN and ΔN) to understand the possible combinatory effect of NF-κB inhibition and β-catenin heterozygosity. Further validation in a wild-type genotype would be helpful to exclude the possible signaling crosstalk, for instance, wild-type organoids with treatment of selective IKKβ inhibitors shall provide direct evidence to

© 2021. Published by The Company of Biologists under the terms of the Creative Commons Attribution License (https://creativecommons.org/licenses/by/4.0/).

attribute the Paneth/goblet cell phenotype to NF-κB inhibition.

*We thank the reviewer for these suggestions and have now added a Western blot and quantification of β-catenin protein expression in the SI epithelium comparing ΔN, loxP-ΔN and control mice to again confirm that there is no difference in β-catenin protein expression between these 2 lines and the controls (see new **Suppl. Fig. S1B and C**). We also added IF images to show that β-catenin expression is not altered in SI tissue or in SI crypt organoids of ΔN or control mice (see below, **Fig. 1 for reviewer**). As β-catenin forms an important part of the adherens junctions (as seen in **Fig. 1 for reviewer**), these were also closely examined in the EM images of our current study (see Fig. 5). Again, we could not see any difference in the number and appearance of adherens junctions when comparing ΔN and control mice (data not shown).*

As expected, a knock-out mouse model of the canonical NF-κB inhibitor IκBα in IEC which has constitutively elevated NF-κB activity in the intestinal epithelium (IκBα$^{IEC\text{-}KO}$; (Mikuda et al. 2020)) showed an opposed phenotype to ΔN mice: When using organoids of IκBα$^{IEC\text{-}KO}$ to overcome the IBD-like inflammation seen in these mice and to analyze intrinsic effects of elevated NF-κB activity in IEC, increased numbers of misplaced Paneth cells and of CBCs as well as elevated Wnt activity were observed (Mikuda et al. 2020).

We hope that we were able to answer these important points to the reviewer's full satisfaction, as for reasons of space we are not able to discuss the ΔN mouse model in any more detail in our manuscript.

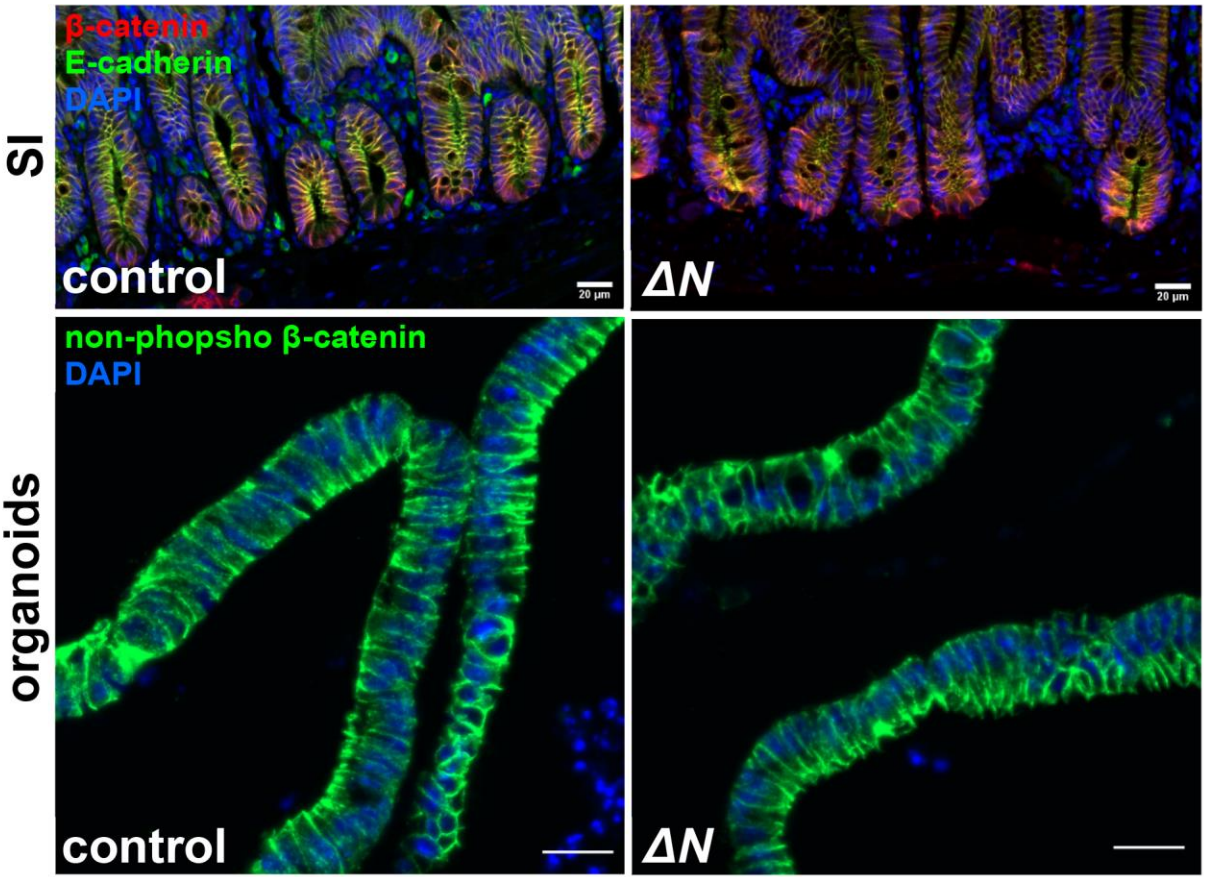

__Fig. 1 for reviewers__: Upper panels: IF on small intestinal (SI) tissue sections of ΔN and control mice using antibodies against E-cadherin and non-phospho (active) β-catenin (Ser33/37/Thr41). Nuclear staining: DAPI. Scale bars = 20 µm. Lower panels: IF on crypt organoids from ΔN and control mice using an antibody that binds non-phospho (active) β- catenin (Ser33/37/Thr41). Nuclear staining: DAPI. Scale bars = 20 µm.

© 2021. Published by The Company of Biologists under the terms of the Creative Commons Attribution License (https://creativecommons.org/licenses/by/4.0/).

Minor Critiques:

In Figure 2A and B, the authors speculated that the Ki67/BrdU positive cells at crypt base in ΔN line are likely TA cells/immature cell types migrating down to the crypt, however, these cells could also be proliferating stem cells. It is intriguing to know whether suppression of NF-κB signalling altered TA/CBC ratio, and how does that affect the morphology of cryptvillus axis (cell density per um, crypt/villus length)?

*We thank the reviewer for this suggestion. We have calculated the TA/CBC ratio (percentage of Ki67/Lgr5$^+$ cells) and found that in ΔN mice it is significantly higher when compared to controls (see **new Fig. S5H**). The same was observed in crypt organoids grown in the absence of Wnt (ENR; see **Fig. 2 for reviewers** below). This is in line with our finding of reduced Lgr5 expression (see **Fig. S5**) which strongly indicates a reduction in Lgr5$^+$ CBCs in ΔN mice and a role for NF-κB in CBC maintenance.*

*Except for altered cell compositions, we did not observe any other gross crypt morphology changes when comparing ΔN and control mice. The overall Ki67-positive cell numbers also remained unaltered in ΔN mice (see **new Fig. 2B**). However, Lgr5$^+$ cells are also Ki67- positive which is why the overall number of proliferative cells in crypts of ΔN (mainly CBC progenitors (TA)/secretory progenitors) and of controls (Lgr5$^+$ CBC, and further up Lgr5$^+$ CBC progenitors including secretory precursors and TA cells) is similar (**new Fig. 2B**).*

In Figure 2B, the quantification of cryptvillus axis is missing, though it is mentioned in the figure legend.

*We thank the reviewer for indicating the error in Fig. 2B, now **right panel of Fig. 2D**. In the right panel (24h after BrdU injection), it should indeed say "BrdU$^+$ cells/crypt-villus axis". This has been corrected.*

In Figure S5, CBC markers Lgr5, Ascl2 and Olfm4 was examined in ΔN versus wild-type control (Lgr5-EGFP). Apparently, only Wnt-regulated CBC marker (Lgr5 and Ascl2) was downregulated, but not non-Wnt-regulated marker (Olfm4), suggesting that the Wnt/β- catenin/LGR5 signaling is compromised in ΔN CBCs.

*We thank the reviewer for this hint. We now analyzed mRNA expression of various ISC markers (from (van der Flier et al. 2009b; Mikuda et al. 2020)) in light of Wnt dependency (see **new Fig. S5 D and E**). Indeed, with the exception of Ephb3, Msi1 and Prom1, which also mark secretory precursors, Wnt-dependent ISC markers Lgr5, Ascl2, Ccnd1, Edn1 and Tnfrsf19 (Troy) were significantly downregulated in ΔN mice, which suggests that Wnt signaling is compromised in ΔN mice (**Fig. S5E**). Wnt-independent ISC markers Lgr1, Smoc2, Tert, Hopx and Olfm4 remained unaltered between ΔN and controls (**Fig. S5D**).*
We hypothesize that NF-κB regulates expression of specific Wnts (we show significantly reduced expression of Wnt3 and Wnt10a in ΔN mice), particularly in secretory precursors, which would be required for generation of Paneth cells, but probably also in Paneth cells which would be essential for CBC maintenance.

It is known that ablation of Paneth cells or epithelial cell-derived Wnt does not cause Lgr5+ stem cell depletion in the crypts, and Notch signalling still remains active. Can authors provide possible explanation for the decrease of Lgr5+ CBCs and the decreased expression of Dll1 and Hes1 in ΔN crypts?

Various publications suggest that early, direct progenitors of Lgr5$^+$ CBCs show high Dll1 expression (Stamataki et al. 2011; van Es et al. 2012). These Dll1^{high} cells are only supposed to be progenitors for the secretory lineages and thus for Paneth cells. On one hand, these particular Lgr5$^+$ cells which would end up differentiating into Dll1^{high} progenitors might be missing in ΔN mice. This would result in decreased Dll1 expression.
On the other hand, Dll1 is also expressed on Paneth cells (Pinto et al. 2003; van Es et al. 2005).

© 2021. Published by The Company of Biologists under the terms of the Creative Commons Attribution License (https://creativecommons.org/licenses/by/4.0/).

Therefore, reduced Dll1 expression concomitant with diminished expression of Notch target gene Hes1 might additionally arise from loss of Paneth cells in ΔN mice.

The crippled immune system resulted from ubiquitous NF-κB inhibition is likely to affect the stromal niche for Paneth/stem cell homeostasis. To distinguish whether the compromised Wnt/ß-catenin/LGR5 is due to crypt-intrinsic NF-κB inhibition or extrinsic defects, the author could include the IEC-restricted villin-ΔN mice for comparison.

*We thank the reviewer for the suggestion. Please note that in **Fig. 3A and B, 4A-C and S2B** IEC-restricted Villin-ΔN mice were used which show an identical phenotype to ΔN mice regarding loss of Paneth cells and increased goblet cells. This confirms that the ISC phenotype observed in the SI crypts of ΔN mice is not related to a crippled immune system. Furthermore, T cell depletion leads to an increase in ISCs (Biton et al. 2018) which is opposite to what we observe in ΔN mice. Finally, SI crypt organoids generated from mice with constitutively **increased** NF-κB activity in the SI epithelium (IκBa$^{IEC\text{-}KO}$; (Mikuda et al. 2020)) showed an increased number of Lgr5- positive CBCs concomitant with increased Wnt activity.*

In organoid culture experiment (Figure 6 and S6), it seems that exogenous Wnt partially rescue the NF-κB inhibition phenotype. However, the ΔN organoid morphology remains *different from control. Since this rescue experiment was performed in Lgr5-EGFP; ΔN reporter line, besides Lgr5 mRNA expression, a quantification of the Lgr5 positive cells would be helpful to understand whether the Lgr5+ stem cell population is restored.*

*We thank the reviewer for the suggestion. We calculated the Ki67/Lgr5^{+} cell ratio in ΔN and control organoids grown in the absence (ENR) or presence of Wnt3 (WENR) and show that Lgr5 expression is re-established when adding exogenous Wnt to the culture medium (see **Fig. 2 for reviewers**). This suggests that the Lgr5^{+} CBC population is restored in ΔN organoids in presence of Wnt. This also explains why ΔN organoids are able to grow in WENR medium.*

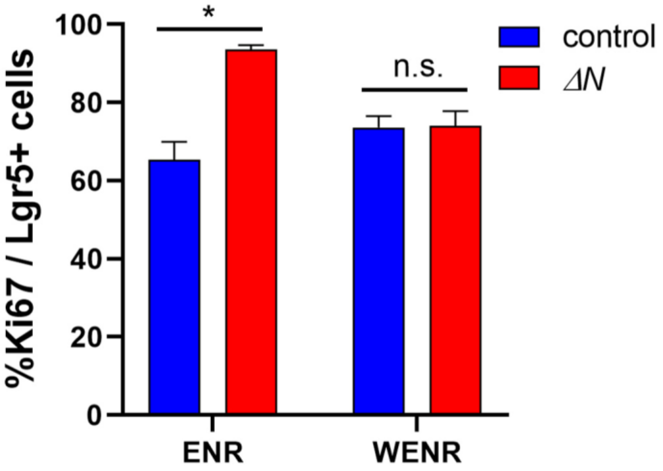

***Fig. 2 for reviewers**: Quantification of Ki67 versus Lgr5 expression on crypt organoids grown in absence (ENR) or presence of Wnt3 ligand (WENR) for 14 days (3rd passage) and stained with antibodies against Ki67 and Lgr5. Two-way Analysis of variance (ANOVA) with Bonferroni´s multiple comparison test, *: $p<0.05$, n.s.= not significant, error bars = SEM.*

To confirm that the ΔN-driven phenotypes are cell-intrinsic, authors should perform long term organoid culture, instead of one-week culture, to assess whether ΔN can be maintained in exogenous Wnt condition for multiple passages.

*We did not observe any changes in long-term cultures of ΔN organoids in WENR medium (3 passages, 14 days; see **Fig. 2 for reviewers**). Thus, ΔN organoids can be maintained in exogenous Wnt medium for multiple passages.*

© 2021. Published by The Company of Biologists under the terms of the Creative Commons Attribution License (https://creativecommons.org/licenses/by/4.0/).

Since Lgr5+ CBCs deficiency was observed in vivo, how exogenous Wnt condition, but not stroma Wnt, can restore Lgr5+ CBCs should be discussed.

We would like to point out to the reviewer, that we hypothesize in the discussion that there are particular Wnts, here Wnt10a, that are specifically expressed in Paneth cells, but not in the mesenchymal stroma. Thus, stromal Wnts may not be sufficient to fully generate all Lgr5^{+} CBCs in crypts of ΔN mice. Furthermore, it was shown that early progenitors from CBCs (in our case TA cells in the ΔN crypts) can revert to Lgr5^{+} CBCs in presence of high Wnt concentrations (van Es et al. 2012), which would be the case for organoids grown in WENR medium.
Further analyses are planned in the future to pursue and prove this hypothesis.

Figure S6 B-E, figure legends are mislabelled.

We apologize for this error which has been corrected accordingly.

In Figure 7, the authors pointed that the central transcription factor Sox9 regulating Paneth cell maturation is downregulated by NF-κB inhibition, reflecting differentiation defects rather than cell death. However, intestinal goblet cell mucin, Muc2, is also a downstream target of canonical NF-κB signalling, while presenting overexpression phenotype in ΔN mice. Thus, the mechanism underlying Sox9 regulation upon NF-κB inhibition required more examination, and how NF-κB responsive genes are differentially affected by ΔN should be discussed.

We thank the reviewer for this suggestion. We did not detect any NF-κB activity in mature goblet cells of the small intestine which suggests that Muc2 is not a direct NF-κB target gene in these cells under normal conditions, at least not in the small intestine. This may be different during inflammatory bowel disease when NF-κB is aberrantly activated due to inflammation, in particular in colon (colitis). Thus far, Muc2 was only described as a target gene of NF-κB in connection with inflammatory processes (infections etc.) in epithelia, such as colon and airways (see f.ex. (Jono et al. 2002; Lee et al. 2002)). Furthermore, Muc2 expression was not altered in SI tissue of IκBa$^{IEC\text{-}KO}$ mice (Mikuda et al. 2020). Thus, the observed increase in Muc2 in ΔN mice is simply due to the elevated number of goblet cells.

*It was shown that Sox9 is required for Paneth cell formation (Mori-Akiyama et al. 2007). Furthermore, we have shown previously that Sox9 is a direct NF-κB target gene (Tomann et al. 2016). Thus, lack of Sox9 expression in crypts of ΔN mice is likely due to suppressed NF-κB activity. However, we have now added quantifications of mRNA expression of various known NF-κB target genes comparing ΔN to IκBa$^{IEC\text{-}KO}$ mice (**new Fig. 7E**, as well as **S1E and F**; wildtype controls were set to 1). As expected, these results confirm that all NF-κB targets analyzed are downregulated in ΔN mice, while aberrantly upregulated in IκBa$^{IEC\text{-}KO}$ mice.*

The persistent activity of intrinsic NF-κB in Paneth cell shown both in vivo and in vitro is novel and worth further investigation/discussion. According to the reporter activity, NF-κB signalling is uniquely activated in mature Paneth cells and some CBCs, indicating its other potential role in maintenance of cell function. The authors could examine the effect of acute inhibition of NF-κB activity in Paneth cell by using Defensin-CreERT mice, or by treating wildtype ENR organoids with NF-κB inhibitors. The NF-κB activity presented in the subset of CBCs is also worth noting. Is this unique CBC subset patterned to become Paneth cell? If using a Lgr5-Cre mouse line to ablate such population also results in loss of Paneth cells, it would provide further evidence that NF-κB plays a crucial upstream for Paneth cell commitment.

We thank the reviewer for these very interesting suggestions some of which will be pursued in the future, in particular cell-tracing experiments to analyze the destiny of CBCs with NF-κB activity, as well as breeding of Lgr5-Cre and loxP-ΔN mice. Unfortunately, this would be too time-consuming for the rebuttal, because we currently do not breed the corresponding mouse models for doing these studies. We also believe that it would be beyond the scope of the current manuscript.

Unfortunately, there are no specific NF-κB inhibitors, and NF-κB has functions which are

© 2021. Published by The Company of Biologists under the terms of the Creative Commons Attribution License (https://creativecommons.org/licenses/by/4.0/).

independent of IKK, which is why the use of IKK inhibitors might not inhibit NF-κB activity (Kolesnichenko et al. 2021). Frequently used inhibitors, like BAY 11-7085 and Bardoxolone for instance, mainly inhibit the IKK complex. However, IKK inhibitors are known to inhibit NF-κB-independent functions of the IKK complex (for example (Vlantis et al. 2016; Mikuda et al. 2018)) and to cause apoptosis of epithelial cells. In line with this, we observed massive apoptosis when adding BAY 11-7085 or Bardoxolone to our organoid culture medium. We also treated control organoids in ENR medium with Glucocorticoids which come closest to a proper NF-κB inhibitor. However, it is known that this only functions after prior treatment with an NF-κB activator, such as TNFα. Unfortunately, this dual treatment did not results in any changes (i.e. loss of Paneth cells, increased goblet cells) in control organoids.

In short, the ΔN mouse model presented in this study does provide a non-inflammatory background to study the role of intrinsic NF-κB in intestinal epithelial homeostasis. However, since the major phenotype (ablation of Paneth, decreased CBCs) implicated crosstalk between NF-κB with Wnt/β-catenin signalling, the authors shall provide evidence that the Wnt/β- catenin signalling in intestine is not compromised by β-catenin heterozygosity in ΔN background, and that the phenotype discovered is not a combinatory effect of ubiquitous NF- κB inhibition and β-catenin heterozygosity.

We hope that we were able to convince the reviewer that loss of Paneth cells, decreased CBCs and increased numbers of goblet cells observed in ΔN mice are distinctly related to suppressed NF-κB activity in the SI epithelium and not due to reduced β-catenin expression that could, of course, have potentially arisen from the knock-in of the truncated IκBa cDNA into the ctnnb1 locus in our ΔN mouse model. This mouse model was already successfully used in studying the development of ectodermal appendages, such as hair follicles, teeth or mammary glands. They all require early Wnt signaling for inducing their formation which was not impaired in ΔN mice. Yet, any Wnt signaling that was required downstream of EDA- A1/NF-κB signaling in the development of ectodermal appendages was inhibited in these mice (see (Schmidt-Ullrich et al. 2006; Liu et al. 2008; Zhang et al. 2008; Zhang et al. 2009; Haara et al. 2011; Voutilainen et al. 2012; Voutilainen et al. 2015)).

Reviewer 2

Understanding the function of NF-κB in intestinal epithelial homeostasis, beyond its well-established role in intestinal inflammation, is of great interest to the fields of intestinal stem cell (ISC) and developmental biology. In this study, Brischetto et al. sought to examine NF- κB physiological role in murine small intestine (SI) by 1) using NF-κB reporter mouse lines and 2) assessing the role of NF-κB in Paneth vs. goblet cell fate decisions in crypt organoids (mini-guts). The authors use mouse lines with β-galactosidase (κ-Gal) or EGFP (κ-EGFP) expression as read-outs for NF-κB activity, which was shown to be primarily confined to crypt ISCs (potential secretory progenitors) in the +4/+5 position as well as in Paneth cells. Additionally, mouse models are used with ubiquitous suppression of NF-κB activity (ΔN) or with intestinal epithelial cell-restricted NF-κB inhibition (Villin-ΔN) to demonstrate that suppression of NF-κB activity does not change overall proliferation rate or cell death in vivo but reduces viable organoids. However, NF-κB suppression (ΔN) appears to increase numbers of goblet cells and reduce numbers of Paneth cells both in vivo and in mini-guts compared to WT controls. While the findings about the role of NF-κB in Paneth and goblet cell differentiation are novel and important, not all of the claims regarding the role of NF-κB in cell proliferation/ISC homeostasis are fully supported by the data, additionally some statements appear to be contradictory and need to be clarified or further explored. The findings are important in understanding the mechanisms underlying intestinal stem cell self- renewal and differentiation.

1. A major critique is that, in the results section, the authors state that NF-κB activity was identified “also at position +4/+5 and occasionally as Lgr5+ CBCs” in reference to Fig. 1C without actually staining for or otherwise identifying Lgr5+ cells in the figure. It appears that they use the ISC marker Bmi1 as a proxy for Lgr5, but they are functionally distinct and should not be used interchangeably.

We thank the reviewer for this comment. We agree that Bmi1 was not an optimal choice. We now

© 2021. Published by The Company of Biologists under the terms of the Creative Commons Attribution License (https://creativecommons.org/licenses/by/4.0/).

*stained SI tissue sections of κ-EGFP mice with pan-stem cell marker Olfm4 which also marks Lgr5$^+$ CBCs (van der Flier et al. 2009a) and see a clear overlap of EGFP (NF-κB activity) and Olfm4 expression. The Bmi1 image in **Fig. 1C** was thus exchanged with EGFP/Olfm4 staining.*

We are aware that our judgment of ISCs with NF-κB activity is vague and was mainly judged by position. Regarding +4/+5 cells, there are many debates going on about the role of these cells which may actually vary between crypts and the momentary need of particular cell types, as well as other factors. Single cell sequencing and cell-tracing experiments will be needed to identify the role of NF-κB in the respective ISC type. This is planned for the future.
As expected, the phenotype observed in ΔN mice (suppressed NF-κB activity) is the opposite of mice with constitutively increased NF-κB activity in the intestinal epithelium (IκBa$^{IEC\text{-}KO}$; (Mikuda et al. 2020)): In IκBa$^{IEC\text{-}KO}$ organoids expression of Lgr5 as well as numbers of Lgr5$^+$ CBCs and Paneth cells were increased. Unfortunately, we were not able to analyze NF-κB activity in Lgr5-EGFP mice, because both reporters use EGFP as read-out. However, Lgr5$^+$ CBCs are located in the bottom of the crypt and not at position +4/+5.

2. Results state that lack of NF-κB activity in ΔN mice was verified by loss of IκBα mRNA expression (Fig. S1A), yet later in the discussion they state that "in ΔN mice, NF-κB activity is not completed blocked," this appears contradictory and should be clarified earlier in the manuscript.
*We apologize for this misunderstanding. Note that a complete block of NF-κB activity is embryonic lethal. IKK/NF-κB KO mice in which NF-κB activity is inhibited 100% die around E14/E15, mainly due to strongly increased hepatocyte apoptosis in the liver, in which hematopoiesis is taking place at that time. 25% of ΔN-positive embryos also die at E14/E15 (see (Schmidt-Ullrich et al. 2001)). However, 75% survive and helped to discover important novel functions of NF-κB besides regulation of the immune response, cell cycle regulation and survival, inflammation and oncogenesis: in the development and morphogenesis of ectodermal appendages such as hair follicles, teeth, mammary glands, secondary lymph nodes, as well as self-renewal of hair follicles and Paneth cells. In the text, we refer to ΔN mice as having suppressed NF-κB activity instead of blocked activity, which most certainly would result in total absence of Paneth cells. However, suppression of NF-κB activity by expression of the trans-dominant super-repressor IκBaΔN is potent enough to allow loss of Paneth cells, complete downregulation of bona fide NF-κB target gene IκBa and strongly reduced expression of other known target genes such as Sox9, A20 (Tnfaip3) and IBD markers Nos2, Nox1, Lcn2 and Lbp (see **Fig. 7E** and new **Fig. S1F**). These latter targets were now examined in relation to controls (set at 1) and IκBa$^{IEC\text{-}KO}$ mice (**Fig. 7E** and new **Fig. S1F**).*

3. Results state that the impaired ex vivo ability to culture organoids may be due to Paneth cell ablation in ΔN epithelium, but then in the discussion it is stated that "in ΔN mice, NF-κB activity is not completely blocked…which allows for the formation of occasional Paneth cells." Given the conclusion that "Paneth cells are the main producers of Wnt and Notch ligands," it confounds the studies on ENR vs. WENR media.
*Please see our response to point 2 and **Reviewer 1** explaining that ΔN mice are not a complete NF-κB KO mouse model which would be embryonic lethal. ΔN organoids derived from crypts without Paneth cells (which is the vast majority) do not grow in ENR medium, because Paneth cells are required to produce Wnt and induce Notch activation in CBCs which are essential for CBC maintenance. Those few organoids generated from crypts that do have a Paneth cell (see also Fig. 4) are able to grow in ENR medium. This is the reason why we also examined organoid formation using single isolated ΔN-positive Lgr5$^+$ CBCs (see Fig. 6). Furthermore, we did not keep the organoids in culture long enough to allow potential overgrowth with those very few organoids which would have a Paneth cell and would eventually grow normally.*
We have now altered this part in the discussion to avoid misunderstandings.

4. In order to claim that proliferation was reduced in organoids derived from ΔN mice (Fig. 6) but not in vivo, a more direct assay would have been to stain organoids with Ki67 (as done in histological sections in Fig. 2) and quantify proliferation as number of Ki67+ cells rather than viable organoids or crypts/well.
We thank the reviewer for the suggestion. We now have calculated the percentage of Ki67-positive cells versus unstained cells (DAPI) comparing crypt organoids generated from ΔN or

© 2021. Published by The Company of Biologists under the terms of the Creative Commons Attribution License (https://creativecommons.org/licenses/by/4.0/).

*control mice (see **new Fig. S6F**). The results demonstrate that in the absence of Wnt (ENR medium, black bars), Ki67-positive cell numbers are significantly reduced in ΔN mice when compared to controls (p<0.0001), which is rescued in medium containing Wnt3 (WERN, grey bars; **new Fig. S6F**). These data confirm our results shown in **Fig. 6B**.*

5. It is well known that Y-27632 promotes proliferation and self-renewal of stem cells, such that organoid studies assessing proliferation often include Y-27632 in crypt culture medium within the first 24 hours of passage but then remove it from the media prior to proliferation studies. However, it appears that the authors always include Y-27632 in their organoid culture media. This could confound the conclusions and should be evaluated.

We thank the reviewer for the comment and we apologize for the inaccuracy. In fact, we only used Y-27632 when generating organoids from FACS-sorted single Lgr5-positive cells obtained from SI crypts. Sorted cells were then cultured for 24 hours in medium containing 10 µm Y27632. After 24 hours, the ROCK inhibitor was removed and cells were grown in medium without Y-27632. Analyses were performed 4 – 7 days later. Thus, it is very unlikely that the ROCK inhibitor still had any influence on our results. We have now corrected this part in the Experimental Procedures.

6. A minor comment is that, under EXPERIMENTAL PROCEDURES line 29 (under Organoid cultures), it should read “Dissociated cells were passed through a cell strainer” rather than “passed through cell strainer”.
We apologize for this error and have corrected it accordingly.

<u>Literature for Reviewers</u>:

Biton M, Haber AL, Rogel N, Burgin G, Beyaz S, Schnell A, Ashenberg O, Su CW, Smillie C, Shekhar K et al. 2018. T Helper Cell Cytokines Modulate Intestinal Stem Cell Renewal and Differentiation. *Cell* **175**: 1307-1320 e1322.

Haara O, Fujimori S, Schmidt-Ullrich R, Hartmann C, Thesleff I, Mikkola ML. 2011. Ectodysplasin and Wnt pathways are required for salivary gland branching morphogenesis. *Development* **138**: 2681-2691.

Jono H, Shuto T, Xu H, Kai H, Lim DJ, Gum JR, Jr., Kim YS, Yamaoka S, Feng XH, Li JD. 2002. Transforming growth factor-beta -Smad signaling pathway cooperates with NF-kappa B to mediate nontypeable Haemophilus influenzae-induced MUC2 mucin transcription. *J Biol Chem* **277**: 45547-45557.

Kolesnichenko M, Mikuda N, Hopken UE, Kargel E, Uyar B, Tufan AB, Milanovic M, Sun W, Krahn I, Schleich K et al. 2021. Transcriptional repression of NFKBIA triggers constitutive IKK- and proteasome-independent p65/RelA activation in senescence. *EMBO J* **40**: e104296.

Lee HW, Ahn DH, Crawley SC, Li JD, Gum JR, Jr., Basbaum CB, Fan NQ, Szymkowski DE, Han SY, Lee BH et al. 2002. Phorbol 12-myristate 13-acetate up-regulates the transcription of MUC2 intestinal mucin via Ras, ERK, and NF-kappa B. *J Biol Chem* **277**: 32624-32631.

Liu F, Chu EY, Watt B, Zhang Y, Gallant NM, Andl T, Yang SH, Lu MM, Piccolo S, Schmidt- Ullrich R et al. 2008. Wnt/beta-catenin signaling directs multiple stages of tooth morphogenesis. *Dev Biol* **313**: 210-224.

Makris C, Godfrey VL, Krahn-Senftleben G, Takahashi T, Roberts JL, Schwarz T, Feng L, Johnson RS, Karin M. 2000. Female mice heterozygous for IKK gamma/NEMO deficiencies develop a dermatopathy similar to the human X-linked disorder incontinentia pigmenti [In Process Citation]. *Mol Cell* **5**: 969-979.

Mikuda N, Kolesnichenko M, Beaudette P, Popp O, Uyar B, Sun W, Tufan AB, Perder B, Akalin A, Chen W et al. 2018. The IkappaB kinase complex is a regulator of mRNA stability. *EMBO J* **37**.

Mikuda N, Schmidt-Ullrich R, Kargel E, Golusda L, Wolf J, Hopken UE, Scheidereit C, Kuhl AA, Kolesnichenko M. 2020. Deficiency in IkappaBalpha in the intestinal epithelium leads to spontaneous inflammation and mediates apoptosis in the gut. *J Pathol* **251**: 160-174.

Mori-Akiyama Y, van den Born M, van Es JH, Hamilton SR, Adams HP, Zhang J, Clevers H, de Crombrugghe B. 2007. SOX9 is required for the differentiation of paneth cells in the intestinal epithelium. *Gastroenterology* **133**: 539-546.

© 2021. Published by The Company of Biologists under the terms of the Creative Commons Attribution License (https://creativecommons.org/licenses/by/4.0/).

Nenci A, Becker C, Wullaert A, Gareus R, van Loo G, Danese S, Huth M, Nikolaev A, Neufert C, Madison B et al. 2007. Epithelial NEMO links innate immunity to chronic intestinal inflammation. *Nature* **446**: 557-561.

Pasparakis M, Courtois G, Hafner M, Schmidt-Supprian M, Nenci A, Toksoy A, Krampert M, Goebeler M, Gillitzer R, Israel A et al. 2002. TNF-mediated inflammatory skin disease in mice with epidermis-specific deletion of IKK2. *Nature* **417**: 861-866.

Pinto D, Gregorieff A, Begthel H, Clevers H. 2003. Canonical Wnt signals are essential for homeostasis of the intestinal epithelium. *Genes Dev* **17**: 1709-1713.

Rebholz B, Haase I, Eckelt B, Paxian S, Flaig MJ, Ghoreschi K, Nedospasov SA, Mailhammer R, Debey-Pascher S, Schultze JL et al. 2007. Crosstalk between keratinocytes and adaptive immune cells in an IkappaBalpha protein-mediated inflammatory disease of the skin. *Immunity* **27**: 296-307.

Schmidt-Supprian M, Bloch W, Courtois G, Addicks K, Israel A, Rajewsky K, Pasparakis M. 2000. NEMO/IKK gamma-deficient mice model incontinentia pigmenti. *Mol Cell* **5**: 981-992.

Schmidt-Ullrich R, Aebischer T, Hulsken J, Birchmeier W, Klemm U, Scheidereit C. 2001. Requirement of NF-kappaB/Rel for the development of hair follicles and other epidermal appendices. *Development* **128**: 3843-3853.

Schmidt-Ullrich R, Tobin DJ, Lenhard D, Schneider P, Paus R, Scheidereit C. 2006. NF-kappaB transmits Eda A1/EdaR signalling to activate Shh and cyclin D1 expression, and controls post-initiation hair placode down growth. *Development* **133**: 1045-1057.

Stamataki D, Holder M, Hodgetts C, Jeffery R, Nye E, Spencer-Dene B, Winton DJ, Lewis J. 2011. Delta1 expression, cell cycle exit, and commitment to a specific secretory fate coincide within a few hours in the mouse intestinal stem cell system. *PLoS One* **6**: e24484.

Stratis A, Pasparakis M, Markur D, Knaup R, Pofahl R, Metzger D, Chambon P, Krieg T, Haase I. 2006a. Localized inflammatory skin disease following inducible ablation of I kappa B kinase 2 in murine epidermis. *J Invest Dermatol* **126**: 614-620.

Stratis A, Pasparakis M, Rupec RA, Markur D, Hartmann K, Scharffetter-Kochanek K, Peters T, van Rooijen N, Krieg T, Haase I. 2006b. Pathogenic role for skin macrophages in a mouse model of keratinocyte-induced psoriasis-like skin inflammation. *J Clin Invest* **116**: 2094-2104.

Tomann P, Paus R, Millar SE, Scheidereit C, Schmidt-Ullrich R. 2016. Lhx2 is a direct NF- kappaB target gene that promotes primary hair follicle placode down-growth. *Development* **143**: 1512-1522.

van der Flier LG, Haegebarth A, Stange DE, van de Wetering M, Clevers H. 2009a. OLFM4 is a robust marker for stem cells in human intestine and marks a subset of colorectal cancer cells. *Gastroenterology* **137**: 15-17.

van der Flier LG, van Gijn ME, Hatzis P, Kujala P, Haegebarth A, Stange DE, Begthel H, van den Born M, Guryev V, Oving I et al. 2009b. Transcription factor achaete scute-like 2 controls intestinal stem cell fate. *Cell* **136**: 903-912.

van Es JH, Sato T, van de Wetering M, Lyubimova A, Yee Nee AN, Gregorieff A, Sasaki N, Zeinstra L, van den Born M, Korving J et al. 2012. Dll1+ secretory progenitor cells revert to stem cells upon crypt damage. *Nat Cell Biol* **14**: 1099-1104.

van Es JH, van Gijn ME, Riccio O, van den Born M, Vooijs M, Begthel H, Cozijnsen M, Robine S, Winton DJ, Radtke F et al. 2005. Notch/gamma-secretase inhibition turns proliferative cells in intestinal crypts and adenomas into goblet cells. *Nature* **435**: 959-963.

Vlantis K, Wullaert A, Polykratis A, Kondylis V, Dannappel M, Schwarzer R, Welz P, Corona T, Walczak H, Weih F et al. 2016. NEMO Prevents RIP Kinase 1-Mediated Epithelial Cell Death and Chronic Intestinal Inflammation by NF-kappaB-Dependent and -Independent Functions. *Immunity* **44**: 553-567.

Voutilainen M, Lindfors PH, Lefebvre S, Ahtiainen L, Fliniaux I, Rysti E, Murtoniemi M, Schneider P, Schmidt-Ullrich R, Mikkola ML. 2012. Ectodysplasin regulates hormone-independent mammary ductal morphogenesis via NF-kappaB. *Proc Natl Acad Sci U S A* **109**: 5744-5749.

Voutilainen M, Lindfors PH, Trela E, Lonnblad D, Shirokova V, Elo T, Rysti E, Schmidt-Ullrich R, Schneider P, Mikkola ML. 2015. Ectodysplasin/NF-kappaB Promotes Mammary Cell Fate via Wnt/beta-catenin Pathway. *PLoS Genet* **11**: e1005676.

Zhang Y, Andl T, Yang SH, Teta M, Liu F, Seykora JT, Tobias JW, Piccolo S, Schmidt-Ullrich R, Nagy A et al. 2008. Activation of {beta}-catenin signaling programs embryonic epidermis to hair follicle fate. *Development* **135**: 2161 - 2172.

Zhang Y, Tomann P, Andl T, Gallant NM, Huelsken J, Jerchow B, Birchmeier W, Paus R, Piccolo

© 2021. Published by The Company of Biologists under the terms of the Creative Commons Attribution License (https://creativecommons.org/licenses/by/4.0/).

S, Mikkola ML et al. 2009. Reciprocal requirements for EDA/EDAR/NF-kappaB and Wnt/beta-catenin signaling pathways in hair follicle induction. *Dev Cell* **17**: 49-61.

Second decision letter

MS ID#: DEVELOP/2021/199683

MS TITLE: NF-κB determines Paneth versus goblet cell fate decision in the small intestine

AUTHORS: Cristina Brischetto, Karsten Krieger, Christian Klotz, Inge Krahn, Severine Kunz, Marina Kolesnichenko, Patrick Michael Mucka, Julian Heuberger, Claus Scheidereit, and Ruth Schmidt-Ullrich
ARTICLE TYPE: Research Article

I am happy to tell you that your manuscript has been accepted for publication in Development, pending our standard ethics checks.

Reviewer 1

Advance summary and potential significance to field

In this study, utilising degradation-resistant IκB mutants, Brischetto et al. examined the cell-intrinsic NF-κB activity in Paneth cell, and its role in murine intestinal epithelial homeostasis. It is of great interest to the fields of intestinal epithelial. It is worth further pursuing the role of persistent NF-κB activity not only in Paneth cells, but also in subtypes of intestinal stem cells.

Comments for the author

In the revised version of the manuscript, the authors manage to address the major concern regarding to the ΔN mouse model chosen that whether Wnt/ß-catenin/Lgr5 signaling is affected by ß-catenin heterozygosity in this model. Additional western blot quantification of nuclear ß-catenin from additional controls are included in the Figure S1 and additional staining of ß-catenin are provided in the rebuttal, indicating that ß-catenin activity is not compromised. For the minor critiques, additional quantifications are included in Figure 2, and additional gene makers are assayed as requested in Figure S5. The major claim of the paper (“NF-kB activity is required for proper Paneth cell differentiation and for SI epithelial homeostasis”) has been more clearly supported by the additional controls. From my point of view, the point-by-point responses provided by the authors adequately addresses the concerns raised, and the paper is now suitable for publication in Development.

Reviewer 2

Advance summary and potential significance to field

Novel role for NF-kB in Paneth lineage allocation in the small intestine.

Comments for the author

All my critiques were adequately addressed.

© 2021. Published by The Company of Biologists under the terms of the Creative Commons Attribution License (https://creativecommons.org/licenses/by/4.0/).
